# The MBNL1/circNTRK2/PAX5 pathway regulates aerobic glycolysis in glioblastoma cells by encoding a novel protein NTRK2-243aa

**DOI:** 10.1038/s41419-022-05219-4

**Published:** 2022-09-05

**Authors:** Yubo Zhao, Jian Song, Weiwei Dong, Xiaobai Liu, Chunqing Yang, Di Wang, Yixue Xue, Xuelei Ruan, Libo Liu, Ping Wang, Mengyang Zhang, Yunhui Liu

**Affiliations:** 1 Department of Neurosurgery, Shengjing Hospital of China Medical University, Shenyang, 110004 China; 2 Key Laboratory of Neuro-oncology in Liaoning Province, Shenyang, 110004 China; 3 Liaoning Medical Surgery and Rehabilitation Robot Technology Engineering Research Center, Shenyang, 110004 China; 4 Department of Neurobiology, School of Life Sciences, China Medical University, Shenyang, 110122 China

**Keywords:** Cancer metabolism, Phosphorylation, Oncogenes

## Abstract

Glioblastoma multiforme (GBM) is the most common tumor of the human central nervous system. Aerobic glycolysis has been strongly related to tumor development and malignant behavior. In this study, we found that MBNL1, circNTRK2, and NTRK2-243aa were markedly downregulated and inhibited glycolysis in GBM, whereas PAX5 was upregulated and promoted glycolysis. Functionally, MBNL1 promoted the expression of circNTRK2 by binding to *NTRK2* pre-mRNA, as validated using RNA pull-down and nascent RNA immunoprecipitation assays. Mass spectrometry, western blotting, and immunofluorescence staining methods were used to detect the expression of NTRK2-243aa. NTRK2-243aa—encoded by circNTRK2—phosphorylated PAX5 at Y102, leading to the attenuation of the half-life of PAX5, as validated by in vitro kinase and MG132 rescue assays. Besides, PAX5 transcriptionally facilitated the expression of PKM2 and HK2 by binding to their promoter regions, as verified by luciferase reporter and chromatin immunoprecipitation assays. Finally, overexpression of MBNL1 and circNTRK2 combined with PAX5 knockdown effectively inhibited the formation of GBM xenograft tumors and significantly prolonged the survival of orthotopic nude mice. We have delineated that the MBNL1/circNTRK2/PAX5 pathway plays a crucial role in regulating GBM glycolysis and could provide potential targets and alternative strategies for the treatment of GBM.

## Introduction

Brain glioblastoma (GBM) is the most common primary malignant tumor of the central nervous system [1], characterized by high aggressiveness, mortality, recurrence rate, and poor prognosis [2, 3]. The median survival of patients with GBM is only approximately 15 months [4, 5]. Recently, an increasing number of studies have indicated that aerobic glycolysis can be an effective target for multiple cancers treatment, including GBM, colon cancer, and breast cancer [6, 7]. Aerobic glycolysis, also called Warburg effect, is considered as one of the top 10 characteristics of tumors [8, 9]. Therefore, it is crucial to elucidate molecular mechanisms of aerobic glycolysis in GBM to further explore reliable biomarkers.

RNA-binding proteins (RBPs) are a class of proteins that specifically bind to RNA and regulate a series of processes [10, 11]. Some RBPs are involved in critical steps in cancers. For instance, HNF4A-AS1 binds to hnRNPU to transactivate CTCF, increasing glycolysis in neuroblastoma cells [12]. Muscleblind-like splicing regulator 1 (MBNL1), one of the members of the MBNL protein family, has been negatively correlated with poor prognosis in breast, lung, and gastric cancers, and inhibits the recurrence and metastasis of triple-negative breast cancer [13]. MBNL1 is downregulated in breast cancer, leukemia, stomach cancer, esophageal cancer, glioma, and Huntington’s disease [14].

Circular RNAs (circRNAs) are endogenous non-coding RNAs that lack poly adenylation and capping with properties of high stability, universality, conservation, and tissue-specificity [15]. In bladder cancer, circACVR2A is positively correlated with clinicopathological invasion features. Besides, circACVR2A regulates ETA4 expression, thus inhibiting malignant behavior of bladder cancer cells [16]. Likewise, neurotrophic receptor tyrosine kinase 2 (NTRK2) is associated with poor prognosis in lung and colorectal cancer [17]. Recently, Britton et al. reported that in pediatric cerebellar glioblastoma, NTRK2 acts as a fusion oncogenic driver via oncogenomic analysis [18]. By scanning the circBase database, it was found that hsa_circ_0139157 with a length of 728 nt, called circNTRK2 in this study, might be formed from the backsplicing of *NTRK2* pre-mRNA. Recently, several studies have focused on the potentially essential regulatory role of circRNA-encoded proteins in tumorigenesis and development [19]. For instance, AKT3-174aa, encoded by circAKT3, regulates proliferation ability and radiation resistance of glioma cells [20]. Additionally, circRNAs with integrated RNA translation components such as internal ribosome entry site (IRES) and open reading frame (ORF) are translated into polypeptides [21]. The circNTRK2, identified in our study, has both IRES and ORF components, possibly encoding a polypeptide consisting of 243 amino acids, NTRK2-243aa. However, few studies have focused on circNTRK2 and NTRK2-243aa.

The expression of Paired box 5 (PAX5) was increased in a range of astrocytoma [22]. In addition, PAX5 transcriptionally activates the expression of FOXP4-AS1 and FOXP4, exerting tumor-promotive effects [23]. Hexokinase 2 (HK2) and pyruvate kinase M2 (PKM2), the rate-limiting enzymes of cellular glycolysis, are remarkably upregulated in glioma, colorectal cancer, and liver cancer, promoting glycolysis and have been generally accepted as key factors in the reprogramming of glucose metabolism [24–26].

## Results

### MBNL1 was downregulated in GBM cells and inhibited glycolysis and proliferation upon overexpression

**Fig. 1:**
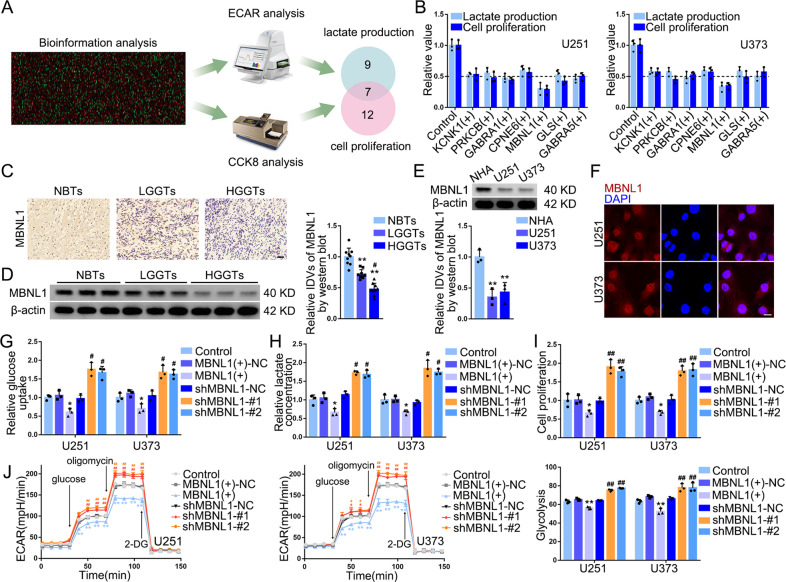
Expression and Effect of MBNL1 in GBM Tissues and Cells after Identification of MBNL1 as a Glycolytic Suppressor. **A** Experimental scheme for identifying suppressors potentially related with both cell glycolysis and proliferation. **B** Lactate production and cell proliferation of U251 and U373 overexpressing 7 suppressors respectively. Data are presented as mean ± SD (*n* = 3, each group). **C** IHC shows the expression and distribution of MBNL1 in NBTs (normal brain tissues), LGGTs, HGGTs (×200; scale bar represents 150 μm). **D** NBTs deriving from regions adjacent to the GBM and GBM tissues of different grades (LGGTs, HGGTs) were analyzed for MBNL1 protein levels by western blotting. IDVs of the bands were statistically analyzed. Data are manifested as mean ± SD (*n* = 9, each group). **P < 0.01, compared with NBTs group; ^#^*P* < 0.05 compared with LGGTs group. **E** Expression of MBNL1 in NHA, U251 and U373 cells. IDVs of the bands were statistically analyzed. Data are manifested as mean ± SD (*n* = 3, each group). **P < 0.01 compared to NHA group. **F** The distribution of MBNL1 in U251 and U373 cells was determined by IF assay (MBNL1 was labeled in red by secondary antibody against anti-MBNL1 antibody and nuclei were labeled in blue by DAPI. Scale bar represents 10 µm.). **G**–**I** Effects of overexpression or knockdown of MBNL1 on glucose uptake, lactate production, and proliferation of U251 and U373 cells were detected by glucose uptake, lactate, and cell proliferation assay respectively. Data are presented as mean ± SD (*n* = 3, each group). *P < 0.05 compared with MBNL1(+)-NC group; ^#^*P* < 0.05, ^##^*P* < 0.01 compared with shMBNL1-NC group. **J** Effects of MBNL1 overexpression or knockdown on ECAR in U251 and U373 cells were determined by XF24 Extracellular Flux Analyzer and glycolysis levels in each ECAR group were analyzed. Data are presented as mean ± SD (*n* = 3, each group), **P < 0.01 compared with MBNL1(+)-NC group; ^#^*P* < 0.05, ^##^*P* < 0.01 compared with shMBNL1-NC group. Glycolytic capacity and glycolytic reserve in each ECAR group were analyzed (see Supplementary Fig. 1D, E).

Using the cancer genome atlas (TCGA) database, we identified suppressors that were expressed in much lower levels in GBM and ranked these suppressors according to their log2-fold change. We overexpressed the top 50 suppressors in GBM cells, respectively, and detected lactate production and cell proliferation of these cells. 16 suppressors might be related to lactate production, 19 involved in cell proliferation, and 7 potentially correlated with both (Fig. 1A, Supplementary Fig. 1A, Supplementary File 1). Interestingly, among these seven suppressors, MBNL1 overexpression reduced lactate production and cell proliferation by more than 50% (Fig. 1B). We then evaluated the diagnostic value of *MBNL1* mRNA using the receiver operating characteristic (ROC) curve with the area under the curve (AUC) being 0.872 (Supplementary Fig. 1B). TCGA database analysis indicated both mutation of *IDH* and 1p/19q codeletion were correlated with MBNL1 expression (Supplementary Table 1). *MBNL1* mRNA expression was significantly lower in the five GBM cell lines than normal human astrocytes (NHA) (Supplementary Fig. 1C) and we selected U251 and U373 for future studies. MBNL1 protein levels in glioma tissues were significantly decreased and negatively correlated with pathological grades (Fig. 1C, D). Moreover, we detected a downregulation of MBNL1 in GBM cells (Fig. 1E). Immunofluorescence (IF) assays showed that MBNL1 was distributed in the nucleus (Fig. 1F). Besides, MBNL1 overexpression markedly decreased glycolysis and proliferation (Fig. 1G–J). Among glucose metabolism-related proteins, the mRNA levels of HK2 and PKM2 were prominently decreased upon MBNL1 overexpression (Supplementary Fig. 1F), suggesting that HK2 and PKM2 might be the targets through which MBNL1 regulates glycolysis. Meanwhile, we found MBNL1 overexpression reduced the expression of HK2 and PKM2 proteins (Supplementary Fig. 1G). The glycolysis inhibitor, 2-DG, could partially reverse the proliferation-promoting effect exerted by MBNL1 knockdown (Supplementary Fig. 1H).

### CircNTRK2, downregulated in GBM tissues and cells, restrained glycolysis and proliferation in GBM cells

**Fig. 2:**
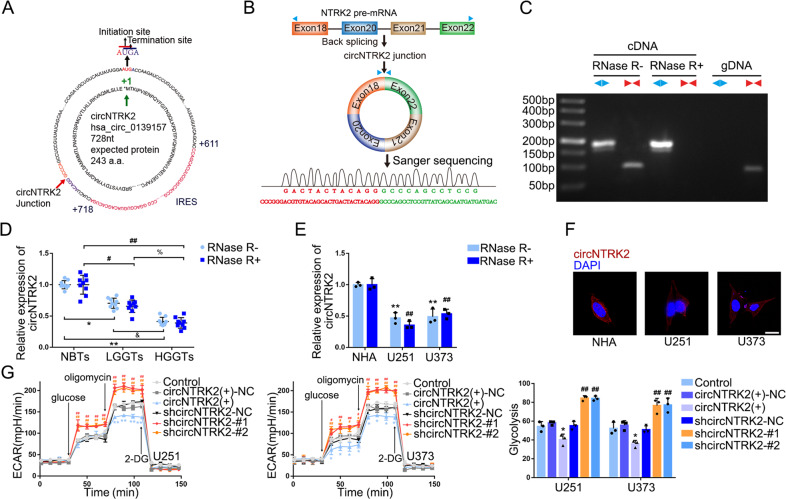
CircNTRK2 was Downregulated in GBM Tissues and Cells, inhibiting Glycolysis and Proliferation in GBM Cells. **A** Scheme for circNTRK2 sequence information including junction sequence, IRES, and ORF, as well as NTRK2-243aa sequence potentially encoded. **B** Scheme illustrates *NTRK2* pre-mRNA was back-spliced to form circNTRK2 and the junction sequence was specifically amplified following Sanger sequencing using diverge primers. **C** CircNTRK2 was detected in cDNA and gDNA by qRT-PCR with or without RNase R treatment (diverge primers in blue and converge primers in red). **D** Expression level of circNTRK2 in NBTs, LGGTs, and HGGTs. Data are manifested as mean ± SD (*n* = 9, each group). *P < 0.05, **P < 0.01, compared with RNase R- NBTs group; ^#^*P* < 0.05, ^##^*P* < 0.01 compared with RNase R + NBTs group; ^&^*P* < 0.05 compared with RNase R- LGGTs group; ^%^*P* < 0.05 compared with RNase R + LGGTs group. **E** Expression level of circNTRK2 in U251 and U373 treated with or without RNase R. Data are manifested as mean ± SD (*n* = 3, each group). There was no significant difference between RNase R- and RNase R + groups in NHA, U251, and U373 cells. **P < 0.01, compared with RNase R- NHA group; ^##^*P* < 0.01 compared with RNase R + NHA group. **F** Distribution of circNTRK2 in NHA, U251, and U373 was determined by FISH. Scale bar represents 10 µm. **G** Effects of circNTRK2 overexpression or knockdown on ECAR in U251 and U373 cells were determined and glycolysis levels in each ECAR group were analyzed. Data are presented as mean ± SD (*n* = 3, each group). *P < 0.05, **P < 0.01 compared with circNTRK2(+)-NC group; ^#^*P* < 0.05, ^##^*P* < 0.01 compared with shcircNTRK2-NC group. Glycolytic capacity and glycolytic reserve in each ECAR group were analyzed (see Supplementary Fig. 2I, J).

To elucidate the mechanism through which MBNL1 regulates aerobic glycolysis in GBM cells, we employed circRNA microarrays in GBM cells overexpressing MBNL1 (Supplementary Fig. 2A). Among top 10 upregulated circRNAs, we found that the expression of hsa_circ_0139157 (circNTRK2) exhibited a remarkable increase (Supplementary Fig. 2B). Meanwhile, we did not observe any difference in the linear NTRK2 expression (Supplementary Fig. 2C). Therefore, we reasoned that MBNL1 might inhibit glycolysis and proliferation via circNTRK2. We proceeded to analyze the expression and function of circNTRK2 in GBM cells. Figure 2A illustrates the structure of circNTRK2, providing essential information. We designed divergent primers to amplify the junction region of circNTRK2 as shown in Fig. 2B. Besides, circNTRK2 was detected in cDNA, but not in gDNA (Fig. 2C). We further used RNase R to confirm the relative expression of circNTRK2. CircNTRK2 was significantly reduced in GBM tissues and cells, and negatively correlated with grades (Fig. 2D, E). Whereas the level of linear NTRK2 was markedly decreased after treatment with RNase R (Supplementary Fig. 2D). We then observed circNTRK2 was localized in the cytoplasm (Fig. 2F). We detected a prominent reduction in glucose uptake, lactate production, extracellular acidification rate (ECAR), and proliferation of GBM cells following circNTRK2 overexpression, without affecting the expression of linear NTRK2 (Fig. 2G and Supplementary Fig. 2E–H). Moreover, we found that circNTRK2 overexpression decreased HK2 and PKM2 proteins levels (Supplementary Fig. 2K). 2-DG could partially reverse the proliferation-promoting effect exerted by circNTRK2 knockdown (Supplementary Fig. 2L).

### MBNL1 regulated glycolysis and proliferation in GBM cells through circNTRK2

**Fig. 3:**
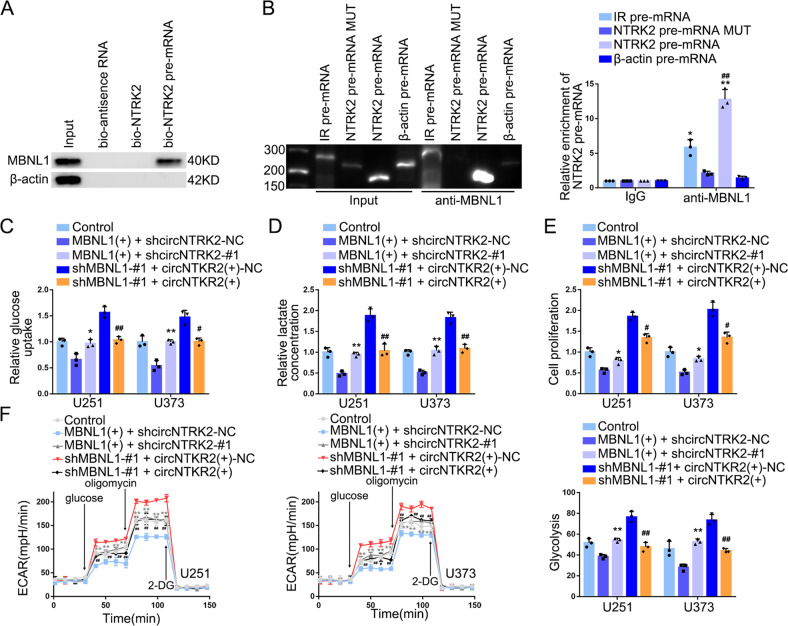
MBNL1 promoted CircNTRK2 expression by binding to *NTRK2* Pre-mRNA inhibiting glycolysis and proliferation in GBM cells. **A** The combination of MBNL1 and *NTRK2* pre-mRNA was determined by RNA pull-down assays. MBNL1 and β-actin protein in immunoprecipitation with bio-*NTRK2* pre-mRNA were detected via western blotting. Bio-antisense RNA was referred as negative control. **B** The relative enrichment of *NTRK2* pre-mRNA in IgG and anti-MBNL1 group was detected by nascent RIP assay. *IR* pre-mRNA group was referred as positive control whereas *β-actin* pre-mRNA group as negative control. Data are presented as mean ± SD (*n* = 3, each group). *P < 0.05, **P < 0.01 compared with anti-MBNL1 *β-actin* pre-mRNA group; ^##^*P* < 0.01 compared with anti-MBNL1 *NTRK2* pre-mRNA MUT group. **C**–**E** The reverse effect of circNTRK2 on MBNL1 in terms of glucose uptake, lactate production, and proliferation. Data are presented as mean ± SD (*n* = 3, each group). *P < 0.05, **P < 0.01 compared with MBNL1(+) + shcircNTRK2-NC group; ^#^*P* < 0.05, ^##^*P* < 0.01 compared with shMBNL1-#1 + circNTRK2(+)-NC group. **F** The reverse effect of circNTRK2 on MBNL1 in terms of glycolysis and glycolysis levels in each ECAR group were analyzed. Data are presented as mean ± SD (*n* = 3, each group). **P < 0.01 compared with MBNL1(+) + shcircNTRK2-NC group; ^##^*P* < 0.01 compared with shMBNL1-#1 + circNTRK2(+)-NC group. Glycolytic capacity and glycolytic reserve in each ECAR group were analyzed (see Supplementary Fig. 3C, D).

Using RNA pull-down and nascent RIP assays, we further clarified the relationship between MBNL1 and *NTRK2* pre-mRNA (Fig. 3A, B). We selected the insulin receptor pre-mRNA as a positive control in nascent RIP assays [27]. We found the plasmid carrying 100 bases of the flanking introns failed to produce circNTRK2 (Supplementary Fig. 3A). Besides, MBNL1 overexpression did not alter the half-life of circNTRK2 (Supplementary Fig. 3B). Furthermore, we observed that knocking-down circNTRK2 rescued the inhibitory effect exerted by MBNL1 overexpression (Fig. 3C–F). In addition, we found the circNTRK2 knockdown restored the inhibition of the HK2 and PKM2 proteins induced by MBNL1 overexpression (Supplementary Fig. 3E). Taken together, these results show that MBNL1 regulates the expression of HK2 and PKM2 by promoting circNTRK2 biosynthesis and not via improving stability.

### NTRK2-243aa, encoded by circNTRK2, exerted inhibitory effects on glycolysis and proliferation in GBM cells

**Fig. 4:**
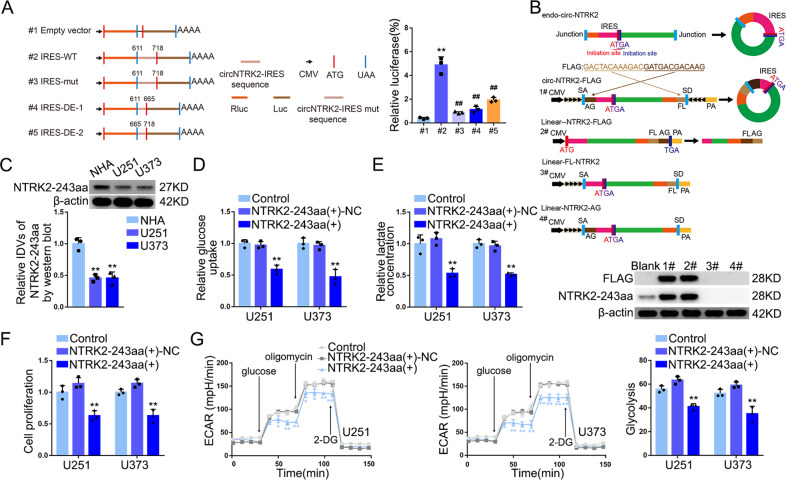
NTRK2-243aa, encoded by CircNTRK2, inhibited glycolysis and proliferation of GBM cells. **A** The activity of IRES (611–718) in circNTRK2 was tested. Left panel shows circNTRK2 IRES (611–718) wild type, mutant type, and two truncated types were cloned between Luc and Rluc with independent promoter and terminator on both sides respectively. Right panel shows Dual-Luciferase Reporter Assay kit determined Luc/Rluc relative luciferase activity in HEK-293T cells transfected with vectors on the left. Data are presented as mean ± SD (*n* = 3, each group). **P < 0.01 compared with empty vector group. ^##^*P* < 0.01 compared with IRES (611–718) WT group. **B** The coding ability of ORF in circNTRK2 was tested. Endo-circ-NTRK2: scheme showing how endogenous circNTRK2 formed with circular junction inside the ORF forming a unique sequence shown in orange. Circ-NTRK2-FLAG: exon 18, exon 20, exon 21, and exon 22 of NTRK2 were cloned between splicing acceptor (SA), splicing donor (SD), and side flanking sequences (black arrows). FLAG tag was divided into both sides (dark brown and light brown). cyclization of this vector could form the same circular RNA as endogenous circNTRK2, except for the FLAG tag behind the ORF. Linear-NTRK2-FLAG: the 243aa ORF with FLAG tag directly cloned behind a linear expression vector. Linear-FL-NTRK2 and Linear-NTRK2-AG: circ**-**NTRK2-FLAG vector lacking the flanking sequence. The expression levels of FLAG and NTRK2-243aa in HEK-293T cell upon transfection of vectors above were analyzed by FLAG antibody and customized anti-NTRK2-243aa antibody (see Supplementary Fig. 4C). **C** Relative expression level of NTRK2-243aa in U251 and U373 was detected. Data are manifested as mean ± SD (*n* = 3, each group). IDVs of the bands were statistically analyzed. **P < 0.01, compared with NHA group. **D**–**F** Effects of NTRK2-243aa knockdown on glucose uptake, lactate production, and proliferation in U251 and U373 cells were determined by glucose uptake, lactate, and cell proliferation assays respectively. Data are presented as mean ± SD (*n* = 3, each group). **P < 0.01, compared with NTRK2-243aa(+)-NC group. **G** Effects of NTRK2-243aa overexpression on ECAR in U251 and U373 cells were measured and glycolysis levels in each ECAR group were analyzed. Data are presented as mean ± SD (*n* = 3, each group). *P < 0.05, **P < 0.01 compared with NTRK2-243aa(+)-NC group. Glycolytic capacity and glycolytic reserve in each ECAR group were analyzed (see Supplementary Fig. 4G, H).

Investigation of circRNADb (http://reprod.njmu.edu.cn/cgi-bin/circrnadb/circRNADb.php) revealed the presence of ORF and IRES components in circNTRK2 that probably facilitate the synthesis of a polypeptide consisting of 243 amino acids via using an “AUGA” overlapping start-stop codon (Supplementary Fig. 4A). We did not detect any significant difference in the relative Luc/RLuc activity between the wild-type IRES (328–478) and empty vector groups (Supplementary Fig. 4B). We also tested the activity of IRES (611–718), its mutant type, and truncated types (Fig. 4A), to verify the activity of the predicted IRES (611–718). Next, we validated the ORF encoding ability in circNTRK2 using a specific anti-NTRK2-243aa antibody (Fig. 4B and Supplementary Fig. 4C). Further, we detected NTRK2-243aa sequence by mass spectrometry (Supplementary Fig. 4D, Supplementary File 2). We found NTRK2-243aa was significantly decreased in glioma tissues and was negatively correlated with pathological grades (Supplementary Fig. 4E, F). The expression of NTRK2-243aa in GBM cells was markedly reduced (Fig. 4C). In addition, NTRK2-243aa overexpression exerted an inhibitory regulation on glucose uptake, lactate production, ECAR, and cell proliferation (Fig. 4D–G), and reduced HK2 and PKM2 proteins expression (Supplementary Fig. 4I). Interestingly, NTRK2-243aa overexpression could fully reverse the promoting effect on glucose uptake, lactate production, but only partly restore the facilitating effect on proliferation exerted by circNTRK2 knockdown (Supplementary Fig. 4J–L).

### PAX5 activated the transcription of HK2 and PKM2, impeding glycolysis and proliferation in GBM cells

We used TCGA database to analyze genes related to *HK2* and *PKM*; the PKM2 protein is encoded by the *PKM* gene (Supplementary Files 3, 4). We identified 877 genes related to *HK2*, 231 genes related to *PKM* and 20 genes potentially correlated with both (Supplementary File 5 and Supplementary Fig. 5A). Next, we validated the mRNA and protein expression levels of related transcription factors among these 20 genes following NTRK2-243aa overexpression (Supplementary Fig. 5B, D). Interestingly, we found that PAX5 protein was significantly downregulated upon NTRK2-243aa overexpression. The correlation of the expression of *PAX5*, *HK2*, and *PKM* is shown in Supplementary Fig. 5C.

**Fig. 5:**
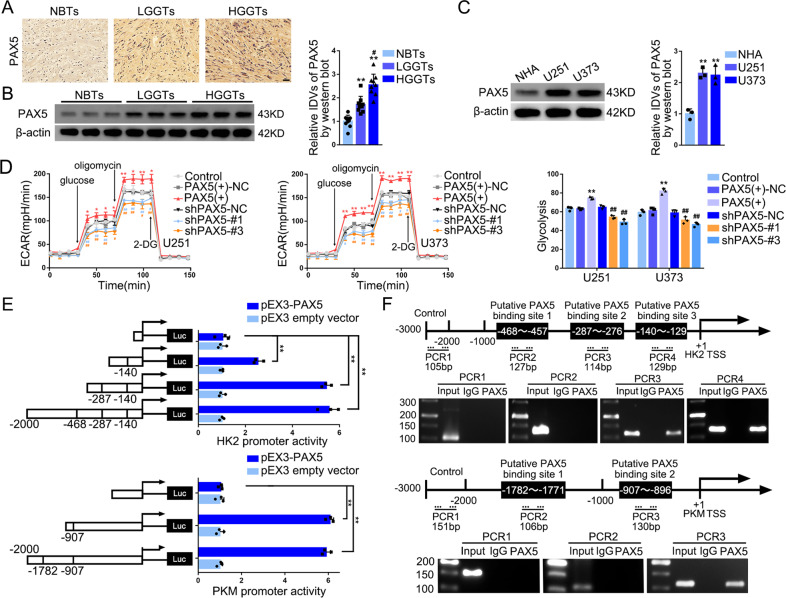
PAX5, upregulated in GBM Cells, facilitated Glycolysis and Proliferation by Transcriptionally promoting HK2 and PKM2 Expression. **A** IHC shows the expression and distribution of PAX5 in NBTs, LGGTs, and HGGTs (×200; scale bar represents 150 µm). **B** NBTs deriving from regions adjacent to the GBM and GBM tissues of different grades (LGGTs, HGGTs) were analyzed for PAX5 protein levels by western blotting. The IDVs of the bands were statistically analyzed. Data are manifested as mean ± SD (*n* = 9, each group). **P < 0.01, compared with NBTs group; ^#^*P* < 0.05 compared with LGGTs group. **C** Expression of PAX5 in NHA, U251, and U373 cells. The IDVs of the bands were statistically analyzed. Data are manifested as mean ± SD (*n* = 3, each group). **P < 0.01 compared to NHA group. **D** Effects of PAX5 overexpression or knockdown on ECAR in U251 and U373 cells were determined. Data are presented as mean ± SD (*n* = 3, each group). *P < 0.05, **P < 0.01 compared with PAX5(+)-NC group; ^#^*P* < 0.05, ^##^*P* < 0.01 compared with shPAX5-NC group. Glycolytic capacity and glycolytic reserve in each ECAR group were analyzed (Supplementary Fig. 5I, J). **E** Schematic depiction of different reporter vectors and relative luciferase activity of HK2 and PKM. Data are presented mean ± SD (*n* = 3, each group). **P < 0.01. **F** The binding sites of PAX5 on the promoter region of HK2 and PKM were determined by ChIP assays. Transcription start sites (TSSs) were designated as +1. Immunoprecipitated DNA was amplified by primers represented by dashed lines, taken normal rabbit IgG as negative control and −2000 to −3000 as blank control.

We found the AUC of *PAX5* mRNA ROC curve was 0.692 (Supplementary Fig. 5E). We also discovered the WHO grade, IDH state, and 1p/19q codeletion were correlated with the expression of PAX5 (Supplementary Table 2). In addition, PAX5 protein level was prominently elevated in GBM tissues and positively correlated with pathological grades (Fig. 5A, B). The expression of PAX5 was higher in GBM cells (Fig. 5C). Likewise, glucose uptake, lactate production, ECAR, and cell proliferation were significantly upregulated in the PAX5(+) group. (Fig. 5D and Supplementary Fig. 5F–H). 2-DG could fully reverse the proliferation-promoting effect exerted by PAX5 overexpression (Supplementary Fig. 5K). The putative binding motif of PAX5 is GAGCGTGACC (Supplementary Fig. 5L). The fluorescence activity of pEX3-PAX5 was markedly improved in luciferase reporter assays (Fig. 5E). Further, using chromatin immunoprecipitation (ChIP) experiments we identified that PAX5 bound to the promoter regions of HK2 at −140 and −287, and PKM at −907, respectively (Fig. 5F). Moreover, we noticed PAX5 overexpression increased the mRNA and protein expression levels of HK2 and PKM2 (Supplementary Fig. 5M–O). HK2 or PKM2 knockdown reversed the PAX5 overexpression-mediated enhancement of glucose uptake, lactate production, and glycolysis (Supplementary Fig. 5P–R).

### Ectopic expression of NTRK2-243aa regulated glycolysis and proliferation in GBM cells through the phosphorylation and half-life reduction of PAX5

**Fig. 6:**
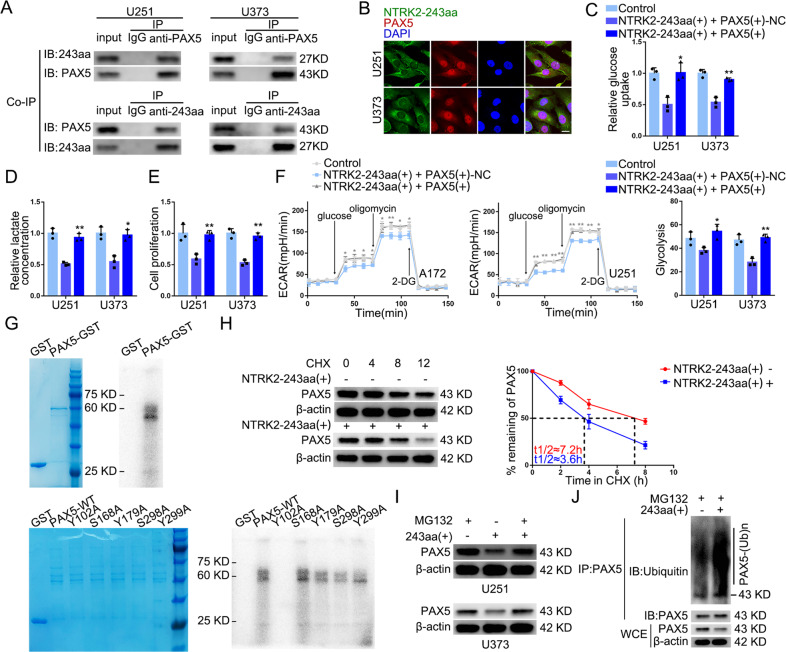
NTRK2-243aa attenuated the Half-life of PAX5 Protein via phosphorylating PAX5 to regulate Glycolysis and Proliferation of GBM Cells. **A** The interaction of NTRK2-243aa and PAX5 in the cytoplasmic proteins of U251 and U373 was determined by co-IP assays. **B** Colocalization of NTRK2-243aa and PAX5 in U251 and U373 cells was determined by IF staining and observed by laser scanning confocal microscopy (NTRK2-243aa was labeled in green by secondary antibody against anti-NTRK2-243aa antibody. PAX5 antibody was labeled in red by secondary antibody against anti-PAX5 antibody and nuclei were labeled in blue by DAPI. Scale bar represents 10 µm). **C**–**E** The reverse effects of PAX5 on NTRK2-243aa in terms of glucose uptake, lactate production, and proliferation in U251 and U373 cells. Data are presented as mean ± SD (*n* = 3, each group). *P < 0.05, **P < 0.01, compared with NTRK2-243aa(+) + PAX5(+)-NC group. **F** The reverse effects of PAX5 on ECAR in U251 and U373 cells were tested and glycolysis levels in each ECAR group were analyzed. Data are presented as mean ± SD (*n* = 3, each group). *P < 0.05, **P < 0.01 compared with NTRK2-243aa(+) + PAX5(+)-NC group. Glycolytic capacity and glycolytic reserve in each ECAR group were analyzed (see Supplementary Fig. 6G, H). **G** The fusion protein PAX5-GST and NTRK2-243aa-GST were constructed, and phosphorylation of PAX5 by NTRK2-243aa was detected by in vitro kinase assays. The upper left image shows the fusion protein added in in vitro kinase system. The upper right image shows the phosphorylatable state of PAX5 by autoradiography. Lower image shows the phosphorylatable state of PAX5-WT and PAX5 with the sited mutations on Y102, S168, Y179, S298, and Y299. **H** Half-life of PAX5 protein was measured in HEK-293T cells upon overexpression of NTRK2-243aa and wild type PAX5 after treatment with CHX. IDVs of the bands were statistically analyzed. Data are presented as mean ± SD (*n* = 3, each group). **I** Expression levels of PAX5 in U251 and U373 cells with NTRK2-243aa overexpression after treatment with 20 μM MG132 for 8 h. IDVs of the bands were statistically analyzed. Data are presented as mean ± SD (*n* = 3, each group). **J** PAX5 ubiquitination level with upregulating NTRK2-243aa was measured after treatment with 20 μM MG132 for 8 h. WCE represents the whole cell extract.

PPA-Pred2 (https://www.iitm.ac.in/bioinfo/PPA_Pred/) predicted that NTRK2-243aa might interact with PAX5 (Supplementary Fig. 6A). Coimmunoprecipitation (co-IP) analyses using cytoplasmic extracts confirmed the interaction between NTRK2-243aa and PAX5 (Fig. 6A). IF assays showed that NTRK2-243aa was mainly located in the cell cytoplasm, whereas PAX5 was distributed in the cytoplasm as well as the nucleus (Fig. 6B). PAX5 overexpression restored the inhibitory effects caused by NTRK2-243aa overexpression (Fig. 6C–F). Interestingly, the mRNA levels of PAX5 remained unchanged, whereas its protein levels were reduced following NTRK2-243aa overexpression (Supplementary Fig. 6B, C), indicating that NTRK2-243aa might interact with PAX5 post-translationally. Using the SMART database (http://smart.embl-heidelberg.de/), we found the TyrKC (tyrosine kinase, catalytic domain) and S_TKC (serine/threonine protein kinase, catalytic domain) domains in NTRK2-243aa (Supplementary Fig. 6D). Meanwhile, scanning the PhosphositePlus (https://www.phosphosite.org/homeAction) revealed the presence of multiple potential phosphorylation sites in PAX5 (Supplementary Fig. 6E). Based on these findings, we speculated that NTRK2-243aa phosphorylates PAX5 protein. The proteomic mass spectrometry-predicted phosphorylation sites of PAX5 are shown in Supplementary Fig. 6F. Subsequently, we mutated five putative phosphorylation sites—Y102, S168, Y179, S298, and Y299—to A (Y, S, and A are short for tyrosine, serine, and alanine, respectively) simulating the unphosphorylatable state. The in vitro kinase assay revealed that NTRK2-243aa can phosphorylate the Y102 site of PAX5 (Fig. 6G).

We observed the overexpression of NTRK2-243aa attenuated the half-life of wild-type PAX5 protein (Fig. 6H), whereas posed no effect to the Y102A-mutant PAX5 protein (Supplementary Fig. 6I). IF assay showed PAX5 was mainly located in the nucleus of HEK-293T cells (Supplementary Fig. 6J). Furthermore, the proteasome inhibitor MG132 reversed the decline of PAX5 induced by NTRK2-243aa overexpression (Fig. 6I), suggesting that PAX5 is degraded through the ubiquitin-proteasome pathway. The ubiquitination levels of PAX5 were increased upon NTRK2-243aa overexpression (Fig. 6J). Next, we predicted the E3 ligases that might participate in PAX5 degradation using UbiBrowser (http://ubibrowser.ncpsb.org.cn/ubibrowser_v3/) (Supplementary File 6). The expression level of PAX5 increased upon UHRF2 knockdown (Supplementary Fig. 6K). In addition, the ubiquitination level of PAX5 and expression of p-PAX5 increased in NTRK2-243aa (+) group but decreased sharply when Y102 of PAX5 was mutated. In the presence of NTRK2-243aa and Y102 of PAX5, overexpression of UHRF2 promoted PAX5 ubiquitination whereas attenuated PAX5 protein expression (Supplementary Fig. 6L). Therefore, we concluded that NTRK2-243aa phosphorylates PAX5 on Y102 and promotes its degradation via UHRF2 through the ubiquitin-proteasome pathway.

### Overexpression of MBNL1 and circNTRK2 combined with PAX5 knockdown exerted the optimum tumor-suppressive effect and conferred the longest survival time in nude mice

**Fig. 7:**
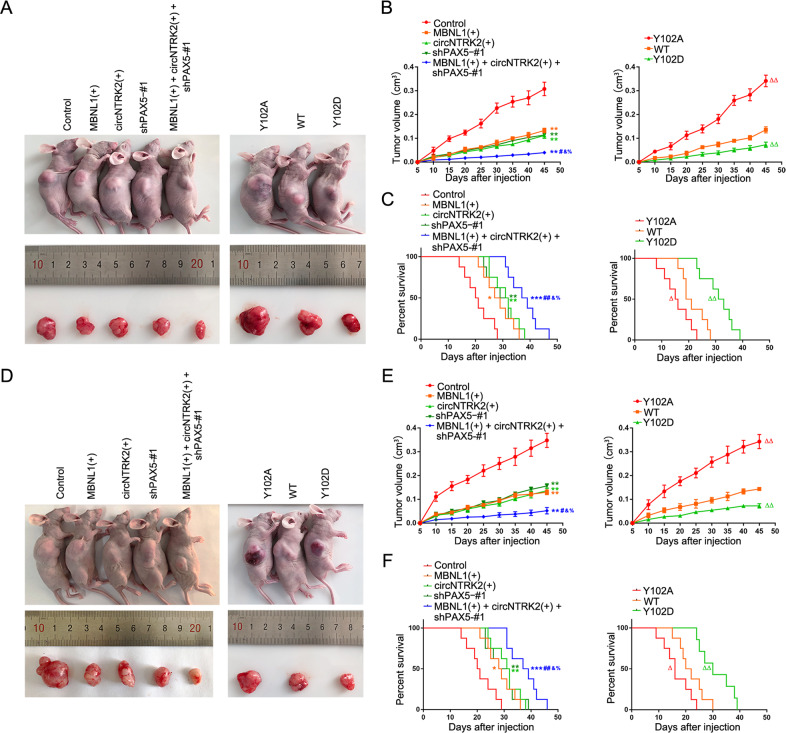
Studies of tumor xenograft. **A** The nude mice carrying U251 cell suspension formed tumors in respective groups are shown. The sample tumors resected from respective groups are shown. **B** Tumor growth curves are shown. Tumor volume was calculated every 5 days after U251 cell suspension injection, and the tumor was resected at 45 days. Data are presented as the mean ± SD (*n* = 8, each group). **P < 0.01 compared with Control group; ^#^*P* < 0.05 compared with MBNL1(+) group; ^&^*P* < 0.05 compared with circNTRK2(+) group; ^%^*P* < 0.05 compared with shPAX5-#1 group; ^ΔΔ^*P* < 0.01 compared with WT group. **C** Survival curves of nude mice U251 cell suspension injected into the right striatum are shown (*n* = 8, each group). *P < 0.05, **P < 0.01, ^***^P < 0.001 compared with Control group; ^##^*P* < 0.01 compared with MBNL1(+) group; ^&^*P* < 0.05 compared with circNTRK2(+) group; ^%^*P* < 0.05 compared with shPAX5-#1 group; ^Δ^*P* < 0.05, ^ΔΔ^*P* < 0.01 compared with WT group. **D** The nude mice carrying U373 cell suspension formed tumors in respective groups are shown. The sample tumors resected from respective groups are shown. **E** Tumor growth curves are shown. Tumor volume was calculated every 5 days after U373 cell suspension injection, and the tumor was resected at 45 days. Data are presented as the mean ± SD (*n* = 8, each group). **P < 0.01 compared with Control group; ^#^*P* < 0.05 compared with MBNL1(+) group; ^&^*P* < 0.05 compared with circNTRK2(+) group; ^%^*P* < 0.05 compared with shPAX5-#1 group; ^ΔΔ^*P* < 0.01 compared with WT group. **F** Survival curves of nude mice U373 cell suspension injected into the right striatum are shown (*n* = 8, each group). *P < 0.05, **P < 0.01, ^***^P < 0.001 compared with Control group; ^##^*P* < 0.01 compared with MBNL1(+) group; ^&^*P* < 0.05 compared with circNTRK2(+) group; ^%^*P* < 0.05 compared with shPAX5-#1 group; ^Δ^*P* < 0.05, ^ΔΔ^*P* < 0.01 compared with WT group.

To detect the effect of MBNL1, circNTRK2, PAX5, as well as that of PAX5 phosphorylation on xenografts we divided nude mice into five and three groups, respectively. We observed, in the MBNL1(+) + circNTRK2(+) + shPAX5-#1 and Y102D (tyrosine at 102 mutated to aspartic acid to simulate the phosphorylatable state) groups, the volumes of subcutaneous xenografts were prominently smaller and orthotopic nude mice had longer survival time. Meanwhile, in the MBNL1(+) + circNTRK2(+) + shPAX5-#1 and Y102D groups, the volumes of subcutaneous xenografts were the smallest and orthotopic nude mice had the longest survival time (Fig. 7A–F). Further, the levels of HK2 and PKM2 in the subcutaneous xenografts of MBNL1(+), circNTRK2(+), and shPAX5-#1 groups were decreased and particularly MBNL1(+) + circNTRK2(+) + shPAX5-#1 and Y102D groups remained the lowest (Supplementary Fig. 7A, B). Also, we obtained pathological sections of subcutaneous and orthotopic xenografts for further analyses (Supplementary Fig. 7C, D). Finally, immunohistochemistry (IHC) data of MBNL1, NTRK2-243aa, and PAX5 staining in the tissues of patients with GBM are shown in Supplementary Fig. 7E.

### Mechanism of the MBNL1/circNTRK2/PAX5 pathway to regulate HK2 and PKM2 in GBM cells

**Fig. 8:**
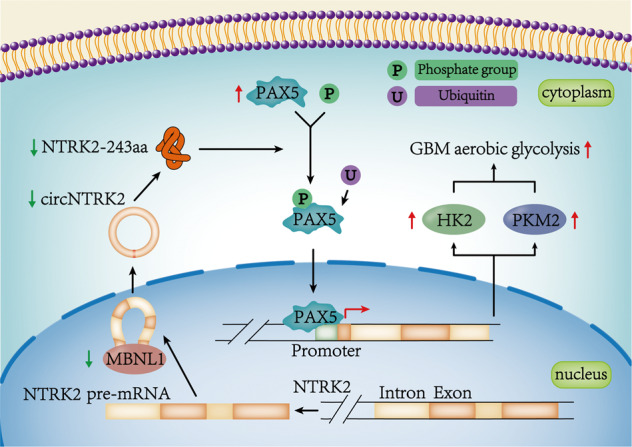
Schematic diagram of MBNL1/circNTRK2/PAX5 pathway to regulate glycolysis and proliferation in GBM cells.

Collectively, our results demonstrate that the downregulation of MBNL1 plays an essential role in the development and progression of GBM through the inhibition of glycolysis and proliferation by circNTRK2-dependent regulation of the PAX5 expression via encoding NTRK2-243aa (Fig. 8).

## Discussion

There is accumulating evidence to support aerobic glycolysis as the most important energy metabolism characteristic of malignant tumors, promoting their occurrence and development [28]. The proteins involved in the regulation of the glucose metabolism pathway include members of the GLUT, HK, PFK, PK, and LDH families. Increasing studies have reported that knocking-down or decreasing the activity of these proteins inhibits glycolysis and the malignant behavior of glioma, liver, colorectal, breast, and lung cancers [29–31]. Therefore, enhanced aerobic glycolysis, one of the main hallmarks of cancer, is considered a potential target for chemotherapy [32].

There is mounting evidence that RBPs are involved in regulating aerobic glycolysis in tumors. In particular, R-2HG inhibits the FTO/RBP-YTHDF2-mediated post-transcriptional upregulation of the expression of PFKP and LDHB, thereby suppressing aerobic glycolysis [33]. In addition, MBNL1 inhibits the migration and invasion of prostate cancer cells, exerting tumor-suppressive effects [34]. Recently, Voss et al. reported that under hypoxic condition, MBNL1 was exported out of the nucleus, resulting in reduction of MBNL1 activity and promoting stem-like phenotypes and tumor growth [35]. In the present study, we detected a significant decrease in the expression of MBNL1 in GBM. Moreover, we found that the overexpression of MBNL1 significantly inhibited glycolysis and proliferation. Previous studies have indicated that circRNAs play multiple roles in tumors. For instance, circ_0020710 facilitates the migration, invasion, and immune escape of melanoma cells [36]. The autophagy-related circCDYL promotes the malignant progression of breast cancer [37]. We discovered that circNTRK2 was downregulated in GBM. Moreover, the overexpression of circNTRK2 significantly inhibited glycolysis and proliferation. Collectively, our results indicate that MBNL1 and circNTRK2 inhibit the glycolysis and proliferation of GBM cells, thereby functioning as tumor suppressors.

Many RBPs are known to be involved in regulating the synthesis and degradation of circRNAs [38]. For example, RBP-RBM3 enhances the formation of SCD-circRNA 2, promoting cell proliferation in hepatocellular carcinoma [39]. In our study, circRNA microarray and qRT-PCR verified that hsa_circ_0139157 (circNTRK2) is significantly increased in GBM cells transfected with MBNL1(+) vector. Nascent RIP and RNA pull-down assays validated that MBNL1 binds to the *NTRK2* pre-mRNA. We also found that flanking introns of *NTRK2* pre-mRNA are necessary for MBNL1 to facilitate circNTRK2 biosynthesis. Besides, silencing circNTRK2 reversed the inhibitory effect exerted by MBNL1 overexpression. Together, our results reveal that MBNL1 regulates the reprogramming of glucose metabolism through the promotion of circNTRK2 biosynthesis. Similar to our findings, MBNL1 promotes circMBL biosynthesis by binding to specific sites in the intron of the *MBNL1* pre-mRNA [40]. Likewise, HNRNPL regulates the formation of multiple circular RNAs via binding to their pre-mRNAs and directly regulating their alternative splicing [41]. Besides, Xu et al., reported that circSMARCA5 is recruited to its parent gene locus, leading to R-loop formation and decrease in the expression of SMARCA5 [42]. Therefore, the mechanism underlying the interaction between the circNTRK2 and the parent gene NTRK2 remain to be studied further.

Several studies have reported that circRNAs might encode polypeptides. For instance, PPP1R12a-73aa, encoded by circPPP1R12a, promotes the proliferation and metastasis in colon cancer [43]. Importantly, IRES and ORF are the two important components of circ-SHPRH, encoding SHPRH-146aa, inhibits the proliferation and tumorigenesis in glioma [44]. In this study, luciferase reporter assays verify the activity of NTRK2-IRES. In addition, a series of plasmids and specific antibodies were generated to confirm the encoding ability of NTRK2-ORF. We observed that NTRK2-243aa was downregulated in GBM and negatively correlated with glioma grades. The overexpression of NTRK2-243aa significantly inhibited cell glycolysis and proliferation. Besides, the reason for the failure of 2-DG and NTRK2-243aa in fully reversing the promoting effect of the circNTRK2 knockdown on cell proliferation has been under investigation by our group and will be addressed in future studies.

We analyzed genes related to *HK2* and *PKM*, and conducted researches on PAX5. Notably, PAX5 facilitates the proliferation of small-cell lung cancer cells by binding to the c-Met promoter sequence [45]. Besides, PAX5 is an important oncogene in neuroblastoma and a tumor suppressor in acute lymphoblastic leukemia [46, 47]. Our results confirmed that PAX5 was highly expressed in GBM. Knockdown of PAX5 significantly inhibited glycolysis and proliferation, indicating that PAX5 promoted malignant progression in GBM.

In the present study, we verified the interaction of NTRK2-243aa and PAX5 using co-IP and IF assays. Notably, PAX5 is phosphorylated at T264 and T299, reducing the transcriptional inhibition of BLIMP1, thus promoting the differentiation of B-cells into plasma cells [48]. Our study first clarified that phosphorylation on Y102 of PAX5 by NTRK2-243aa is important for its ubiquitination which UHRF2 participates in. However, the way in which UHRF2, as a E3 ligase, interact with PAX5 needs to be further studied. Similarly, Yang et al. [49] reported that the EGF-AKT1-mediated binding of FBXW2 to the S552 phosphorylation site of β-catenin promotes the ubiquitination and degradation of β-catenin through the proteasomal pathway, and thereby inhibits the migration and invasion of lung cancer cells. Likewise, Li et al. [50] reported that dioscin attenuates the phosphorylation of Skp2 on S72, eventually enhancing the Cdh1-mediated lysine 48-linked polyubiquitination and degradation of Skp2.

Several studies have revealed that PAX5 mainly functions as a transcriptional activator in various cancers. In prostate cancer, PAX5 promotes the expression of IDH1-AS1 transcriptionally, regulating cell proliferation and apoptosis [51]. Moreover, PAX5 promotes the expression of MCOLN2 by binding to the MCOLN2 promoter region [52]. In our study, ChIP and luciferase reporter gene assays indicated PAX5 transcriptionally promotes the expression of HK2 and PKM2, thus promoting glycolysis and proliferation of GBM cells, playing a role as a pro-oncogenic factor.

Finally, U251 and U373 cells were applied in GBM transplantation in nude mice, which demonstrated that overexpression of MBNL1 and circNTRK2 combined with PAX5 knockdown effectively inhibits the formation of GBM cell xenografts, as well as significantly prolongs the survival of orthotopic nude mice. Therefore, MBNL1, circNTRK2, and PAX5 might be effective targets in GBM treatment, possessing potential clinical value, but the survival analysis and targeted drugs of these marker in GBM remain to be elucidated.

In conclusion, we found that MBNL1 promotes the expression of circNTRK2 by binding to the *NTRK2* pre-mRNA. NTRK2-243aa, encoded by circNTRK2, phosphorylates PAX5 on Y102 facilitating the degradation of PAX5. PAX5 promotes the expression of HK2 and PKM2 at transcriptional level. Our results demonstrate the role and mechanism of the MBNL1/circNTRK2/PAX5 pathway in regulating glycolysis in GBM and provide new molecular targets for therapy of GBM.

## Materials and methods

### Analysis of the TCGA and genotype-tissue expression project (GTEx) databases

The expression, prognosis, clinical outcome, and correlational data of patients with glioma were obtained from the TCGA database (https://portal.gdc.cancer.gov/), whereas normal control data were obtained from the GTEx database (https://www.gtexportal.org/home/). All analyses were performed in R (University of Auckland, New Zealand).

### Human brain tissues and GBM specimens

Human GBM and normal brain tissues were collected from patients in the Department of Neurosurgery, Shengjing Hospital, China Medical University. All surgically resected tissue samples were divided into two parts, one part was immediately frozen in liquid nitrogen overnight and then stored at −80 °C, the other was fixed with 4% paraformaldehyde for 36 h, then embedded in paraffin to prepare sections. All patients signed informed consent, and our study was approved by the Ethics Committee of the Shengjing Hospital Affiliated to China Medical University. GBM tissues were divided into two groups according to the 2007 classification system of tumors: low-grade glioma tissues (LGGTs), which are WHO grade I and II (*n* = 9), and high-grade glioma tissues (HGGTs), which are WHO grade III and IV (*n* = 9).

### Cell lines and cell culture

Normal human astrocytes (ZY-1028) were purchased from ScienCell Research Laboratories (CA, USA) and cultured in astrocyte medium (ScienCell, CA, USA) supplemented with 10% fetal bovine serum (ScienCell, CA, USA), Astrocyte Growth Supplement (ScienCell, CA, USA) and 1% penicillin-streptomycin solution (ScienCell, CA, USA). Human GBM cell lines (A172, T98G, U251, U373, and LN229) and human embryonic kidney 293T (HEK-293T) cells were purchased from the Shanghai Institutes for Biological Sciences Cell Resource Center and cultured in Dulbecco’s modified Eagle’s medium (HyClone, UT, USA) supplemented with 10% fetal bovine serum (Gibco, NY, USA). All cells were authenticated by Short Tandem Repeat (STR) with Cell Line Authentications and maintained in an incubator at 37 °C and 5% CO_2_.

### qRT-PCR (quantitative real-time PCR) and RNase R treatment

RNA from cells and tissues was extracted using the Trizol reagent (Life Technologies Corporation, CA, USA) according to the manufacturer’s instructions. The concentration and quality of extracted RNA were measured using a Nanodrop Spectrophotometer (ND-100, Thermo Fisher Scientific, MA, USA). The one-step SYBR Prime-Script RT-PCR kit (Takara, Kyoto, Japan) and 7500 FAST system (ABI, Shanghai, China) were used to detect the expression levels of the genes of interest, with β-actin used as an internal reference. Referring to the RNase R instructions, 5 μg total RNA, 2 μL 10× reaction buffer and 20 U RNase R (20 U/μL, Thermo Fisher Scientific, MA, USA) were supplemented with water to make a 20 μL system. Next, the system was incubated at 37 °C for 40 min then 70 °C for 10 min. Then qRT-PCR was performed to detect the expression level of circNTRK2. In our study, the primers used are shown in Supplementary Table 3.

### IHC

IHC was performed following the instructions of Ultrasensitive SP kit (Fuzhou MaiXin Biotech, Fujian, China). Briefly, Human glioma tissue samples and xenograft tumors were fixed with 4% paraformaldehyde for 36 h; then, they were embedded in paraffin and sectioned at a thickness of 5 µm. These sections were deparaffinized in xylene, hydrated in graded ethanol, and boiled in EDTA antigen-unmasking solution. When cooled to room temperature, the slides were incubated in peroxide at room temperature for blocking endogenous peroxidase, blocked with goat serum, and stained with the corresponding primary monoclonal antibodies overnight at 4 °C. After washing with PBS thrice and incubating with goat anti-rabbit/mouse secondary antibody at room temperature for 10 min, the slides were treated with a Dako REAL EnVision Detection System, Peroxidase/DAB+, Rabbit/Mouse DAB kit (Fuzhou MaiXin Biotech, Fujian, China). Lastly, the slides were viewed under a light microscope.

### Western blot analysis

Briefly, the collected cells were lysed on ice with RIPA (Beyotime Institute of Biotechnology, Jiangsu, China) buffer for 30 min, and then centrifuged at 17,000 × *g* at 4 °C for 45 min. Protein concentration was measured using BCA protein kit (Beyotime Institute of Biotechnology, Jiangsu, China). After SDS-PAGE electrophoresis, proteins were transferred to PVDF membranes (Millipore, MA, USA), which were sealed in 5% BSA at room temperature for 2 h, and then incubated in primary antibody at 4 °C overnight. The next day, membranes were washed three times with 0.1% TTBS, and incubated at room temperature with secondary antibody (Proteintech, Wuhan, China) cross-linked with horseradish peroxidase for 2 h, and then illuminated with ECL Bioluminescence Kit (Santa Cruz Biotechnology, TX, USA) using ChemImager 5500 V2.03 software according to instructions. The integrated density values (IDVs) were obtained and β-actin was used as internal reference. Fluor Chen 2.0 software was used to calculate the protein relative expression. The primary antibodies are shown in Supplementary Table 4. Full length uncropped original western blots used in the manuscript is shown as Supplementary Material File.

### Vectors construction and cell transfection

The vectors with the full-length sequence of MBNL1 and PAX5 (MBNL1(+) and PAX5(+)), as well as their respective empty vectors (MBNL1(+)-NC and PAX5(+)-NC), were constructed by GeneChem (Shanghai, China) using CMV-MCS-IRES-EGFP-SV40-neomycin and CMV-MCS-mCherry-SV40-puromycin vectors, respectively. The vector with the full-length sequence of circNTRK2 (circNTRK2(+)) and its respective empty vector (circNTRK2(+)-NC) were constructed by GenScript (Piscataway, NJ, USA) using pcDNA3.1 vectors. The short hairpin RNAs against MBNL1 (shMBNL1) and PAX5 (shPAX5) and their corresponding nonspecific (NC) vectors (shMBNL1-NC and shPAX5-NC) were introduced in pGPU6-GFP-Neo and pGPU6-mCherry-Puro vectors by GenePharma (Shanghai, China). For details and targeted sites, see Supplementary Table 5. When cell confluence of the 24-well plate (Corning, NJ, USA) reached 70–80%, the transfection reagent Lipofectamine 3000 (Life Technologies, CA, USA) was used to transfect above vectors into U251 and U373 cells according to the manufacturer’s protocols. Then G418 and puromycin (Sigma-Aldrich, MO, USA) were used to construct stably-transfected cell lines within approximately 4 weeks (The plasmid with the highest knockdown efficiency was selected by qRT-PCR for stable transfection after transient transfection was carried out for 48 h using Lipofectamine 3000 according to instructions.), and the transfection efficiency was detected by qRT-PCR as well as western blotting (Supplementary Fig. 8).

### IF assay

When the cell confluence of the glass culture dishes (201200, Sorfa, Zhejiang, China) reached 60–70%, the cells were fixed with 4% paraformaldehyde at room temperature for 20 min, permeated with 0.2% Triton X-100 (Sorfa, Zhejiang, China) for 10 min, and then blocked with 5% BSA (Sorfa, Zhejiang, China) at room temperature for 2 h. The cells were then incubated with antibodies at 4 °C overnight. The next day, these cells were washed using 0.1% PBST thrice, and then incubated with Alexa-Fluor-488-labeled Goat anti-Mouse IgG (H + L) or anti-Rabbit IgG (H + L) and Alexa-Fluor-555-labeled Donkey anti-Mouse IgG (H + L) or anti-Rabbit IgG (H + L) (Beyotime Institute of Biotechnology, Jiangsu, China) at room temperature for 2 h. Subsequently, the cells were washed with 0.1% PBST thrice. The nuclei were stained with 0.5 mg/mL DAPI at room temperature for 5 min, and the cells were observed under a laser scanning confocal microscope (LSCM) (confocal microscope parameters: Gain value, 2; Gamma value, 1; DAPI Laser Strength, 79%; Alexa Fluor, 68%). The antibodies used are provided in Supplementary Table 4.

### Glucose uptake assay

After cultured in sugar-free DMEM for 10 h, cells were incubated with high-glucose DMEM mixed with 2-DG (30 mmol/L, Sigma-Aldrich, MO, USA) for 30 min. Intracellular glucose levels were detected by glucose uptake test (ab136955, Abcam, USA) according to the instructions. Briefly, cells were lysed using lysis buffer, then heated to 85 °C for 40 min and cooled on ice for 5 min. Supernatants were transferred to 96-wells plates (Corning, NJ, USA), added with reaction mix A and incubated for 1 h at 37 °C. Next, extraction buffer was added and heated to 90 °C for 40 min, cooled on ice for 5 min and then reaction mix B was added and analyzed the plate on microplate reader. Eventually, the results were normalized to the number of cells.

### Lactate assay

Cells inoculated in 96-well plates (Corning, NJ, USA) reached confluence 80–90% were cultured for 24 h. The supernatants collected were measured using a lactate colorimetric assay (Jiancheng, Nanjing, China) then read at 540 nm according to the manufacturer’s instructions. Results were normalized to the cell number.

### Cell proliferation assay

2000 cells seeded in 96-well (Corning, NJ, USA) were cultured in an incubator at 37 °C with 5% CO_2_ overnight. Next, 20 μL Cell Counting kit-8 (Beyotime Institute of Biotechnology, Jiangsu, China) was added in each well, and cells were cultured for 4 h then read at 450 nm according to the instructions. Cell number was rechecked after measurement. Finally, results were normalized to the cell number.

### ECAR measurement

ECAR was measured according to the protocol of the XF glycolysis stress test kit (103020-100, Seahorse Bioscience, MA, USA), using the XF24 Extracellular Flux Analyzer (Seahorse Bioscience, MA, USA), kindly provided by the Experimental Teaching Center, School of Public Health, China Medical University. In short, cells were plated 70,000 cells/well in XF-24 plate (100777-004, Seahorse Biosciences, MA, USA). Meanwhile, the XF-probe (100850-001, Seahorse Bioscience, MA, USA) was hydrated overnight. The next day, cells were cultured in XF medium (without glucose, adding 2 mM glutamine, pH = 7.4, Seahorse Bioscience, MA, USA) in a non-CO_2_ incubator at 37 °C to maintain glucose starvation conditions for 2 h. The ECAR was measured under glucose starvation conditions (0–30.42 min, representing as non-glycolysis state). Next, to determine the basal glycolysis rate, we added glucose in final concentration 10 mM and ECAR was measured thrice again (40.4–69.48 min). Oligomycin was subsequently injected to evaluate glycolysis capacity. Oligomycin inhibit oxidative phosphorylation by leading cells to completely depend on glycolysis for energy and resulting in increasing their acidic products, thereby further increasing ECAR (79.43–108.52 min). Lastly, 2-deoxyglucose (2-DG) was injected to inhibit total glycolysis competitively (118.45–147.53 min). Measurements were all normalized by cell number rechecked after experiments. Subtracting the non-glycolysis ECAR from the ECAR obtained after adding glucose makes glycolysis. Subtracting the non-glycolysis ECAR from ECAR obtained after adding oligomycin makes glycolytic capacity. Subtracting glycolysis from glycolytic capacity makes glycolytic reserve. Data were analyzed using Seahorse XF-96 WAVE software and presented as mpH/min.

### Protein stability measurement

Cells were cultured in the medium containing 10 µM cycloheximide (CHX, Sigma-Aldrich, MO, USA) to inhibit protein biosynthesis. Total protein was extracted at different time points, then detected using western blot analysis. The half-life of protein was determined by the reduction of its level to 50% at a certain time point compared with that at the beginning of the experiment (zero time).

### In vivo ubiquitination assay

PAX5 together with HA-Ub and NTRK2-243aa plasmids were transfected into HEK-293T and incubated with 10 μM MG132 for 4 h. Cell lysates were prepared using the RIPA buffer (Beyotime Institute of Biotechnology, Jiangsu, China) mixed with 10 mM N-ethylmaleimide and protease inhibitors (Beyotime Institute of Biotechnology, Jiangsu, China). After 30 s of ultrasonic treatment, the supernatant was boiled at 95 °C for 15 min, followed by dilution with RIPA buffer containing 0.1% SDS (Beyotime Institute of Biotechnology, Jiangsu, China), and then centrifuged at 12,000 × *g* for 15 min at 4 °C. The supernatant was incubated with anti-PAX5 antibody and 30 μL protein A-Sepharose beads (Sigma-Aldrich, MO, USA) overnight at 4 °C. After extensive washing and centrifugation, bound proteins were washed out by boiling with 2 × SDS sample loading buffer at 95 °C for 5 min, and PAX5 ubiquitination was determined by western blot analysis. The primary antibodies are shown in Supplementary Table 4.

### FISH assay

To confirm the position of circNTRK2 in NHA, U251, and U373 cells, we used the circNTRK2-probe (red-labeled, Biosense, Guangzhou, China). In brief, cells plated on the cell slides were first fixed with 4% paraformaldehyde for 10 min, and then washed with DEPC twice. Next, 0.2% Triton X-100 (Beyotime Institute of Biotechnology, Jiangsu, China) was added for 10 min at room temperature, then washed the plate 3 times with PBS. PCR-grade protease K (Roche Diagnostics, Mannheim, Germany) was added for 15 min at 37 °C. Further, a 20 μL prehybridization solutions in SSC was used to treat for 30 min at 37 °C. At the same time, the hybridization reaction solution was prepared as 1:40 and placed on ice for use according to the instructions. The hybridization reaction solution covered the sample, denaturated at 73 °C for 5 min, and then hybridized overnight at 37 °C. The next day the sample was washed with 25% formamide/2 × SSC preheated at 53 °C for 2 times, 5 min per time, and then washed with 0.1% NP-40/2 × SSC preheated at 42 °C for 2 times, 5 min per time. Next, anti-Digoxin Rhodamine coupling agent (1:100, Exon Biotch Inc., Guangzhou, China) was used to conjugate for 1 h at 37 °C in the dark, and wash the sample with PBS for 3 times, 5 min each time. DAPI (Beyotime Institute of Biotechnology, Jiangsu, China) dyed the nucleus of cells and then wash the sample with PBS thrice, 5 min per time. Finally, cells were observed by LSCM (confocal microscope parameters: Gain value, 2; gamma value, 1; DAPI laser strength, 79%; Alexa Fluor, 68%). The probe for circNTRK2 is shown in Supplementary Table S3.

### Construction of reporter vector and luciferase reporter assay

The IRES sequence of circNTRK2 predicted by the database, its truncated sequence, and mutated sequence were cloned between the Renilla luciferase reporter gene and Firefly luciferase reporter gene. The inserted sequence and reporter gene had independent promoters and terminators respectively. Human full-length PAX5 gene was constructed into pEX3 (pGCMV/MCS/neo) vector (GenePharma, Shanghai, China). pGL3 vector with wild-type promoter or mutant promoter sequences of HK2 and PKM2 respectively were cotransfected with pEX3-PAX5 vector (or empty pEX3 vector as negative control) into HEK-293T cells using Lipofectamine 3000 (Life Technologies, CA, USA). Luciferase activity was measured using the Dual-Luciferase Reporter Assay kit (Promega, WI, USA) following the instructions, and relative luciferase activity was normalized by Renilla luciferase activity.

### Microarray analysis of human circRNA expression profile

The microarray analysis of the human circRNA expression profile was performed by using the Human circular RNA Array V2.0, designed by the Arraystar company (Aksomics, Shanghai, China), with the ability to detect 13, 617 human circular RNAs. In short, total RNA, isolated from U251 and U373 cells upon stable transfection with MBNL1(+)-NC and MBNL1(+), was reversely transcribed to cDNA. Next, cRNA was Cy3-labeled during its transcription from cDNA and then purified. Each slide of the circRNA array was hybridized with Cy3-labeled cRNA in hybridization wells. Then, slides were washed in staining dishes. The Arraystar microarray scanner was used to read the slide data. Last, differentially expressed circRNAs were obtained from the samples in the three groups after data analysis.

### Nascent RIP assay

The wild-type and mutant plasmids were constructed by Gene Pharma (Shanghai, China) and transfected into GBM cells. For nascent RIP, RNA pol II transcription was blocked with 100 μM DRB (D1916, Sigma-Aldrich, MO, USA) for 3 h and then restarted after removal of the DRB so as that newly transcribed RNA were labeled with 200 μM 4sU for 30 min (T4509, Sigma-Aldrich, MO, USA). The whole cell lysates were incubated with anti-human MBNL1 and normal anti-rabbit IgG respectively as RIP products, in which newly transcribed pre-mRNA-labeled by 4sU was purified and then verified by qRT-PCR following agarose gel electrophoresis (AGE). The primary antibodies are shown in Supplementary Table 4. The primers used for qRT-PCR are shown as Supplementary Table 3; the sequences of the wild-type and mutant vectors are listed in Supplementary Table 7.

### RNA pull-down assay

The interaction between *NTRK2* pre-mRNA and MBNL1 protein was detected using Pierce Magnetic RNA-Protein Pull-Down Kit (Thermo Fisher Scientific, MA, USA). In brief, biotin-labeled *NTRK2* pre-mRNA (Bio-NTRK2 pre-mRNA) was labeled in vitro with Biotin RNA-Labeling of Mix (Roche, Mannheim, Germany) transcription with T7 RNA Polymerase (Promega, WI, USA) and was purified using RNeasy Mini Kit (QIAGEN, Dusseldorf, Germany). Bio-*NTRK2* pre-mRNA and bio-antisense RNA were incubated in GBM cell lysate for 24 h at 4 °C, and then washed and mixed with magnetic beads (Thermo Fisher Scientific, MA, USA) to form probe-magnetic beads complex to pull down the protein that interacted with *NTRK2* pre-mRNA. Next, the pulled down proteins were identified by western blotting using β-actin as internal reference.

### In vitro kinase assay

Recombinant GST-tagged wild type, sited-mutation PAX5, and GST-tagged NTRK2-243aa were expressed in *E. coli* and purified on glutathione-Sepharose beads (Beyotime Institute of Biotechnology, Jiangsu, China). Recombinant proteins were incubated in kinase buffer (20 mM Tris–HCl, pH 7.4, 20 mM NaCl, 10 mM MgCl_2_, and 1 mM DTT) supplemented with 50 mM ATP (Beyotime Institute of Biotechnology, Jiangsu, China) and 5 μCi [γ-^32^P] ATP (PerkinElmer, MA, USA) for 20 min at 30 °C in the presence of 500 ng active NTRK2-243aa. The reaction products were analyzed by SDS-PAGE electrophoresis, transferred to PVDF membranes (Millipore, MA, USA), and autoradiographed. Finally, PVDF membranes were stained with Coomassie Brilliant Blue Fast Staining solution (Solarbio, Beijing, China).

### Co-IP assay

The interaction between NTRK2-243aa and the PAX5 proteins was detected by co-IP. Briefly, harvested cells were suspended in RIPA (Beyotime Institute of Biotechnology, Jiangsu, China) buffer on ice for 40 min and incubated with the antibody-coupled beads overnight at 4 °C. The beads were washed using high-salt buffer thrice. Finally, the beads were boiled in SDS buffer for 15 min, followed by western blotting.

### ChIP assay

ChIP was performed by using the enzymatic chromatin immunoprecipitation kit (Cell Signaling Technology, MA, USA). Briefly, the cells were subjected to cross-linking in 1% formaldehyde at 37 °C for 10 min, and then the cross-linking was stopped by glycine at 37 °C for 5 min. Next, the cells were collected within lysate buffer; these mixtures were divided into three parts: input control, incubated with normal rabbit IgG antibody, and incubated with anti-human PAX5 antibody under vertical rotation at 4 °C. The cross-linked DNA was delinked under a high-salt environment and treatment with protease K; the purified DNA was amplified by PCR, followed by AGE to detect the binding sites. The primers used for ChIP are shown in Supplementary Table 6.

### Xenograft mouse model in vivo

Four-week-old athymic nude mice (BALB/c) were purchased from the Cancer Institute of the China Academy of Medical Science (Beijing, China). The nude mice were divided into the following groups: control, NC (nonspecific control), MBNL1(+), circNTRK2(+), shPAX5, MBNL1(+) + circNTRK2(+) + shPAX5-#1, Y102A, WT, and Y102D. All experiments were carried out strictly following the Animal Welfare Act and approved by the Ethics Committee of China Medical University. Each nude mouse was subcutaneously injected with 200 μL of the cell suspension (containing 6 × 10^5^ cells) in the right flank region to construct the tumor transplantation model in vivo. The tumor formation time of each group was observed and recorded, and tumor formation rates were also calculated. The longest and shortest diameters of the transplanted tumor were measured every 5 days until 45 days, and the tumor volume was calculated according to the formula: *V* = (*L* × *W*^2^)/2 (*V* = tumor volume, *L* = the longest diameter of tumor, and *W* = the shortest diameter of tumor). The growth curves of the transplanted tumor were plotted. For survival analysis, 3 × 10^5^ glioma cells were implanted into the right striatum of nude mice using digital stereotaxic apparatus. In a span of 45 days, the survival of the nude mice was recorded, and Kaplan–Meier survival curves were constructed to analyze their survival rate. Meanwhile, body weight of nude mice was measured and plotted. The nude mice were sacrificed at day 45; the tumors were dissected and separated, the size of which were gauged. The expression levels of HK2 and PKM2 were detected taken β-actin as internal reference.

### Statistical analysis

All data were analyzed using GraphPad Prism v7.01 (GraphPad, CA, USA) manifested as mean ± standard deviation (SD), and statistically analyzed using one-way ANOVA. Differences between groups were regarded as significant when *P* < 0.05. Biological replications are shown in figure legends.

## Supplementary information

Supplementary Figures and Tables

Supplementary file 1

Supplementary file 2

Supplementary file 3

Supplementary file 4

Supplementary file 5

Supplementary file 6

supplementary file of western blot

cdd aj-checklist

## Data Availability

Data are available on reasonable request.
