# Supplementary material for: The MBNL1/circNTRK2/PAX5 pathway regulates aerobic glycolysis in glioblastoma cells by encoding a novel protein NTRK2-243aa
Source: Cell Death Dis. 2022 Sep 5;13(9):767. doi: 10.1038/s41419-022-05219-4 (PMC9445070; doi:10.1038/s41419-022-05219-4)
Supplement: Supplementary file 1 — Supplementary Figures and Tables [file 41419_2022_5219_MOESM1_ESM.docx]

**Supplementary Figure**

**Supplementary figure 1**

**
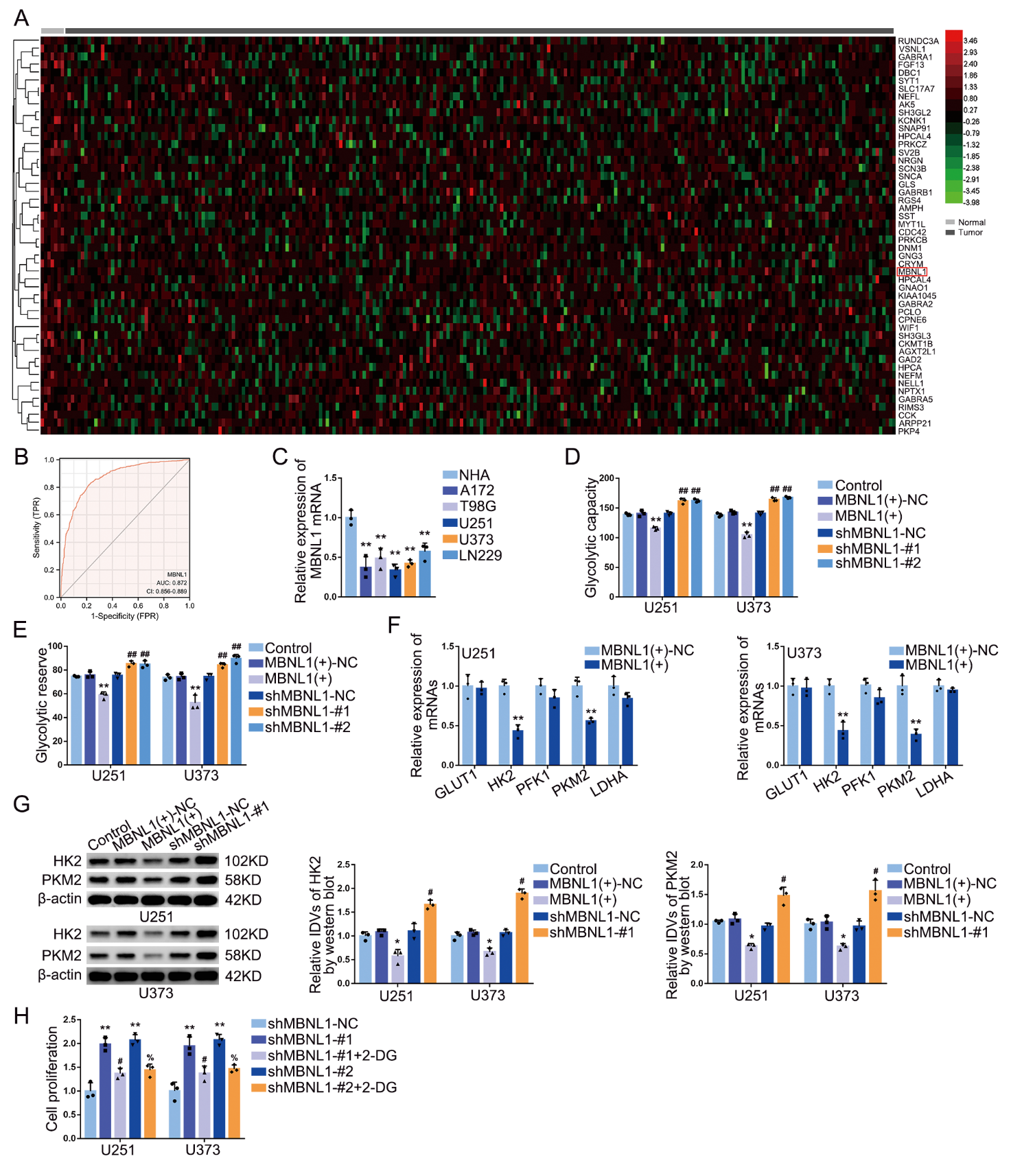
**

**Supplementary figure 1. Top 50 suppressors of GBM and inhibited effect of MBNL1 overexpression on glycolysis and proliferation of GBM cells.**

(A) Top 50 factors show low expression in GBM compared with normal brain tissues according to analysis of TCGA data. (B) ROC curve of *MBNL1* mRNA expression in glioma cohort of TCGA database. (C) mRNA expression levels of *MBNL1* in five GBM cell lines (A172, T98G, U251, U373, LN299). Data are presented as mean ± SD (n = 3, each group), **^**^***P* < 0.01 compared with NHA group. (D, E) Analysis of the glycolytic capacity and glycolytic reserve of Fig. 1J. Data are presented as mean ± SD (n = 3, each group). **^**^***P* < 0.01 compared with MBNL1(+)-NC group; **^##^***P* < 0.01 compared with shMBNL1-NC group. (F) mRNA expression levels of GLUT1 (glucose transporter 1), HK2, PFK1 (phosphofructokinase-1) and LDHA (lactate dehydrogenase A) in U251 and U373 with overexpression of MBNL1. Data are presented as mean ± SD (n = 3, each group), **^**^***P* < 0.01 compared with MBNL1(+)-NC group. (G) Expression levels of HK2 and PKM2 in U251 and U373 cells with MBNL1 overexpression or knockdown. IDVs of the bands were statistically analyzed. Data are presented as mean ± SD (n = 3, each group). **^*^***P* < 0.05 compared with MBNL1(+)-NC group; **^#^***P* < 0.05 compared with shMBNL1-NC group. (H) Effects of MBNL1 knockdown and 2-DG on proliferation of U251 and U373 cells were detected by cell proliferation assays. Data are presented as mean ± SD (n = 3, each group). **^**^***P* < 0.01 compared with shMBNL1-NC group; **^#^***P* < 0.05, compared with shMBNL1-#1 group; ^%^*P* < 0.05, compared with shMBNL1-#2 group.

**Supplementary figure 2**


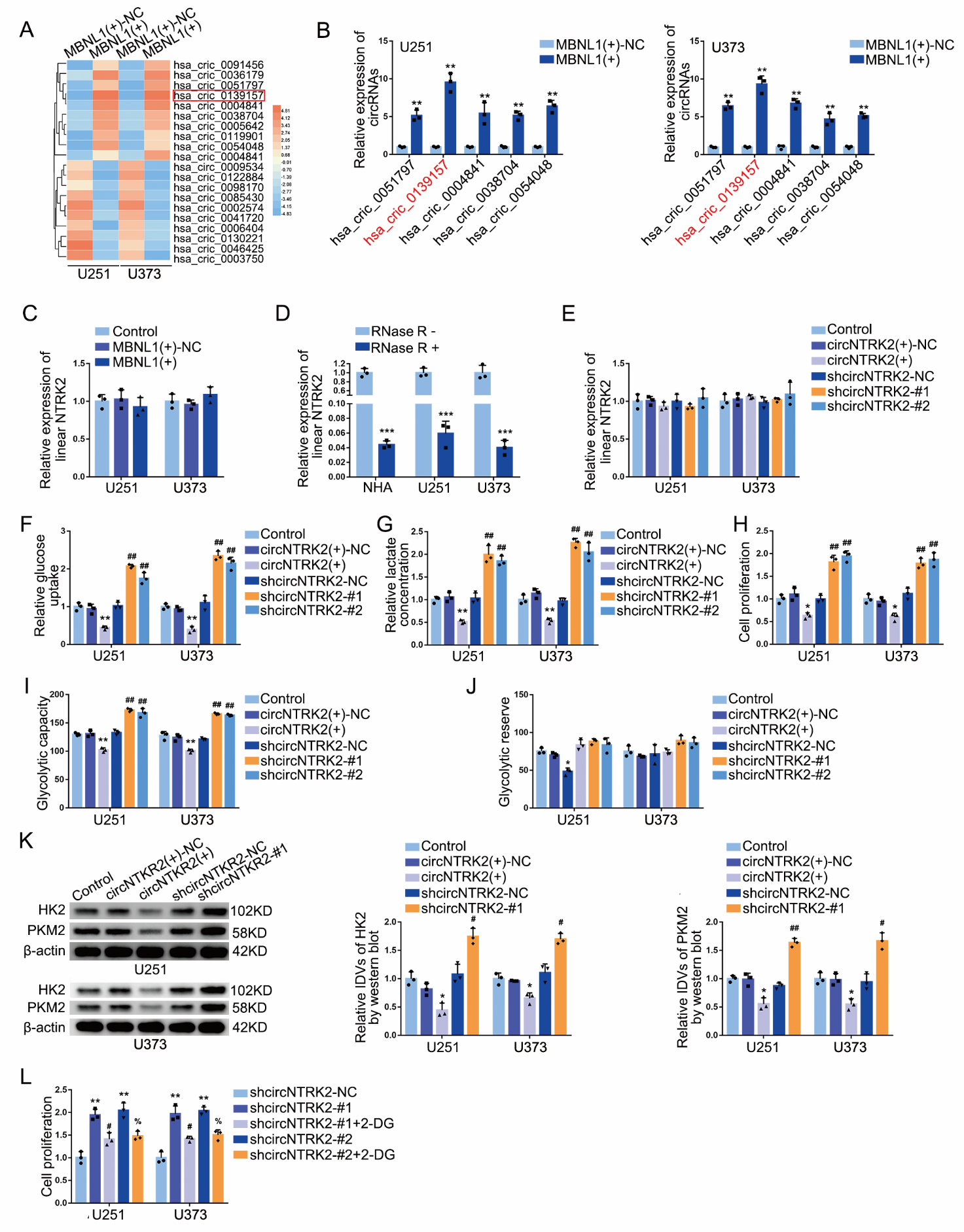


**Supplementary figure 2. CircNTRK2 exerted inhibitory effect on glycolysis and proliferation in GBM cells.**

(A) CircRNAs expression profiles obtained in MBNL1(+)-NC and MBNL1(+) groups as indicated in method. (B) qRT-PCR was conducted to validate the selected circNTRK2 in U251 and U373. Data are presented as mean ± SD (n = 3, each group). **^**^***P* < 0.01 compared with MBNL1(+)-NC group. (C) Expression level of linear NTRK2 upon MBNL1 overexpression in U251 and U373. Data are presented as mean ± SD (n = 3, each group). (D) Expression of linear NTRK2 treated with RNase R was detected by qRT-PCR. Data are manifested as mean ± SD (n = 3, each group). **^***^***P* < 0.001, compared with RNase R- group. (E) Detection of linear NTRK2 in U251 and U373 upon overexpression or knockdown of circNTRK2. Data are manifested as mean ± SD (n = 3, each group). (F–H) Effects of circNTRK2 overexpression or knockdown on glucose uptake, lactate production and proliferation in U251 and U373 cells were detected by glucose uptake, lactate and cell proliferation assays respectively. Data are presented as mean ± SD (n = 3, each group). **^*^***P* < 0.05, **^**^***P* < 0.01, compared with circNTRK2(+)-NC group; **^##^***P* < 0.01 compared with shcircNTRK2-NC group. (I, J) Analysis of the glycolytic capacity and glycolytic reserve of Figure 2G. Data are presented as mean ± SD (n = 3, each group). **^*^***P* < 0.05, **^**^***P* < 0.01 compared with circNTRK2(+)-NC group; **^##^***P* < 0.01 compared with shcircNTRK2-NC group. (K) Expression levels of HK2 and PKM2 in U251 and U373 cells upon circNTRK2 overexpression or knockdown. IDVs of the bands were statistically analyzed. Data are presented as mean ± SD (n = 3, each group). **^*^***P* < 0.05, compared with circNTRK2(+)-NC group; **^#^***P* < 0.05, **^##^***P* < 0.01 compared with shcircNTRK2-NC group. (L) Effects of circNTRK2 knockdown and 2-DG on proliferation of U251 and U373 cells were detected by cell proliferation assays. Data are presented as mean ± SD (n = 3, each group). **^**^***P* < 0.01 compared with shcircNTRK2-NC group; **^#^***P* < 0.05, compared with shcircNTRK2-#1 group; ^%^*P* < 0.05, compared with shcircNTRK2-#2 group.

**Supplementary figure 3**


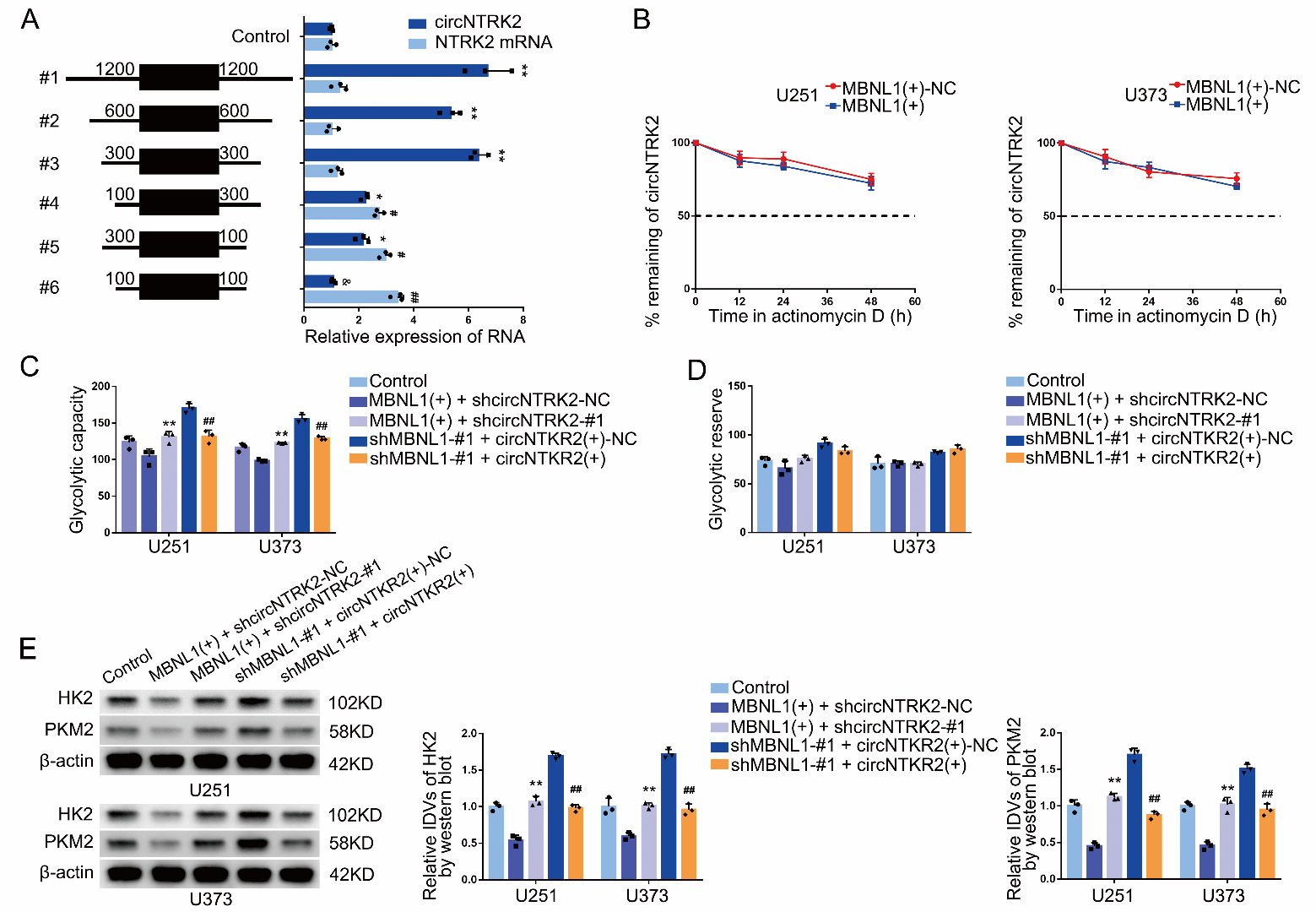


**Supplementary figure 3.** **Regulation of glycolysis and proliferation of GBM cells exerted by MBNL1 via promoting the expression of circNTRK2**

(A) Sequences in the intron downstream and upstream of circNTRK2 are necessary for circularization. The expression of circNTRK2 and *NTRK2* mRNA in HEK-293T cells with MBNL1 overexpression were detected by qRT-PCR. Black lines indicate circNTRK2 flanking intronic sequences and black boxes indicate exons between flanking intron. Data are presented as mean ± SD (n = 3, each group). **^*^***P* < 0.05, **^**^***P* < 0.01 compared with control circNTRK2 group. **^#^***P* < 0.05, **^##^***P* < 0.01 compared with control *NTRK2* mRNA group. ^&^*P* < 0.05 compared with #5 circNTRK2 group. (B) Half-life of circNTRK2 was measured in GBM cells upon overexpression of MBNL1. Data are presented as mean ± SD (n = 3, each group). (C, D) Analysis of the glycolytic capacity and glycolytic reserve of Figure 3F. Data are presented as mean ± SD (n = 3, each group). **^**^***P* < 0.01 compared with MBNL1(+) + shcircNTRK2-NC group; **^##^***P* < 0.01 compared with shMBNL1-#1 + circNTRK2(+)-NC group. (E) The reverse effect of circNTRK2 on MBNL1 in terms of HK2 and PKM2 expression levels. IDVs of the bands were statistically analyzed. Data are manifested as mean ± SD (n = 3, each group). **^**^***P* < 0.01, compared with MBNL1(+) + shcircNTRK2-NC group; **^##^***P* < 0.01 compared with shMBNL1-#1 + circNTRK2(+)-NC group.

**Supplementary figure 4**


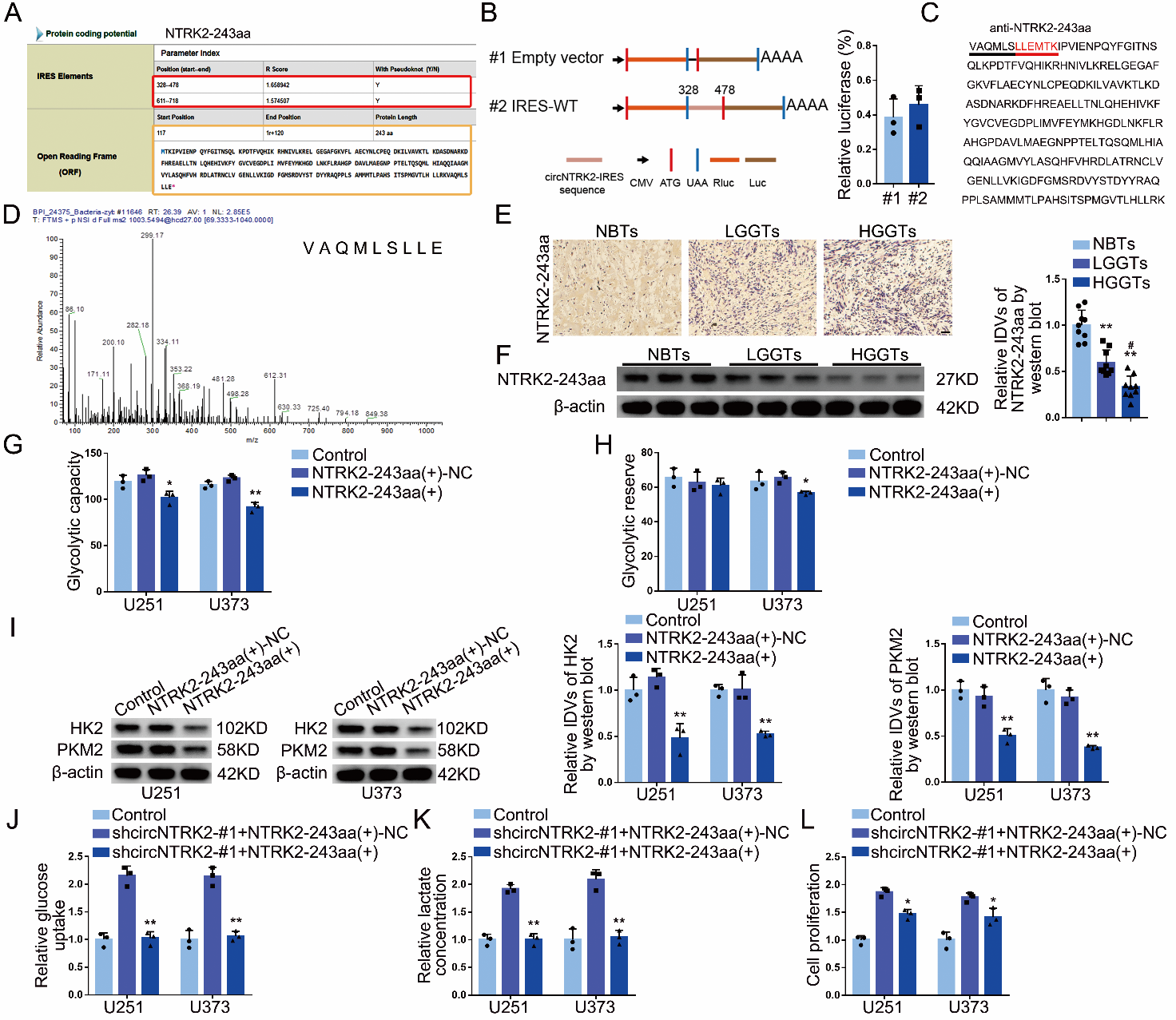


**Supplementary figure 4. Inhibitory effect of NTRK2-243aa on glycolysis and proliferation in GBM cells**

(A) CircNTRK2, containing ORF and IRES by scanning data of circRNADb, potentially encoded NTRK2-243aa. (B) The activity of IRES (328–478) in circNTRK2 was tested. CircNTRK2 IRES (328–478) wild type was cloned between Luc and Rluc with independent promotor and terminator on the both sides. Luc/Rluc relative luciferase activity was determined by Dual-Luciferase Reporter Assay kit in HEK-293T cells transfected with vectors above. Data are manifested as mean ± SD (n = 3, each group). (C) The amino acid sequence of NTRK2-243aa. The line represents the sequence used to produce the NTRK2-243aa antibody generated by Beijing Protein Invovation. The red amino acids represent the unique sequence formed by the circNTRK2 junction. (D) Sequence of NTRK2-243aa was validated by IP enrichment followed by mass spectrometry. (E) IHC shows the expression and distribution of NTRK2-243aa in normal brain tissues (NBTs), low grade glioma tissues (LGGTs) and high grade glioma tissues (HGGTs) (×200; scale bar represents 150 μm). (F) NBTs deriving from regions adjacent to the GBM, LGGTs and HGGTs were analyzed for NTRK2-243aa protein levels by western blotting. IDVs of the bands were statistically analyzed. Data are manifested as mean ± SD (n = 9). **^**^***P* < 0.01, compared with NBTs group; **^#^***P* < 0.05 compared with LGGTs group. (G–H) Analysis of the glycolytic capacity and glycolytic reserve of Fig. 4G. Data are presented as mean ± SD (n = 3, each group). **^*^***P* < 0.05, **^**^***P* < 0.01 compared with NTRK2-243aa(+)-NC group. (I) The expression of HK2 and PKM2 in U251 and U373 cells with the overexpression of NTRK2-243aa. IDVs of the bands were statistically analyzed. Data are presented as mean ± SD (n = 3, each group). **^**^***P* < 0.01 compared with NTRK2-243aa(+)-NC group. (J–L) The reverse effect of NTRK2-243aa on circNTRK2 in terms of glucose uptake, lactate production and proliferation. Data are presented as mean ± SD (n = 3, each group). **^*^***P* < 0.05, **^**^***P* < 0.01 compared with shcircNTRK2-#1 + NTRK2-243aa(+)-NC group.

**Supplementary figure 5**


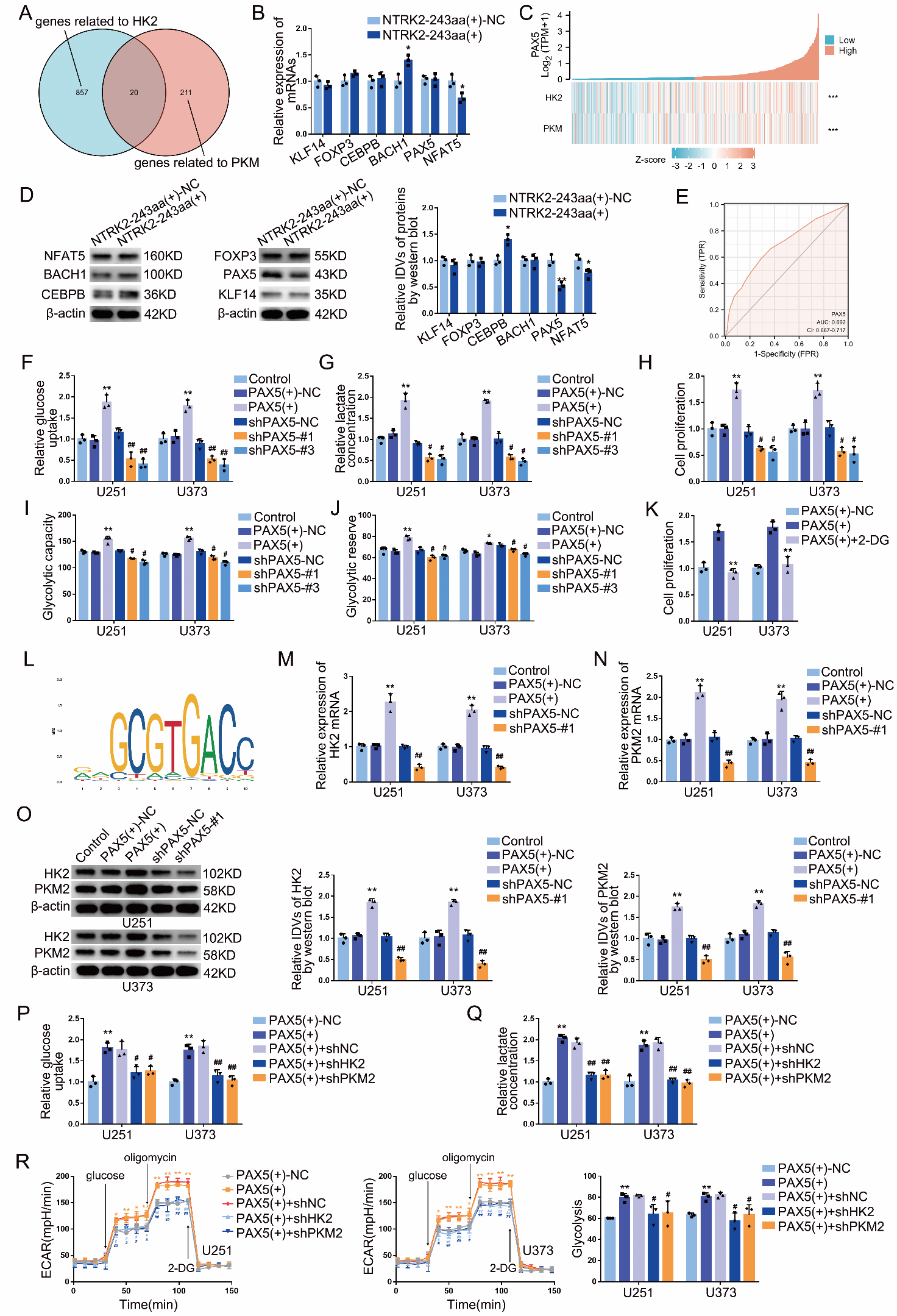


**Supplementary figure 5. PAX5 was selected as a research candidate, impeding glycolysis and proliferation of GBM cells via promoting the expression of HK2 and PKM2 at transcriptional level.**

(A) Venn diagram of genes related to *HK2* and *PKM*. (B) The mRNA expression of KLF14, FOXP3, CEBPB, BACH1, PAX5 and NFAT5 when upregulating NTRK2-243aa. Data are presented as mean ± SD (n = 3, each group). **^*^***P* < 0.05 compared with NTRK2-243aa(+)-NC group. (C) Co-expression correlation Heatmap of *PAX5*, *HK2* and *PKM*. (D) The protein expression of KLF14, FOXP3, CEBPB, BACH1, PAX5 and NFAT5 when overexpressing NTRK2-243aa. The IDVs of the bands were statistically analyzed. Data are presented as mean ± SD (n = 3, each group). **^*^***P* < 0.05, **^**^***P* < 0.01 compared with NTRK2-243aa(+)-NC group. (E) ROC curve of *PAX5* mRNA expression in glioma cohort of TCGA database. (F–H) Effects of PAX5 overexpression and knockdown on glucose uptake, lactate production and proliferation in U251 and U373 cells were measured by glucose uptake, lactate and cell proliferation assays respectively. Data are presented as mean ± SD (n = 3, each group). **^**^***P* < 0.01, compared with PAX5(+)-NC group; **^#^***P* < 0.05, **^##^***P* < 0.01, compared with shPAX5-NC group. (I, J) Analysis the glycolytic capacity and glycolytic reserve of Figure 5D. Data are presented as mean ± SD (n = 3, each group). **^**^***P* < 0.01 compared with PAX5(+)-NC group; **^#^***P* < 0.05 compared with shPAX5-NC group. (K) Effects of PAX5 overexpression and 2-DG on proliferation of U251 and U373 cells were detected by cell proliferation assays. Data are presented as mean ± SD (n = 3, each group). **^**^***P* < 0.01 compared with PAX5(+) group. (L) Analysis of transcription factor PAX5 binding sites by JASPAR database. (M, N) mRNA expression levels of *HK2* and *PKM2* in U251 and U373 with PAX5 overexpression or knockdown. Data are presented as mean ± SD (n = 3, each group), **^**^***P* < 0.01 compared with PAX5(+)-NC group; **^##^***P* < 0.01 compared with shPAX5-NC group. (O) Expression levels of HK2 and PKM2 in U251 and U373 cells with PAX5 overexpression or knockdown. The IDVs of the bands were statistically analyzed. Data are presented as mean ± SD (n = 3, each group). **^**^***P* < 0.01 compared with PAX5(+)-NC group; **^##^***P* < 0.01 compared with shPAX5-NC group. (P–R) Effects of HK2 or PKM2 knockdown on glucose uptake, lactate production and ECAR of U251 and U373 cells overexpressing PAX5 were detected by glucose uptake, lactate assays and Searhorse respectively. Data are presented as mean ± SD (n = 3, each group). **^**^***P* < 0.01 compared with PAX5(+)-NC group; **^#^***P* < 0.05, **^##^***P* < 0.01 compared with PAX5(+)-NC + shNC group.

**Supplementary figure 6**


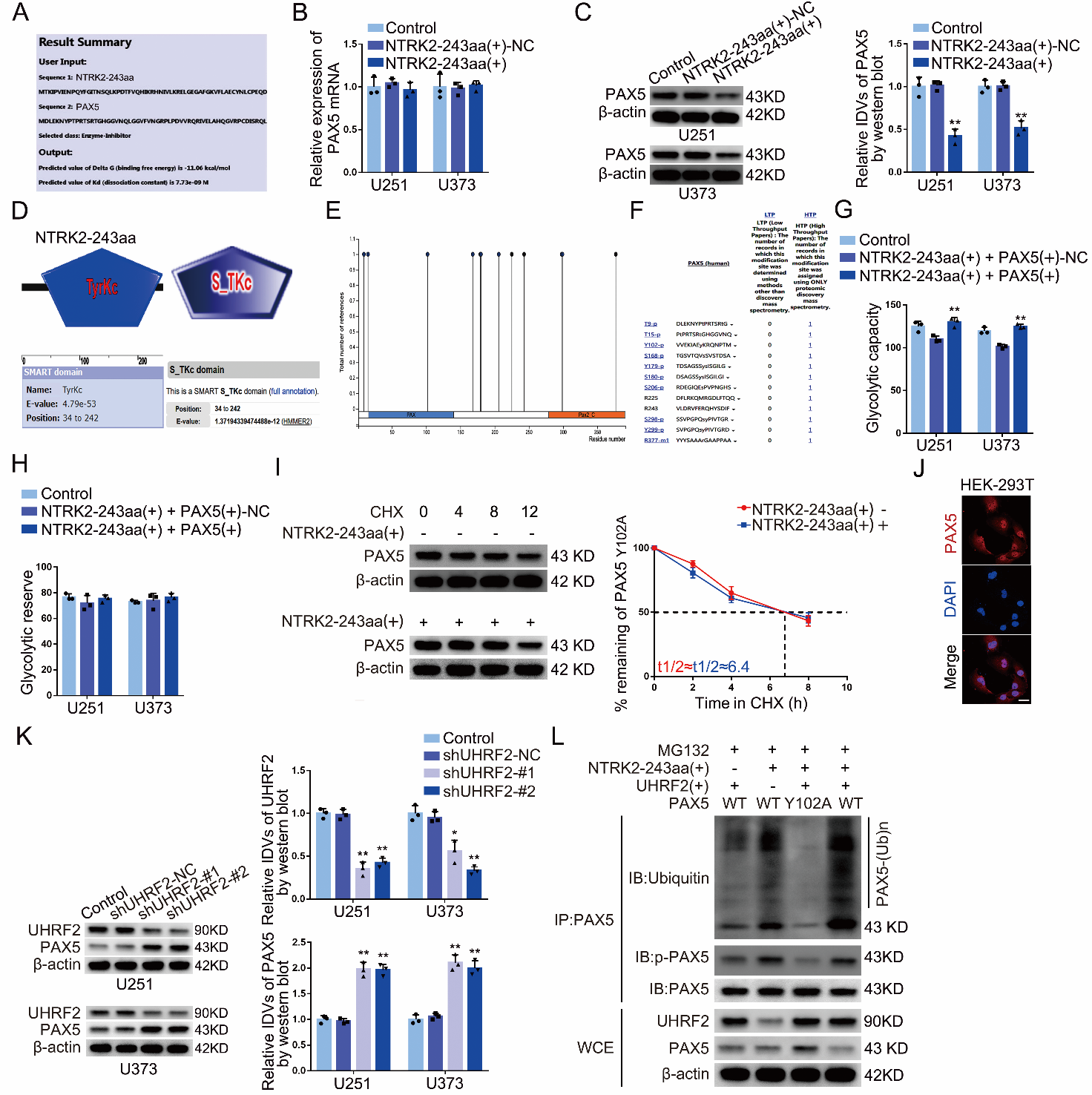


**Supplementary figure 6.** **Interaction between NTRK2-243aa and PAX5.**

(A) The interaction of NTRK2-243aa and PAX5 was predicted by PPA-Pred2 database. (B) The mRNA expression of PAX5 in U251 and U373 with upregulating NTRK2-243aa. Data are presented as mean ± SD (n = 3, each group). (C) The protein expression of PAX5 in U251 and U373 upon NTRK2-243aa overexpression. The IDVs of the bands were statistically analyzed. Data are presented as mean ± SD (n = 3, each group). **^**^***P* < 0.01 compared with NTRK2-243aa(+)-NC group. (D) Kinase domains, like TyrKc and S_TKc, are predicted in NTRK2-243aa by SMART database. (E, F) Phosphorylation sites were predicted in PAX5 by PhosphoSitePlus database. (G, H) Analysis glycolytic capacity and glycolytic reserve of Figure 6F. Data are presented as mean ± SD (n = 3, each group). **^**^***P* < 0.01 compared with NTRK2-243aa(+) + PAX5(+)-NC group. (I) Half-life of PAX5 protein was measured in HEK-293T cells upon overexpression of NTRK2-243aa and PAX5 with the sited mutations of Y102A after treatment with CHX. The IDVs of the bands were statistically analyzed. Data are presented as mean ± SD (n = 3, each group). (J) The distribution of PAX5 in HEK-293T cells was determined by IF assay (PAX5 was labeled in red by secondary antibody against anti-PAX5 antibody and nuclei were labeled in blue by DAPI. Scale bar represents 10 μm). (K) Expression levels of UHRF2 and PAX5 were detected by western blot assays. The IDVs of the bands were statistically analyzed. Data are presented as mean ± SD (n = 3, each group). **^*^***P* < 0.05, **^**^***P* < 0.01 compared with shUHRF2-NC group. (L) PAX5 ubiquitination level, expression level of p-PAX5, using a polyclonal antibody that targets the Y102-phosphorylated PAX5, and UHRF2 was measured with upregulating NTRK2-243aa and UHRF2 after treatment with 20μM MG132 for 8 h. WCE represents the whole cell extract.

**Supplementary figure 7**


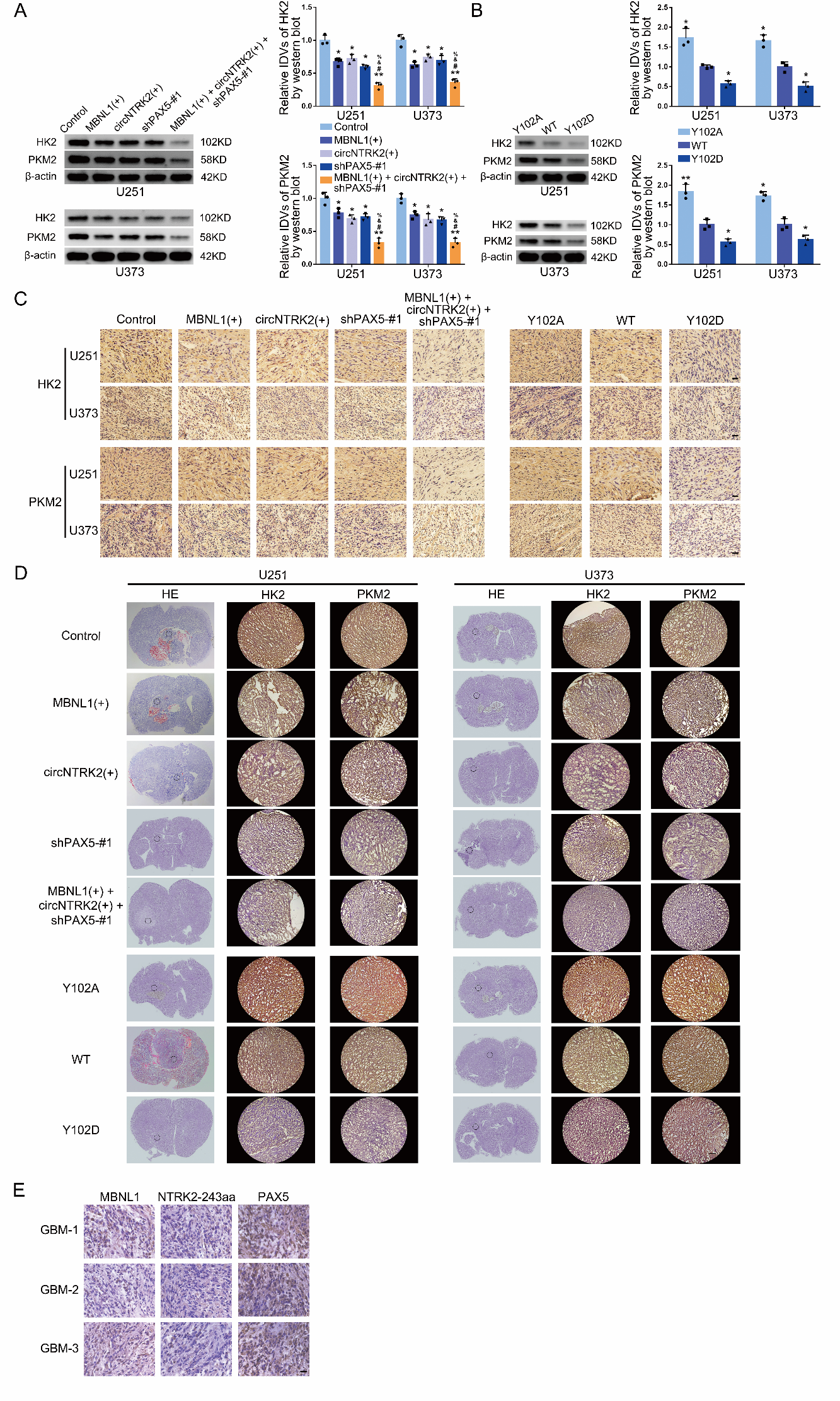


**Supplementary figure 7. HK2 and PKM2 detection of heterotopic graft**

(A) Expression levels of HK2 and PKM2 in sample tumors of MBNL1(+) group, circNTKR2(+) group, shPAX5-#1 group, and MBNL1(+) + circNTRK2(+) + shPAX5-#1 group. The IDVs of the bands were statistically analyzed. Data are presented as mean ± SD (n = 3). **^*^***P* < 0.05, **^**^***P* < 0.01 compared with Control group; **^#^***P* < 0.05, compared with MBNL1(+) group; ^&^*P* < 0.05 compared with circNTRK2(+) group, ^%^*P* < 0.05 compared with shPAX5-#1 group. (B) Expression levels of HK2 and PKM2 in sample tumors of Y102A group, simulating dephosphorylated state of tyrosine, and Y102D group, simulating phosphorylated state of tyrosine. The IDVs of the bands were statistically analyzed. Data are presented as mean ± SD (n = 3). **^*^***P* < 0.05, **^**^***P* < 0.01 compared with WT group. (C) IHC shows the expression and distribution of HK2 and PKM2 in subcutaneous xenografts (×200; scale bar represents 150 μm). (D) H&E images of whole brain slice containing the orthotopic xenograft were performed and the gated fields of adjacent slices were magnified in the IHC for HK2 and PKM2 (×200; scale bar represents 150 μm). (E) IHC shows the expression and distribution of MBNL1, NTRK2-243aa, and PAX5 in GBM patients’ tissues (×400; scale bar represents 70 μm).

**Supplementary figure 8**


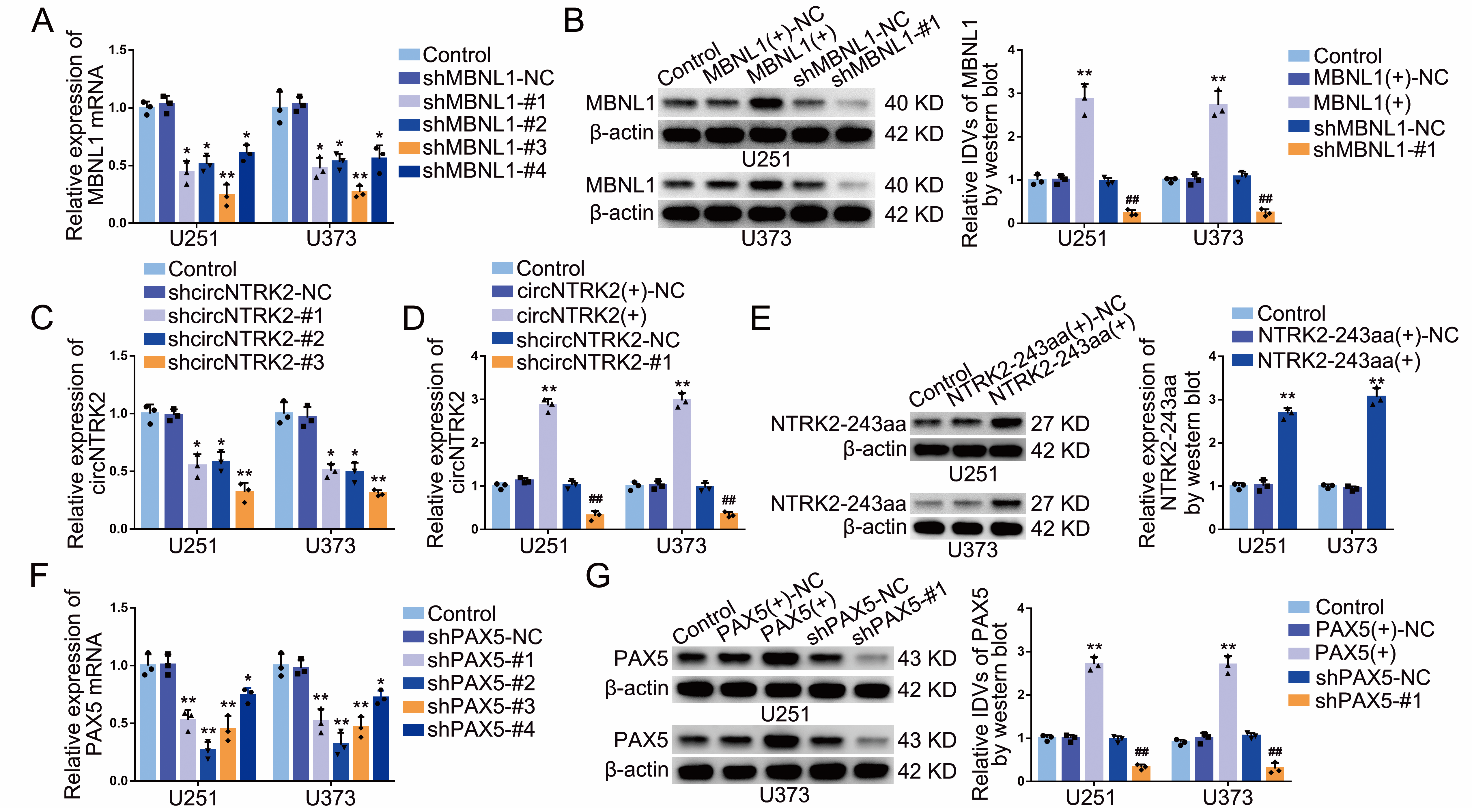


**Supplementary figure 8:** **Transfection efficiency of MBNL1, circNTRK2, NTRK2-243aa and PAX5**

(A) Efficiency of transient MBNL1 knockdown at four different sites. Data are presented as the mean ± SD (n=3, each group). **^*^***P* < 0.05, **^**^***P* < 0.01 compared with shMBNL1-NC group. (B) Efficiency of stable transfection of MBNL1(+) and shMBNL1-#1. IDVs of the bands were statistically analyzed. Data are presented as the mean ± SD (n=3, each group). **^**^***P* < 0.01 compared with MBNL1(+)-NC group; **^##^***P* < 0.01 compared with shMBNL1-NC group. (C) Efficiency of transient circNTRK2 knockdown using three different shRNAs against the junction sequence. Data are presented as the mean ± SD (n=3, each group). **^*^***P* < 0.05, **^**^***P* < 0.01 compared with shcircNTRK2-NC group. (D) Efficiency of stable transfection of circNTRK2(+) and shcircNTRK2-#1. Data are presented as the mean ± SD (n=3, each group). **^**^***P* < 0.01 compared with circNTRK2(+)-NC group; **^##^***P* < 0.01 compared with shcircNTRK2-NC group. (E) Efficiency of stable NTRK2-243aa overexpression. IDVs of the bands were statistically analyzed. Data are presented as the mean ± SD (n=3, each group). **^**^***P* < 0.01 compared with NTRK2-243aa(+)-NC group. (F) Efficiency of PAX5 transient knockdown in four different sites. Data are presented as the mean ± SD (n=3, each group). **^*^***P* < 0.05, **^**^***P* < 0.01 compared with shPAX5-NC group. (G) Efficiency of stable transfection of PAX5(+) and shPAX5-#1. IDVs of the bands were statistically analyzed. Data are presented as the mean ± SD (n=3, each group). **^**^***P* < 0.01 compared with PAX5(+)-NC group; **^##^***P* < 0.01 compared with shPAX5-NC group.

**Supplementary Table**

**Supplementary table 1**

Clinical and Gene Expression Data of MBNL1 from TCGA Database

| Characteristic | levels | Low expression of MBNL1 | High expression of MBNL1 | p |
| --- | --- | --- | --- | --- |
| n |  | 348 | 348 |  |
| WHO grade, n (%) | G2 | 123 (19.4%) | 101 (15.9%) | 0.086 |
|  | G3 | 112 (17.6%) | 131 (20.6%) |  |
|  | G4 | 76 (12%) | 92 (14.5%) |  |
| IDH status, n (%) | WT | 138 (20.1%) | 108 (15.7%) | 0.021 |
|  | Mut | 205 (29.9%) | 235 (34.3%) |  |
| 1p/19q codeletion, n (%) | codel | 57 (8.3%) | 114 (16.5%) | < 0.001 |
|  | non-codel | 288 (41.8%) | 230 (33.4%) |  |
| Primary therapy outcome, n (%) | PD | 43 (9.3%) | 69 (14.9%) | < 0.001 |
|  | SD | 64 (13.9%) | 83 (18%) |  |
|  | PR | 37 (8%) | 27 (5.8%) |  |
|  | CR | 88 (19%) | 51 (11%) |  |
| Gender, n (%) | Female | 143 (20.5%) | 155 (22.3%) | 0.399 |
|  | Male | 205 (29.5%) | 193 (27.7%) |  |
| Race, n (%) | Asian | 8 (1.2%) | 5 (0.7%) | 0.606 |
|  | Black or African American | 18 (2.6%) | 15 (2.2%) |  |
|  | White | 316 (46.3%) | 321 (47%) |  |
| Age, n (%) | <=60 | 279 (40.1%) | 274 (39.4%) | 0.707 |
|  | >60 | 69 (9.9%) | 74 (10.6%) |  |
| Age, meidan (IQR) |  | 45 (34, 58) | 47 (34, 59) | 0.397 |

**Supplementary table 2**

| Characteristic | levels | Low expression of PAX5 | High expression of PAX5 | p |
| --- | --- | --- | --- | --- |
| n |  | 348 | 348 |  |
| WHO grade, n (%) | G2 | 124 (19.5%) | 100 (15.7%) | < 0.001 |
|  | G3 | 109 (17.2%) | 134 (21.1%) |  |
|  | G4 | 62 (9.8%) | 106 (16.7%) |  |
| IDH status, n (%) | WT | 93 (13.6%) | 153 (22.3%) | < 0.001 |
|  | Mut | 250 (36.4%) | 190 (27.7%) |  |
| 1p/19q codeletion, n (%) | codel | 11 (1.6%) | 160 (23.2%) | < 0.001 |
|  | non-codel | 333 (48.3%) | 185 (26.9%) |  |
| Primary therapy outcome, n (%) | PD | 53 (11.5%) | 59 (12.8%) | 0.363 |
|  | SD | 58 (12.6%) | 89 (19.3%) |  |
|  | PR | 31 (6.7%) | 33 (7.1%) |  |
|  | CR | 68 (14.7%) | 71 (15.4%) |  |
| Gender, n (%) | Female | 142 (20.4%) | 156 (22.4%) | 0.319 |
|  | Male | 206 (29.6%) | 192 (27.6%) |  |
| Race, n (%) | Asian | 4 (0.6%) | 9 (1.3%) | 0.334 |
|  | Black or African American | 18 (2.6%) | 15 (2.2%) |  |
|  | White | 319 (46.7%) | 318 (46.6%) |  |
| Age, n (%) | <=60 | 278 (39.9%) | 275 (39.5%) | 0.851 |
|  | >60 | 70 (10.1%) | 73 (10.5%) |  |
| Age, meidan (IQR) |  | 45 (33, 59) | 45.5 (35, 59) | 0.520 |

Clinical and Gene Expression Data of PAX5 from TCGA Database

| Primer or Probe | Gene | Sequence (5’->3’) |
| --- | --- | --- |
| Primer | MBNL1 | F: GTTGCACCAAGCTTAGCCAC |
|  |  | R: AGGTCAAAGGTTGCCTCGAG |
|  | circNTRK2 (has_circ_0139157) | F: ACCTCCAGCATGAGCACATC |
|  |  | R: GTACACGTCCCGGGACATC |
|  | hsa_circ_0051797 | F: TTCCGAGTGGCAGCTGACA |
|  |  | R: GCCCGGGTCACGTGAAGC |
|  | hsa_circ_0004841 | F: TCCAGCATTTCAGGGAGCAG |
|  |  | R: AAAGGGCGCTCATTCACGAT |
|  | hsa_circ_0038704 | F: CGTGAATGAGAAAGCAAGGGC |
|  |  | R: TCAGCCACGCCTTTCAGGAT |
|  | hsa_circ_0054048 | F: CCAGTCAGTGCAGCTCTTCA |
|  |  | R: TCGAGCCGATTTCAACCTGG |
|  | β-actin | F: GTGCTATCCCTGTACGCCTC |
|  |  | R: AATGCCAGGGTACATGGTGG |
|  | linear NTRK2 | F: TCTGCTCACTTCATGGGCTG |
|  |  | R: AGACCGAGAGATGTTCCCGA |
|  | NTRK2 pre-mRNA | F: GTACGGCAGGGGAGCAAG |
|  |  | R: TGCGCTGATTCTTATTATAGGAA |
|  | NTRK2 pre-mRNA MUT | F: ACTTTGCCATGGGATTGATCC |
|  |  | R: ACACTAGAACGAGATTAGTAACAGGA |
|  | β-actin pre-mRNA | F: CTACCTCTTCTGGTGGCCG |
|  |  | R: CACCATGTCACACTGGGGAA |
|  | IR pre-mRNA | F: CCCGTTCTGCCCAAGAATCT |
|  |  | R: GGGAACGAAAGGCTCACTCA |
|  | circNTRK2 convergent primers | F: ACCTCCAGCATGAGCACATC |
|  |  | R: CTTGTTGAGGTCCCCATGCT |
|  | circNTRK2 divergent primers | F: CCAGCCTCCGTTATCAGCAA |
|  |  | R: TGTCTGGCTTGAGCTGACTG |
|  | PAX5 | F: ACAGCATAGTGTCCACTGGC |
|  |  | R: ATAGTAGGGGGAGCCAAGCA |
|  | KLF14 | F: GAGTGCCTGGTGTCCATGTC |
|  |  | R: CAGCAGGTGTGACAGACCTC |
|  | FOXP3 | F: CACTGCTGGCAAATGGTGTC |
|  |  | R: AGGCAAACATGCGTGTGAAC |
|  | CEBPB | F: CAAGAACTGCAAGAAGCCGG |
|  |  | R: TGCTCCACCTTCTTCTGCAG |
|  | BACH1 | F: AGATGTGCTGTGCGATGTCA |
|  |  | R: AGAGGAGGTGAGGCTTTTGC |
|  | NFAT5 | F: CCGAAGAGGCACACAGTCTT |
|  |  | R: GAGTCGTTGCCCACAAACAC |
|  | HK2 | F: GATTGCCTCGCATCTGCTTG |
|  |  | R: CAACGTCTCTGCCTTCCACT |
|  | PKM2 | F: TCATTCAGACCCAGCAGCTG |
|  |  | R: AGGAAGTCGGCACCTTTCTG |
| Probe | circNTRK2 | CY3-TGACTGATGATGTCCCGGGT |

**Supplementary table 3.** Primers used for qRT-PCR and the probe for FISH assays

**Supplementary table 4.** Antibodies used for western blotting, co-IP, nascent RIP, IF and ChIP

| **Protein** | **Applications** | **Antibody** | **Origin** | **Dilution** | **Observed molecular weight** |
| --- | --- | --- | --- | --- | --- |
| MBNL1 | WB, IP, IF | 66837-1-Ig, Proteintech | Mouse | 1:2000;1μg per IP;1:50 | 40 kD |
| NTRK2-243aa | WB, IP, IF | 243-Pep1(196-210aa)-KLH, Beijing protein invovation | Rabbit | 1:500;1μg per IP;1:50 | 27 kD |
| PAX5 | WB, IP, IF | 60349-1-Ig, Proteintech | Mouse | 1:1000;1μg per IP;1:50 | 43 kD |
| β-actin | WB | 66009-1-Ig, Proteintech | Mouse | 1:10000 | 42 kD |
| IgG | RIP | ab18413, Abcam | Mouse | 1:10 | 150 kD |
| HK2 | WB | 66974-1-Ig, Proteintech | Mouse | 1:10000 | 102 kD |
| PKM2 | WB | 60268-1-Ig, Proteintech | Rabbit | 1:5000 | 58 kD |
| Goat anti-mouse IgG (H+L), HRP conjugate | WB | SA00001-1, Proteintech |  | 1:10000 | None |
| Goat anti-rabbit IgG (H+L), HRP conjugate | WB | SA00001-2, Proteintech |  | 1:10000 | None |
| anti-FLAG | WB | 80010-1-RR, Proteintech | Rabbit | 1:2000 | None |
| ubiquitin | WB | 10201-2-AP, Proteintech | Rabbit | 1:1000 | None |
| NFAT5 | WB | bs-9473R, Bioss | Rabbit | 1:1000 | 160 kD |
| BACH1 | WB | 14018-1-AP, Proteintech | Rabbit | 1:500 | 100 kD |
| CEBPB | WB | 23431-1-AP, Proteintech | Rabbit | 1:500 | 36 kD |
| FOXP3 | WB | 22228-1-AP, Proteintech | Rabbit | 1:1000 | 55 kD |
| KLF14 | WB | #DF2594-BP, Affinity | Rabbit | 1:1000 | 35 kD |
| UHRF2 | WB | orb165659, Biorbyt | Rabbit | 1:1000 | 90 kD |
| Alexa-Fluor-488-labeled Goat anti-Mouse IgG (H+L) | IF | A0428, Proteintech | Goat | 1:500 | None |
| Alexa-Fluor-488-labeled Goat anti-Rabbit IgG (H+L) | IF | A0423, Proteintech | Goat | 1:500 | None |
| Alexa-Fluor-555-labeled Donkey anti-Mouse IgG (H+L) | IF | A0460, Proteintech | Donkey | 1:500 | None |
| Alexa-Fluor-555-labeled Donkey anti-Rabbit IgG (H+L) | IF | A0453, Proteintech | Donkey | 1:500 | None |

**Supplementary table 5.** shRNA used for transfection

| Gene | Target Sequence (5'->3') |
| --- | --- |
| shMBNL1-#1  shMBNL1-#2  shMBNL1-#3  shMBNL1-#4 | CATTTGCAAGCCAAGATCAAG  TTAACCAGAAGAAAAGACAAC  GGTTAGAGTAAAGGACGAGGT  CCAGACACGGAATGTAAATTTGC |
| shcircNTRK2-#1  shcircNTRK2-#2  shcircNTRK2-#3 | CACTGACTACTACAGGGCCCA  CTACAGGGCCCAGCCTCCGTTA  GACTACTACAGGGCCCAGCCT |
| shPAX5-#1 | GCCGACACCAACAAGCGCAAG |
| shPAX5-#2 | GGTAATTGGAGGATCCAAACC |
| shPAX5-#3  shPAX5-#4  shUHRF2-#1  shUHRF2-#2 | GGTGCTGGACCGCGTGTTTGA  CTAGACAATCAGTCTGTAAGAAC  CTGCTGATGAAGACGTTATTT  GTTGGTGATGTGGTAATGGTT |

**Supplementary table 6.** Primers used for ChIP experiments

| Gene | Binding or control sites | Sequence (5’->3’) | Product size (bp) | Annealing temperature (°C) |
| --- | --- | --- | --- | --- |
| HK2 | PCR1 | F: GGGTAGAAATATGAGTGCAGAGC | 105 | 53.7 |
|  |  | R: TACATCAGGAGCAGAGGCAC |  |  |
|  | PCR2 | F: GCTGGTCATGAACTCCTGGT | 127 | 55.4 |
|  |  | R: GTGAGTTTCACTAACCCGTTT |  |  |
|  | PCR3 | F: ACAATCAGCAGCTAAGGCGG | 114 | 62.1 |
|  |  | R: TGGCATCTCGGGATCATGTG |  |  |
|  | PCR4 | F: TTTTTCCAGTCGCCCCACAC | 129 | 60.3 |
|  |  | R: CTGAGATGGGACGTGTGGTC |  |  |
| PKM | PCR1 | F: GTCCGACCAACTTCAATTTGGG | 151 | 57.5 |
|  |  | R: CTGGCCCTGTGCATCTCTT |  |  |
|  | PCR2 | F: GATGGCCTCGATCTCCTGAC | 106 | 54.9 |
|  |  | R: AGATCAAGCTTGGAGGCCTG |  |  |
|  | PCR3 | F: GATGGTCTCAACTCCTGGCC | 130 | 61.4 |
|  |  | R: CTGGGATGGCAAGGATCTGG |  |  |

**Supplementary table 7.** Wild-type and mutant plasmid sequences

| Gene | plasmid sequences |
| --- | --- |
| IRES (611-718)-WT | CCAGCACTTCGTGCACCGCGATTTGGCCACCAGGAACTGCCTGGTCGGGGAGAACTTGCTGGTGAAAATCGGGGACTTTGGGATGTCCCG GGACGTGTACAGCACTGA |
| IRES (611-718)-mut | TTCATCTGGTAGATCTTATACGGGAATTCTTCAACCTGATTGAAGTAAAACACCTGGATGAAGACCCCGTAAAACTGGGAAACGAGTTTA AACTAGAGCTCATCTGAC |
| IRES (611-718)-DE-1 | CCAGCACTTCGTGCACCGCGATTTGGCCACCAGGAACTGCCTGGTCGGGGAGAA |
| IRES (611-718)-DE-2 | CTTGCTGGTGAAAATCGGGGACTTTGGGATGTCCCG GGACGTGTACAGCACTGA |
| IRES (328-478)-WT | AGGATGCCAGTGACAATGCACGCAAGGACTTCCACCGTGAGGCCGAGCTCCTGACCAACCTCCAGCATGAGCACATCGTCAAGTTCTATGGCGTCTGCGTGGAGGGCGACCCCCTCATCATGGTCTTTGAGTACATGAAGCATGGGGACCT |
| IRES (328-478)-mut | CAACGATTCAGACTCCGATCTATCCAACTGGTTCTTAGACAATTACATGTTGACTTCCTTGTTCATCGACATCTCGTAGTCCAGGTGCGAATAGTGATAGAACAAATACTTTTTGTCGTCGAAGTGGGACAGCTCGACCATCGAAAACTTG |
| PAX5 WT | GTGGAAAAAATCGCTGAATATAAACGCCAAAATCCCACCAT |
| PAX5 Y102A | GTGGAAAAAATCGCTGAAGCTAAACGCCAAAATCCCACCAT |
| PAX5 Y102D | GTGGAAAAAATCGCTGAAGATAAACGCCAAAATCCCACCAT |
| NTRK2 pre-mRNA | AATTTATAATACATTTTCCTGTTACTAATCTC |
| NTRK2 pre-mRNA MUT | AATTTATAATACATTTTCCTGTGCTGCATCTC |
