# Supplementary material for: The MBNL1/circNTRK2/PAX5 pathway regulates aerobic glycolysis in glioblastoma cells by encoding a novel protein NTRK2-243aa
Source: Cell Death Dis. 2022 Sep 5;13(9):767. doi: 10.1038/s41419-022-05219-4 (PMC9445070; doi:10.1038/s41419-022-05219-4)

Original western blots of 'The MBNL1/circNTRK2/PAX5 pathway regulates aerobic glycolysis in glioblastoma cells by encoding a novel protein NTRK2-243aa'

Figure 1D representative image

MBNL1

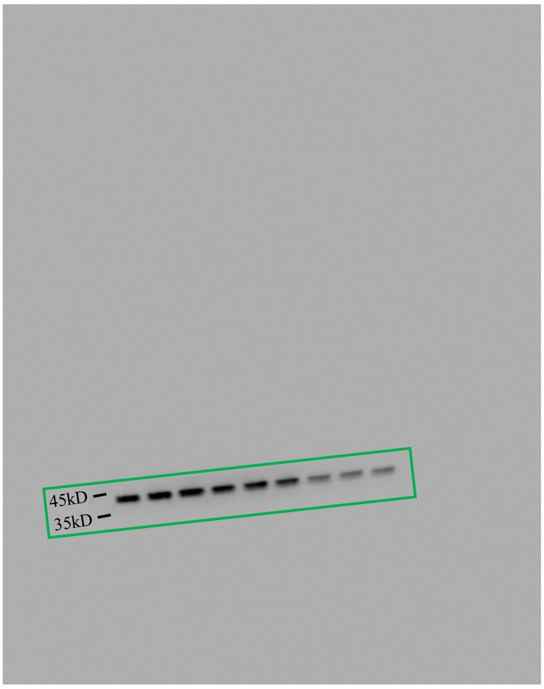

Figure 1D group 1 representative image

β-actin

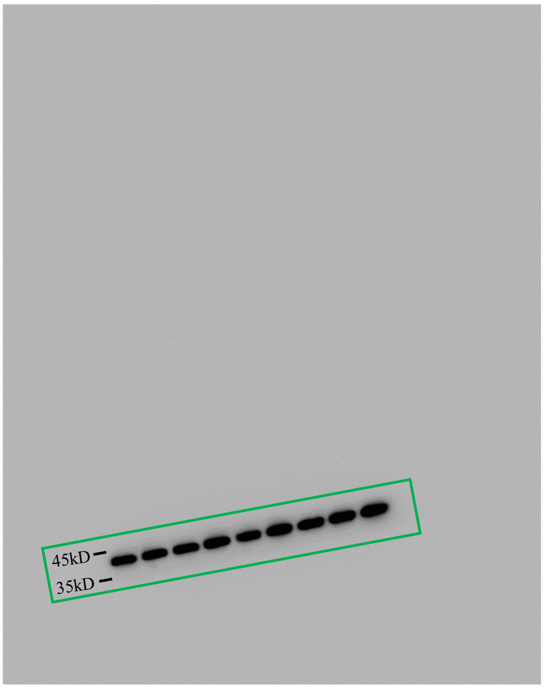

Figure 1E representative image

MBNL1

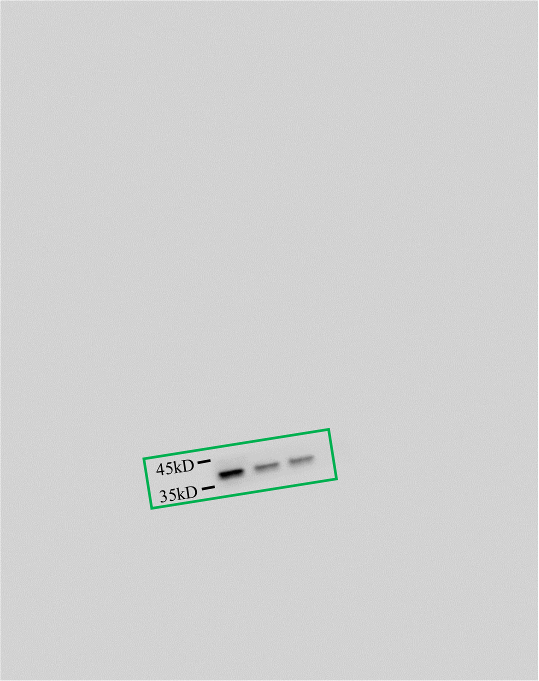

Figure 1E representative image

β-actin

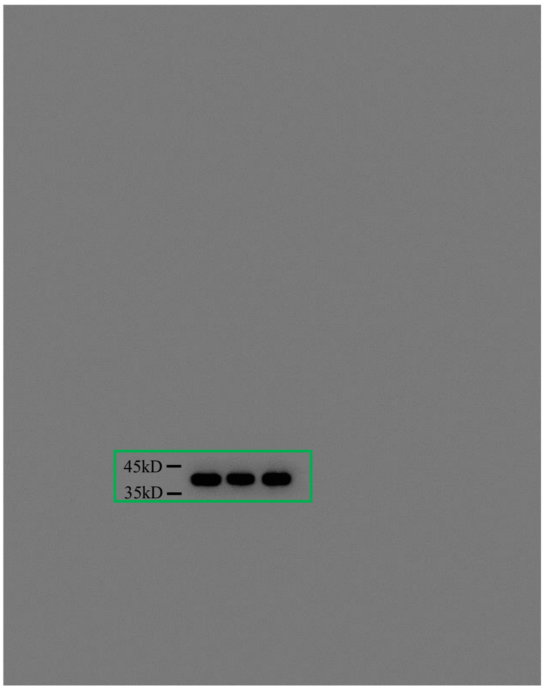

Figure 3A representative image

MBNL1

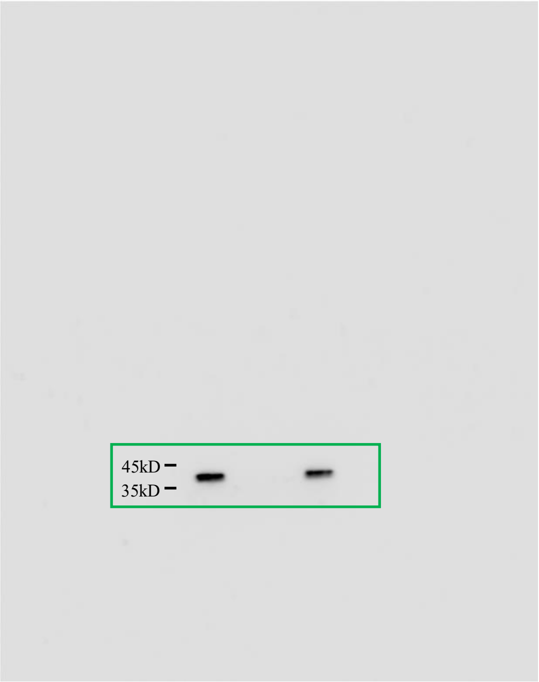

β-actin

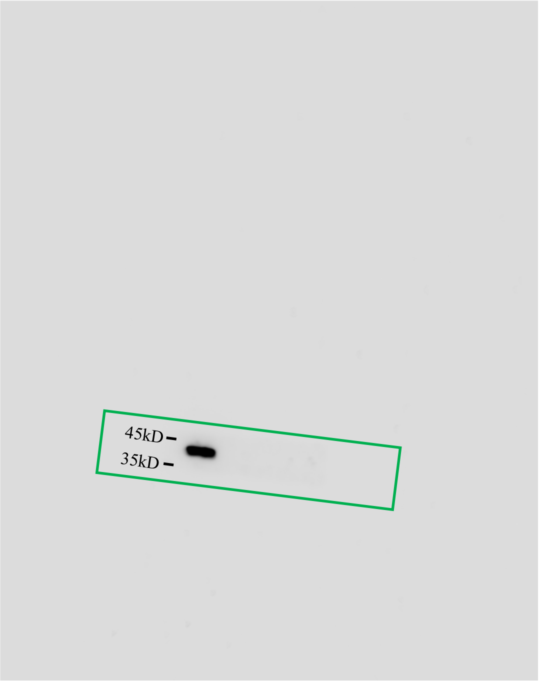

Figure 4B representative image

FLAG

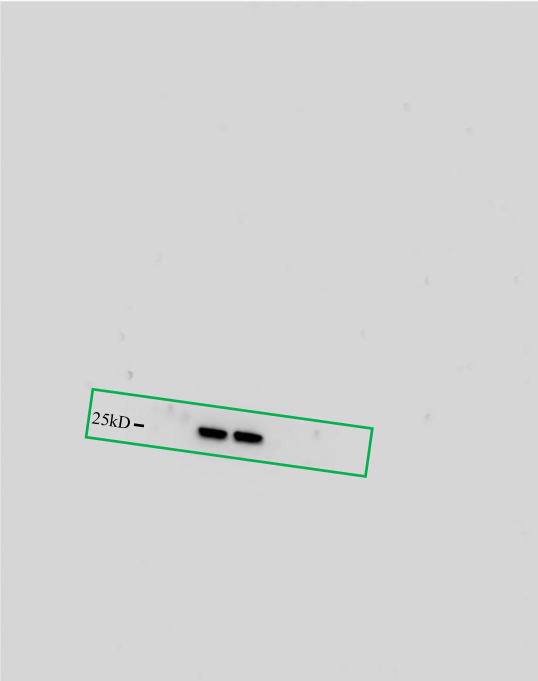

NTRK2-243aa

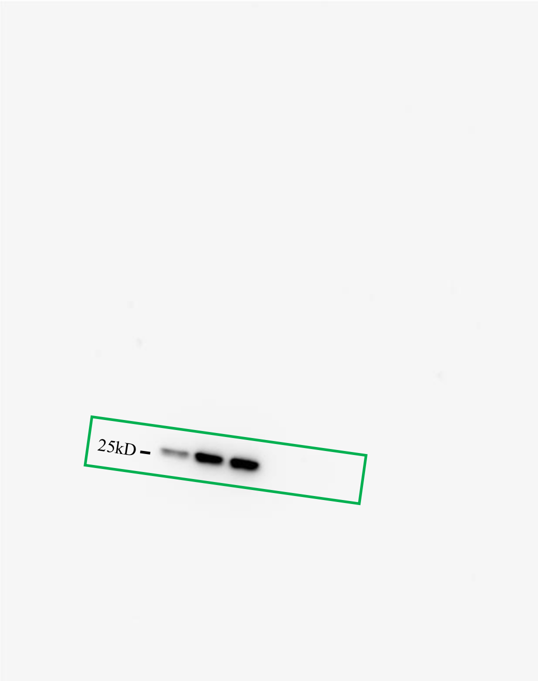

β-actin

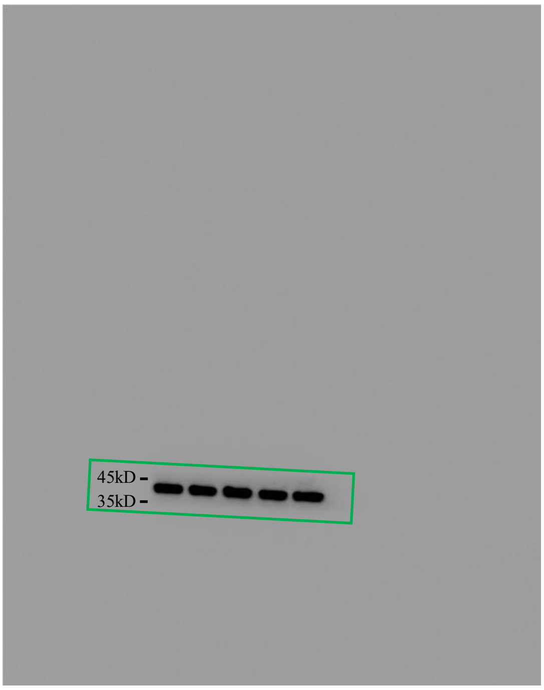

Figure 4C representative image

NTRK2-243aa

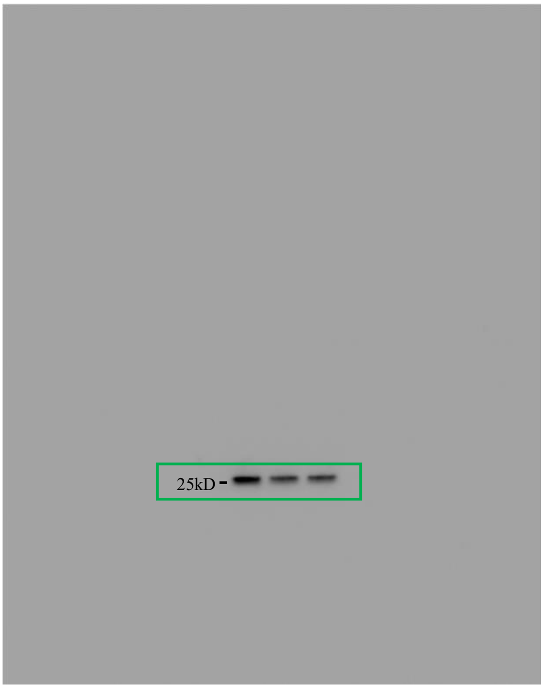

β-actin

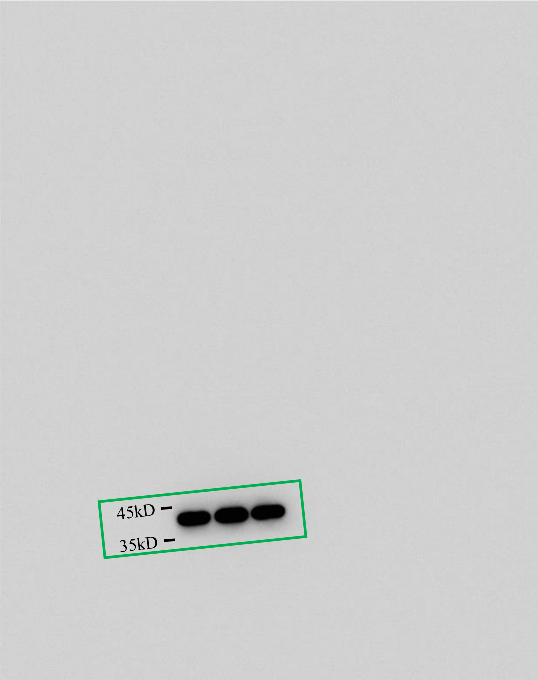

Figure 5B representative image

PAX5

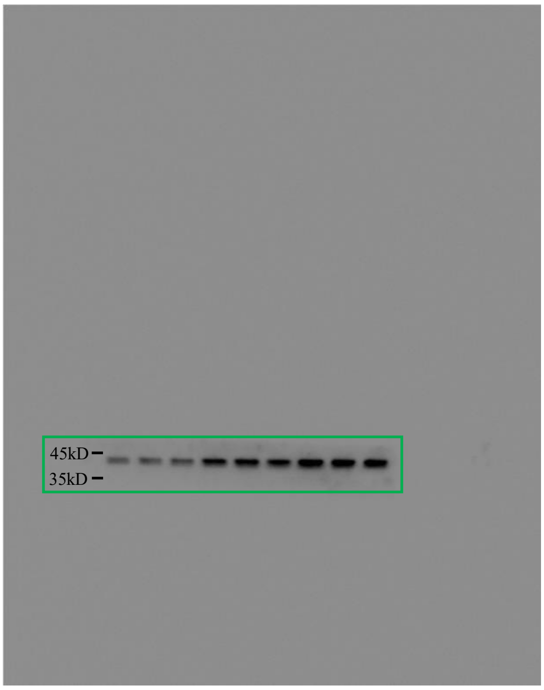

β-actin

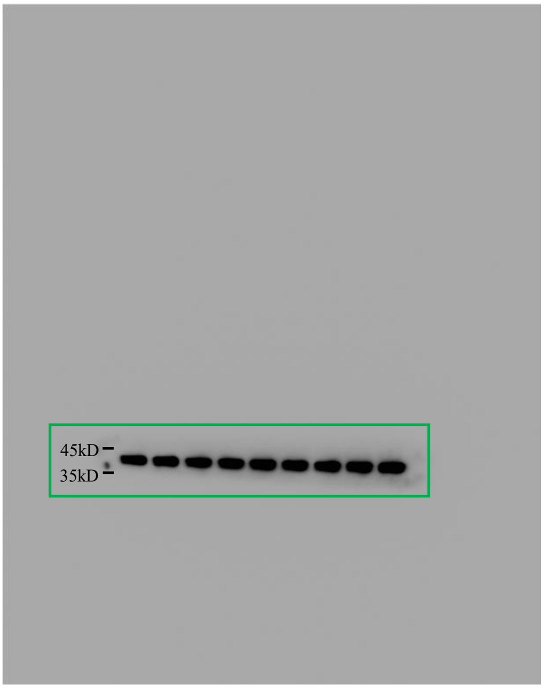

Figure 5C representative image

PAX5

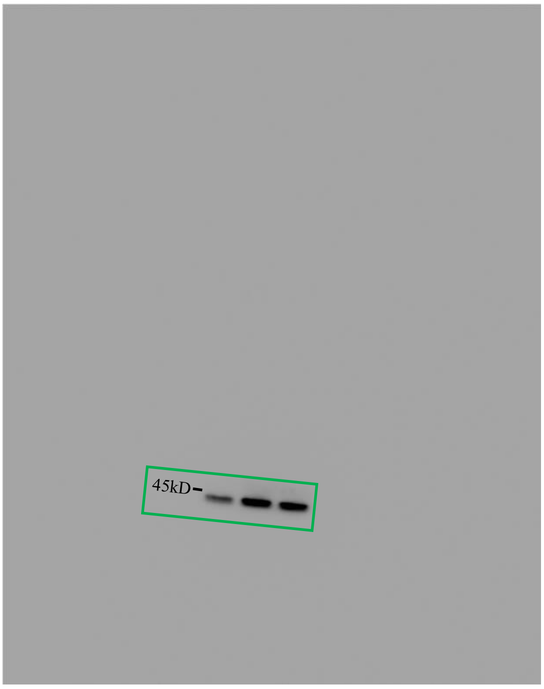

β-actin

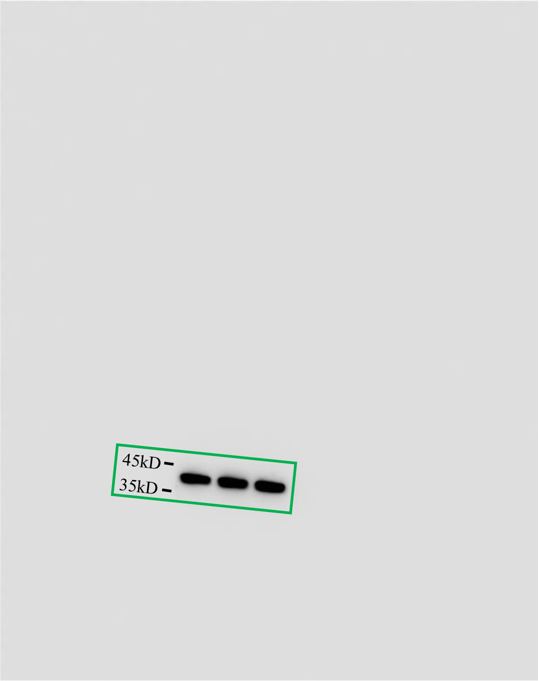

Figure 6A representative image

U251
anti-PAX5(IP)
243aa(IB)

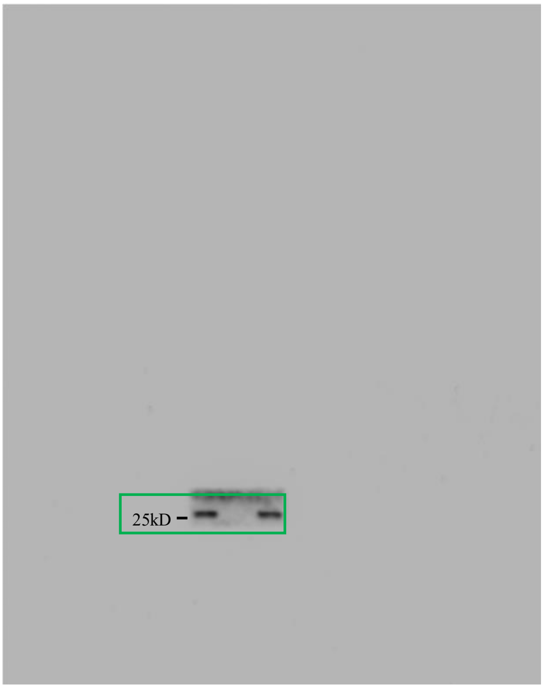

U251
anti-PAX5(IP)
PAX5(IB)

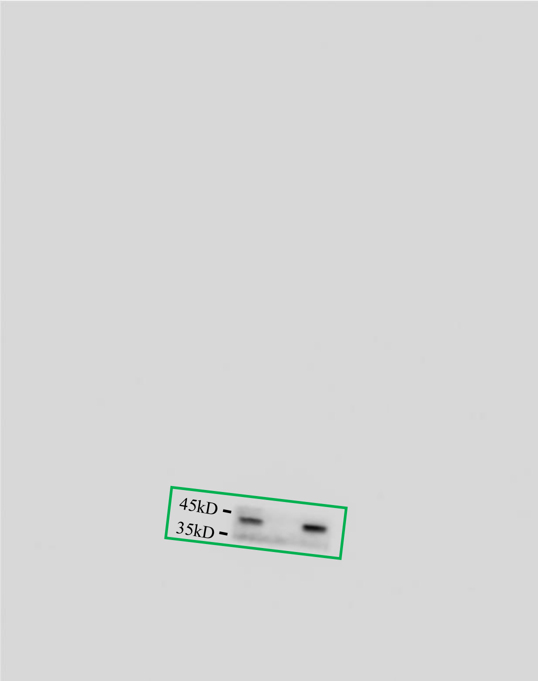

U373
anti-PAX5(IP)
243aa(IB)

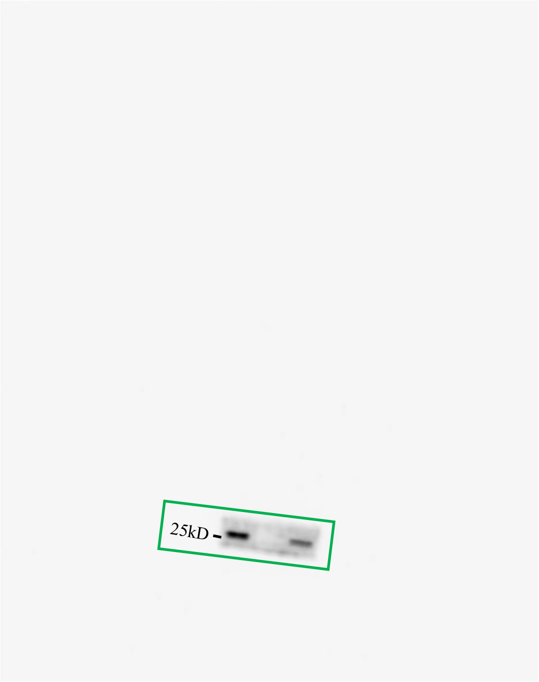

U373
anti-PAX5(IP)
PAX5(IB)

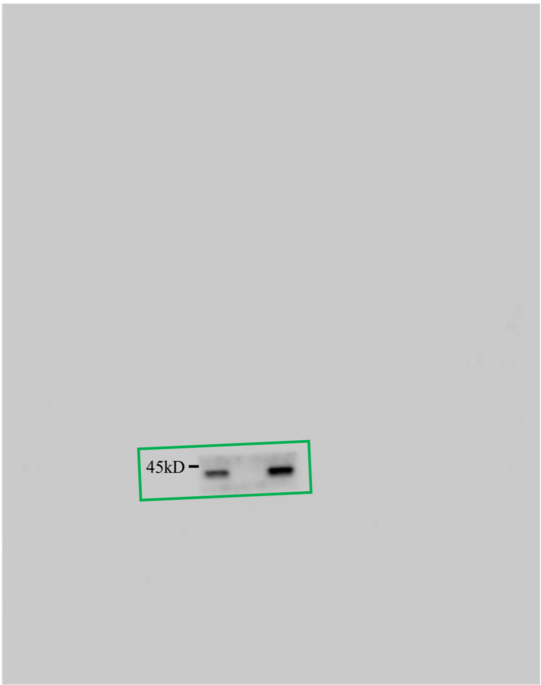

U251
anti-243aa(IP)
PAX5(IB)

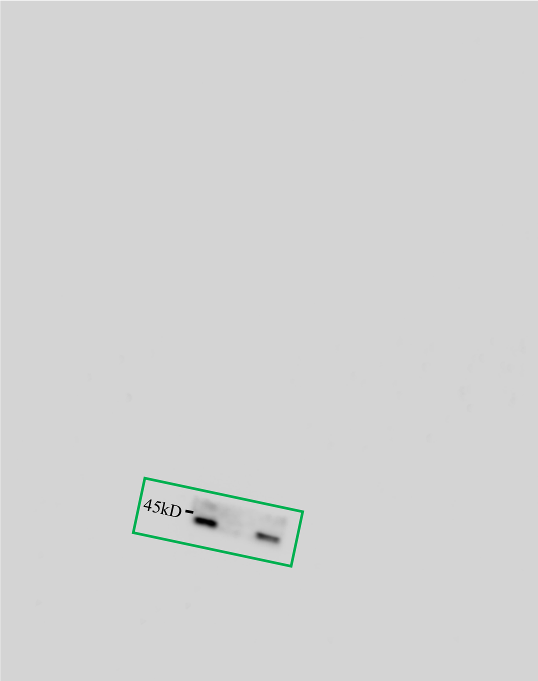

U251
anti-243aa(IP)
243aa(IB)

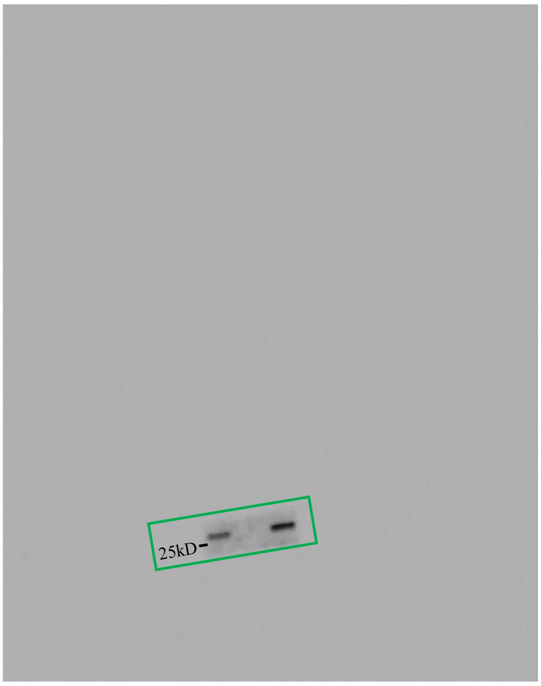

U373
anti-243aa(IP)
PAX5(IB)

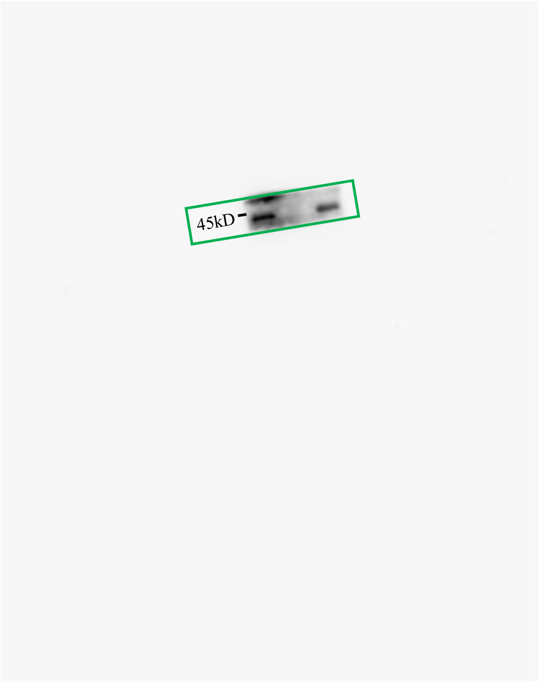

U373
anti-243aa(IP)
243aa(IB)

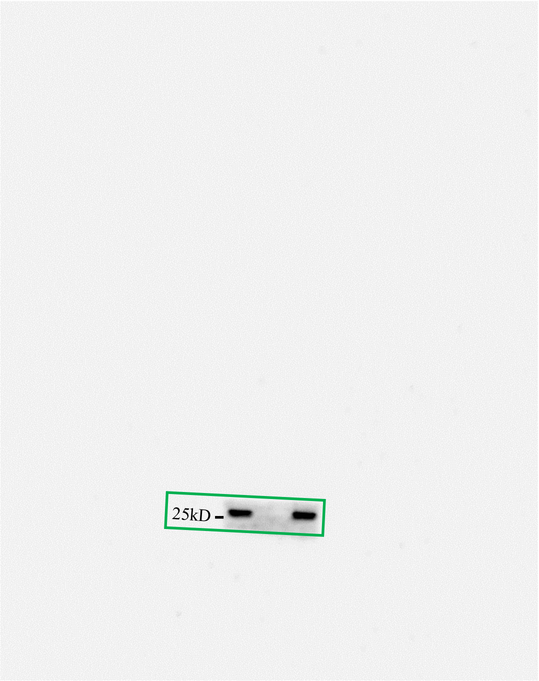

Figure 6H NTRK2-243aa(+) – representative image

PAX5

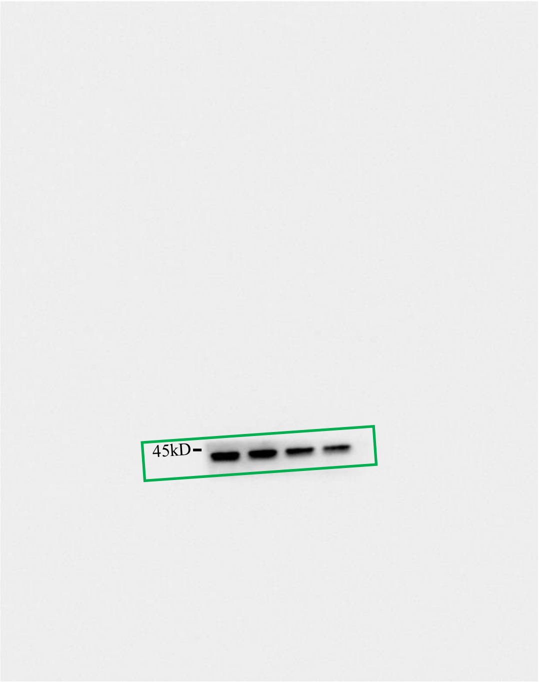

β-actin

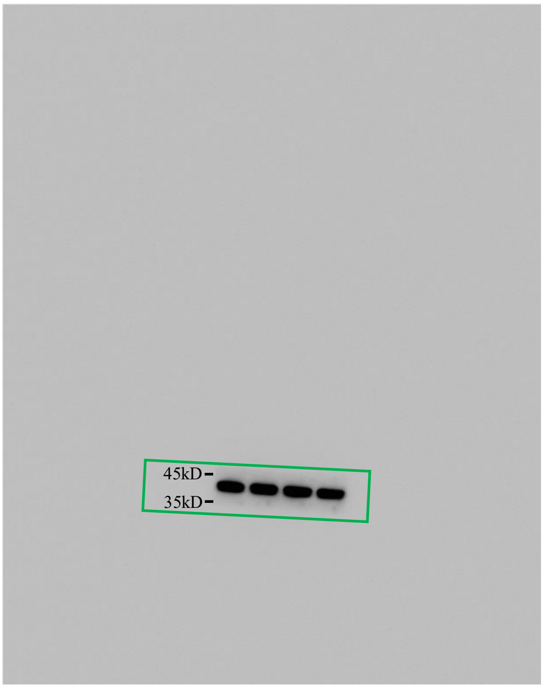

Figure 6H NTRK2-243aa(+) + representative image

PAX5

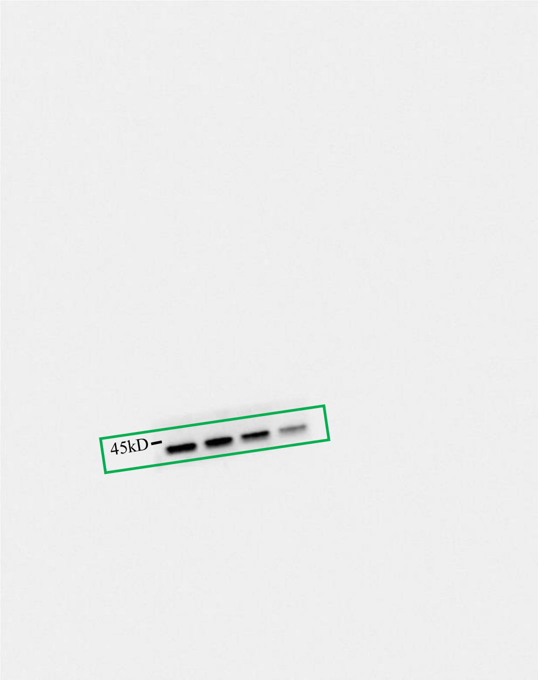

β-actin

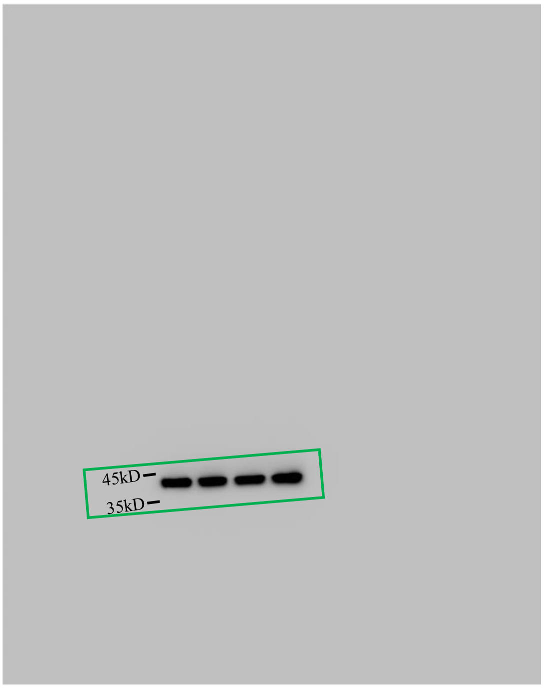

Figure 6I U251 representative image

PAX5

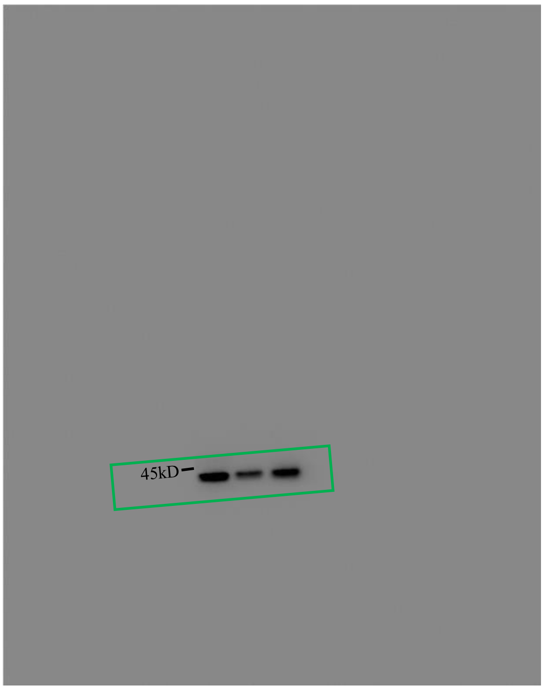

β-actin

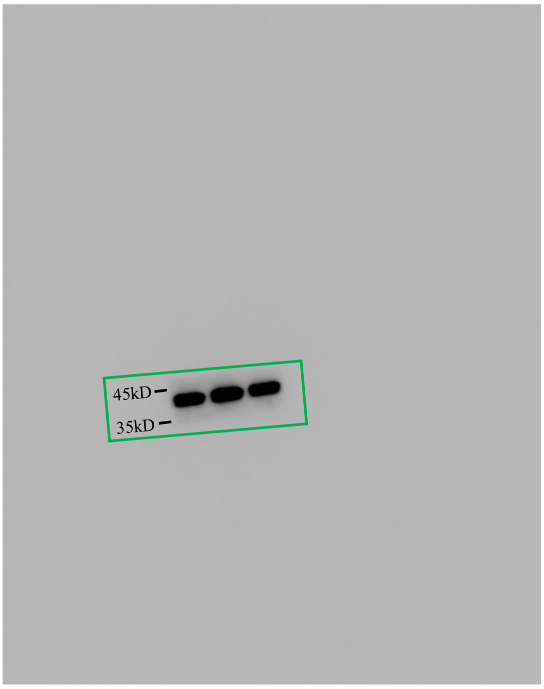

Figure 6I U373 representative image

PAX5

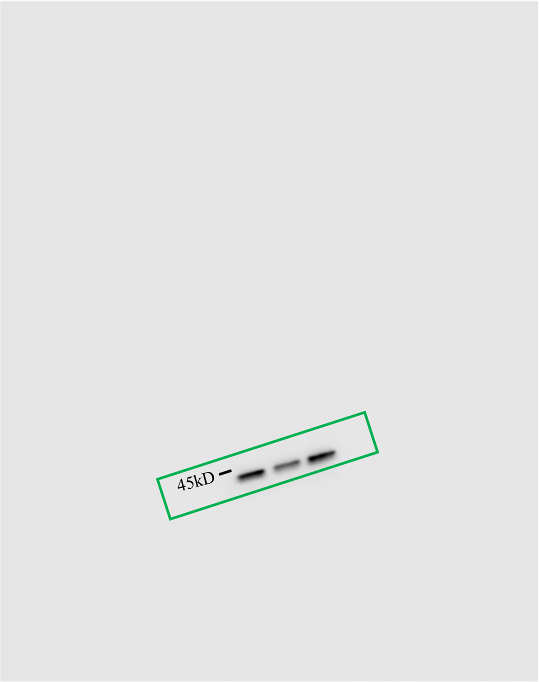

β-actin

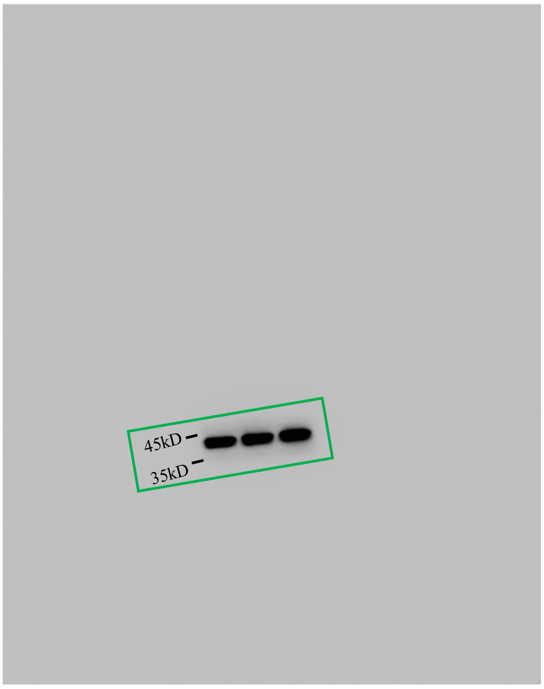

Figure 6J representative image

PAX5 (IP)
Ubiquitin (IB)

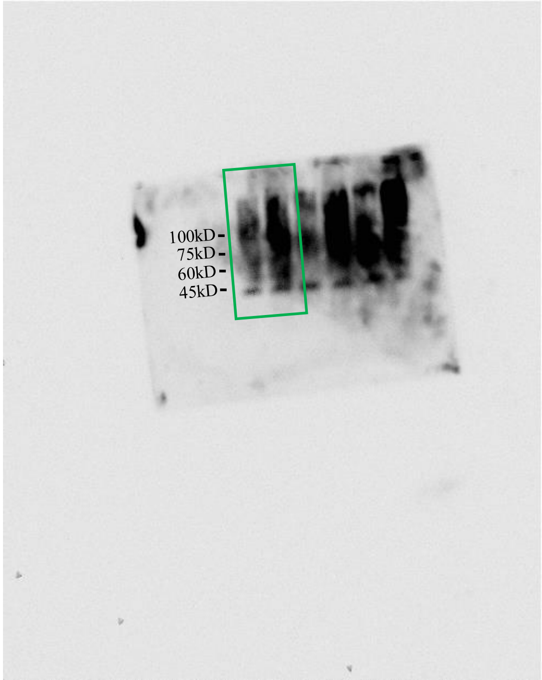

PAX5 (IP)
PAX5 (IB)

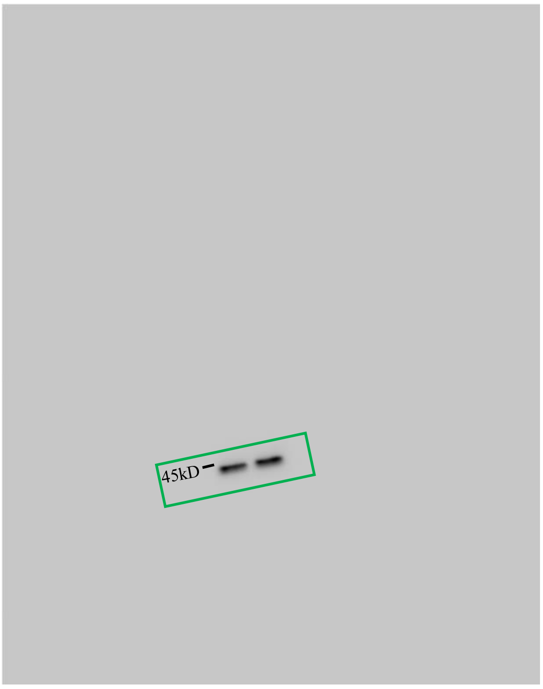

WCE PAX5

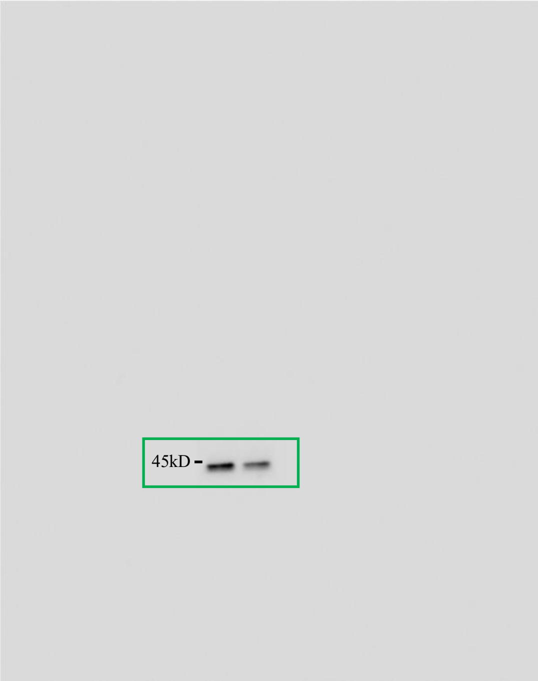

WCE β-actin

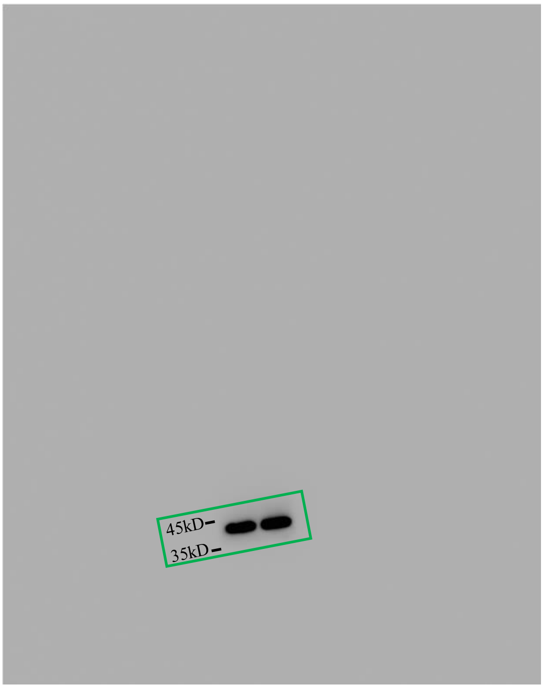

Supplementary Figure 1G U251 representative image

HK2

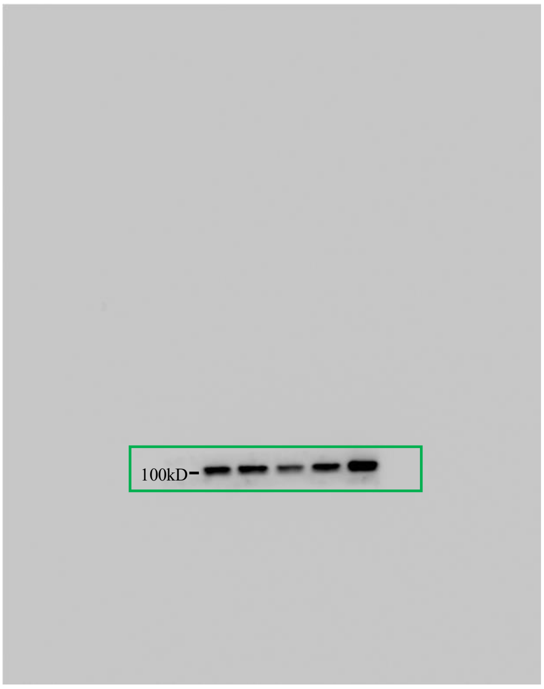

PKM2

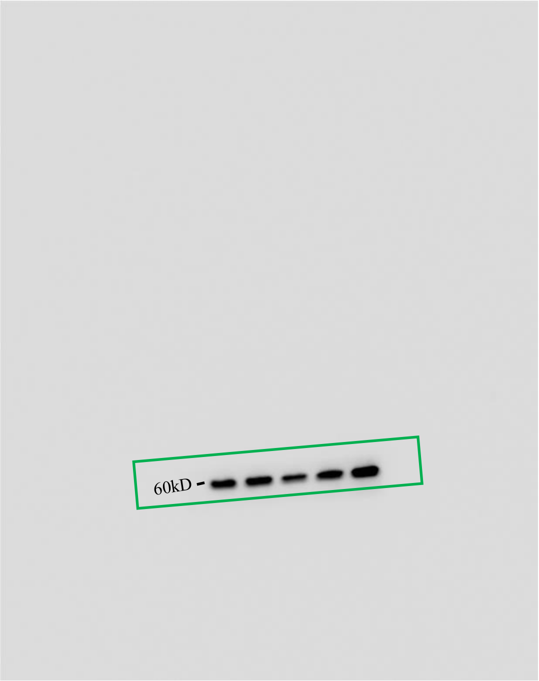

β-actin

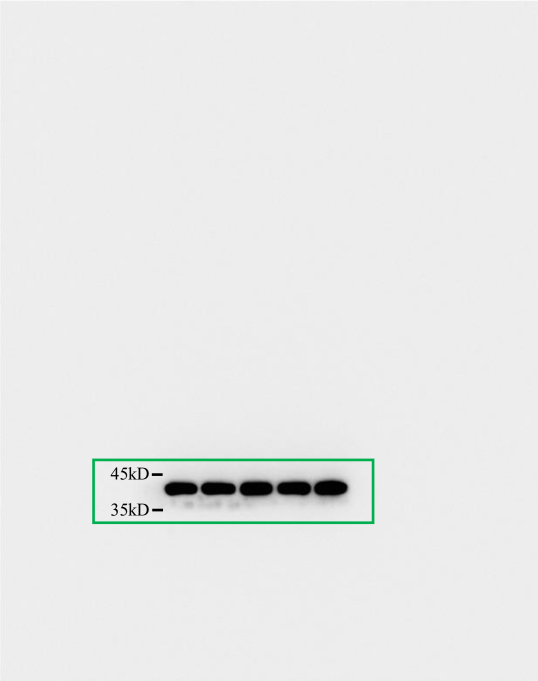

Supplementary Figure 1G U373 representative image

HK2

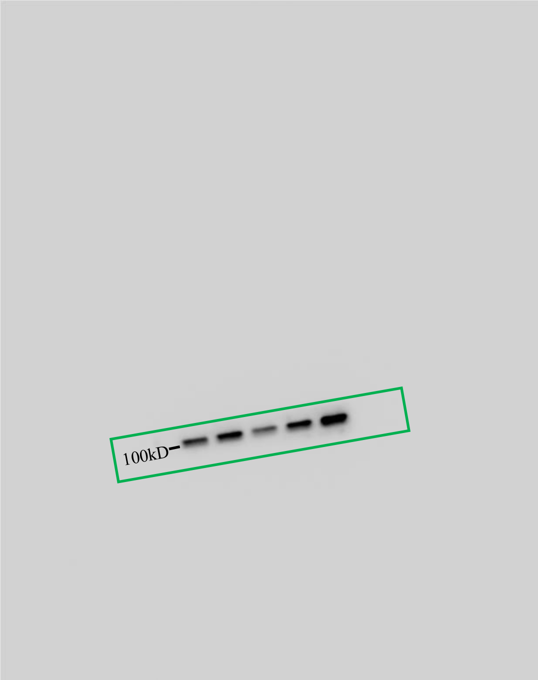

PKM2

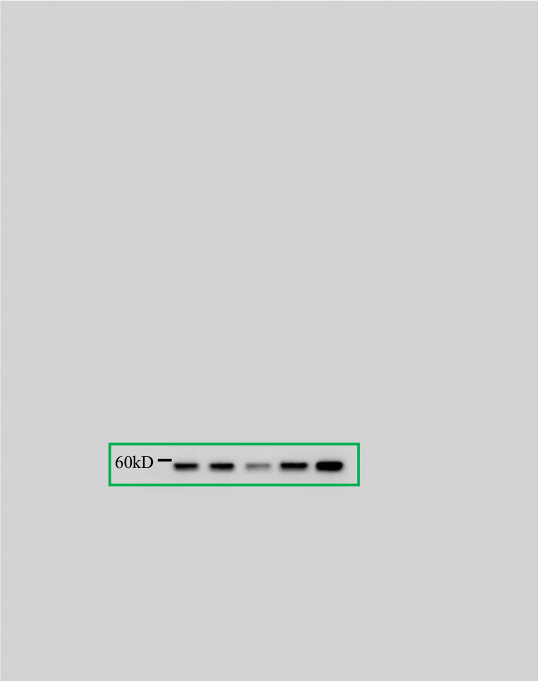

β-actin

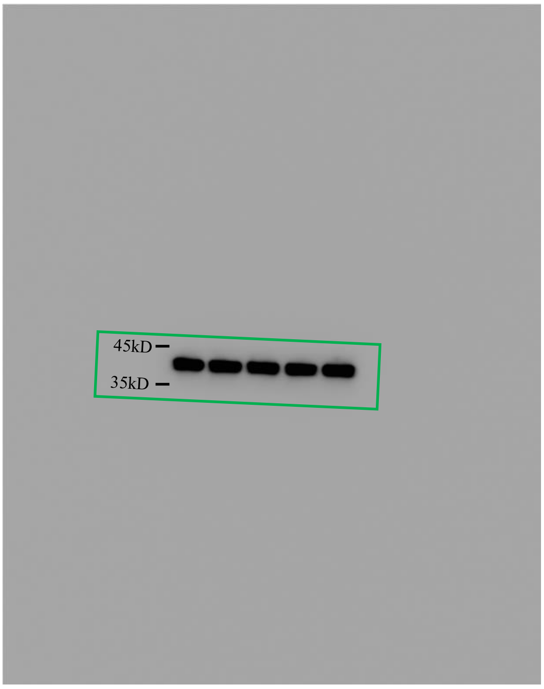

Supplementary Figure 2K U251 representative image

HK2

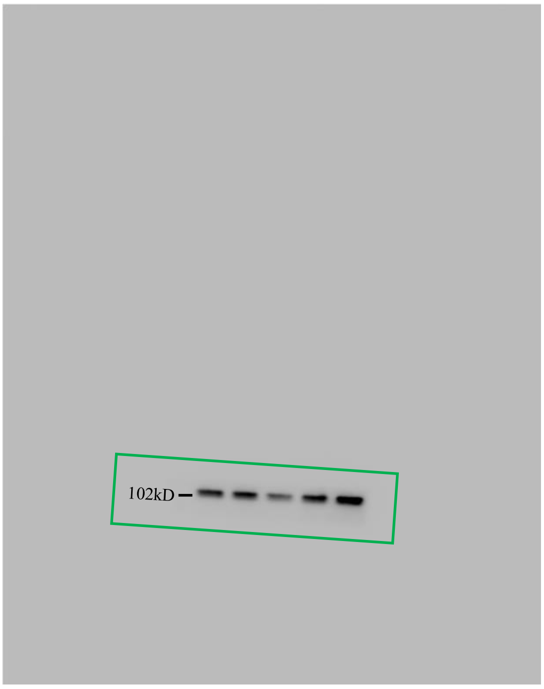

PKM2

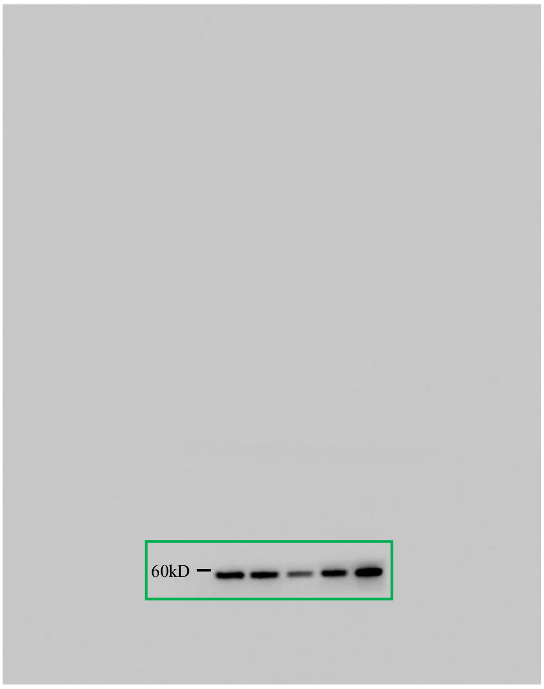

β-actin

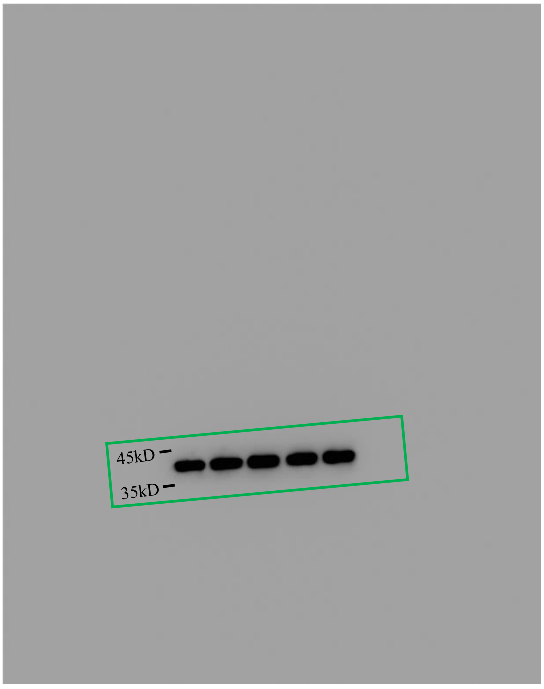

Supplementary Figure 2K U373 representative image

HK2

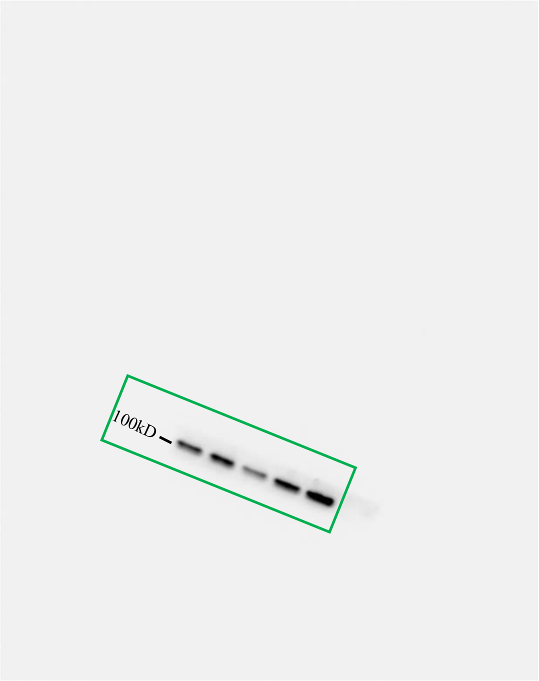

PKM2

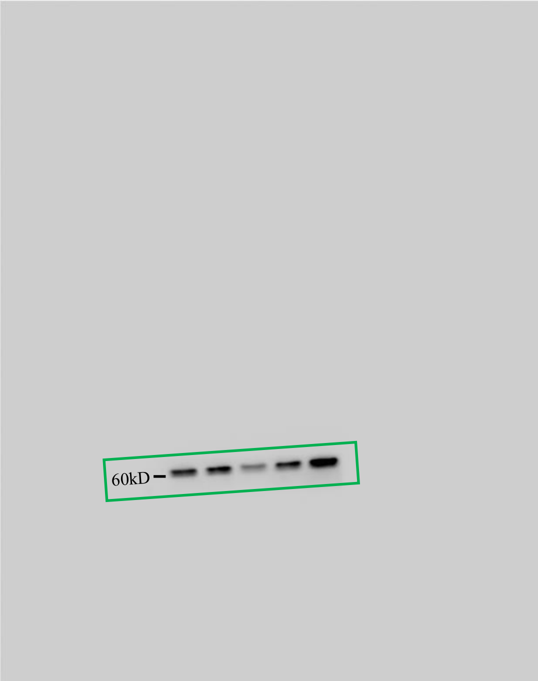

β-actin

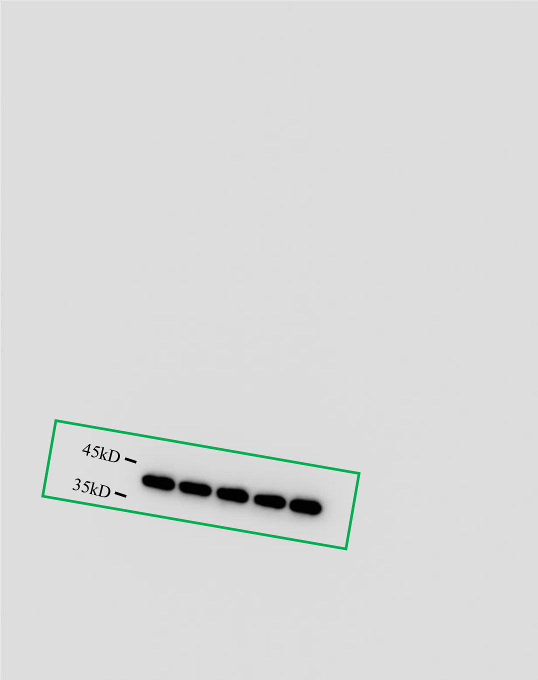

Supplementary Figure 3E U251 representative image

HK2

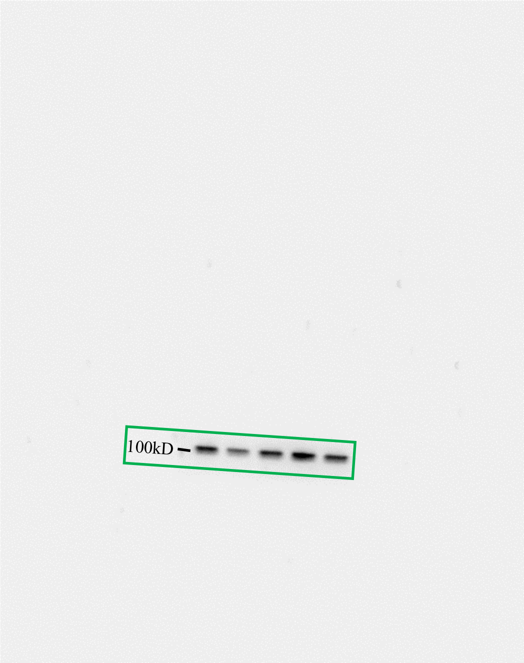

PKM2

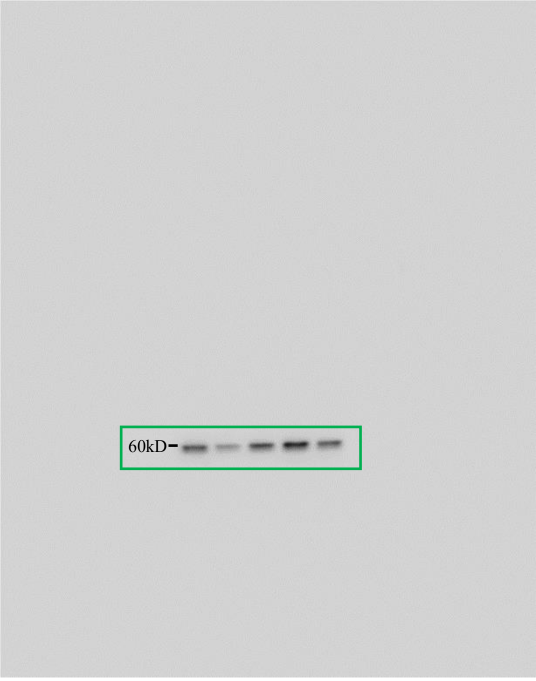

β-actin

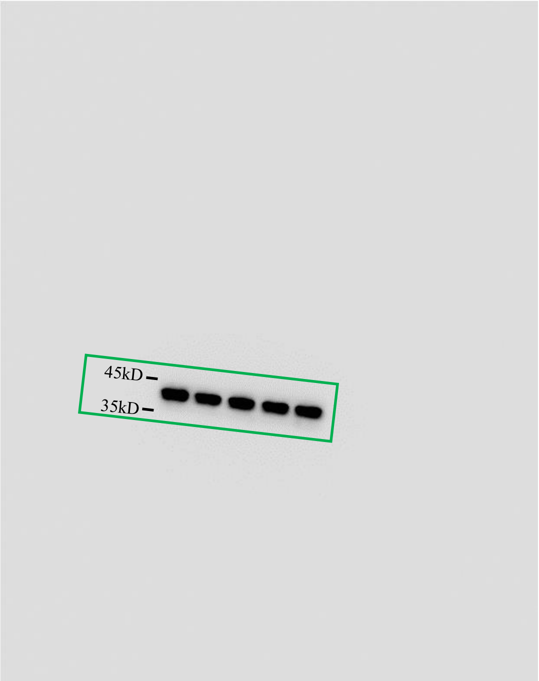

Supplementary Figure 3E U373 representative image

HK2

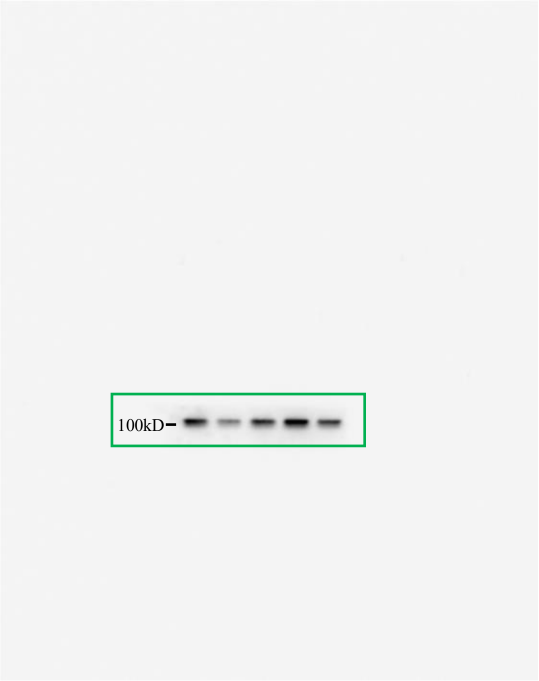

PKM2

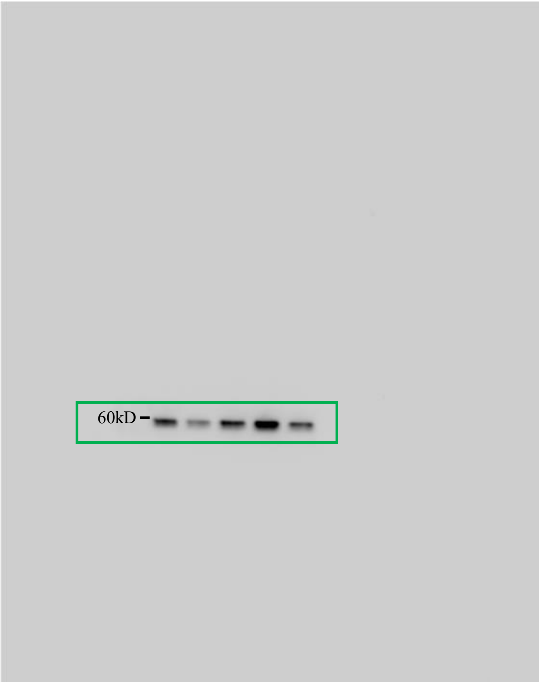

β-actin

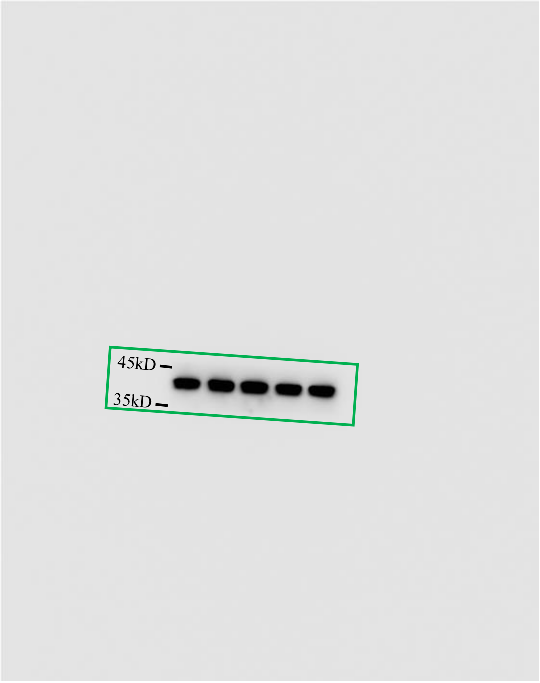

Supplementary Figure 4F representative image

NTRK2-243aa

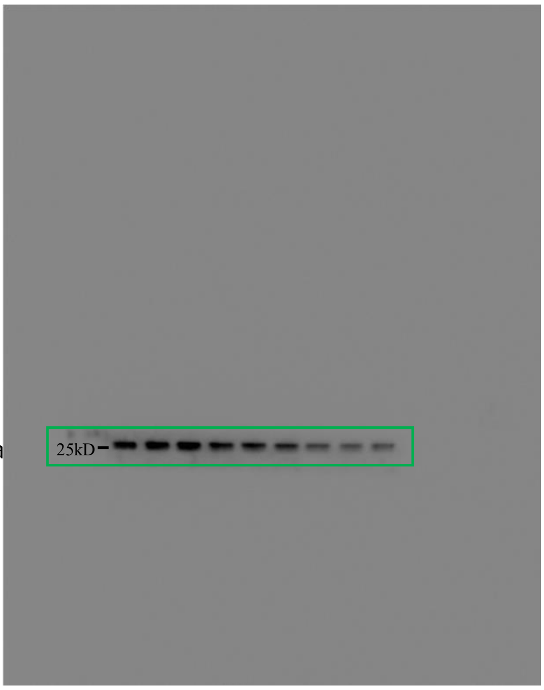

β-actin

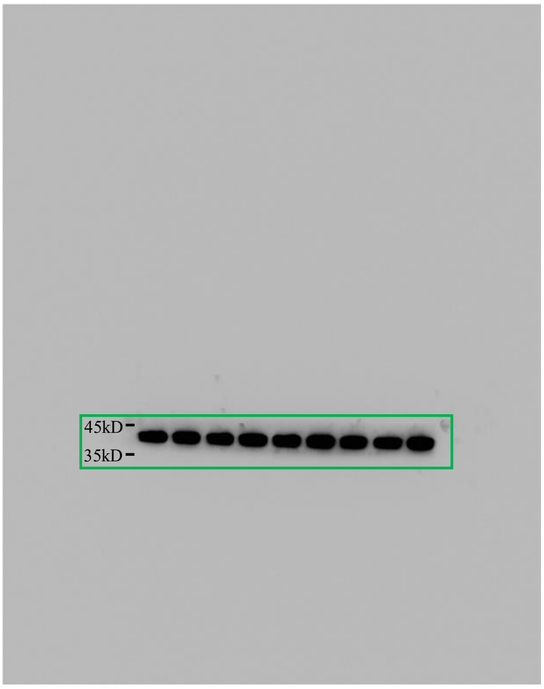

Supplementary Figure 4I U251 representative image

HK2

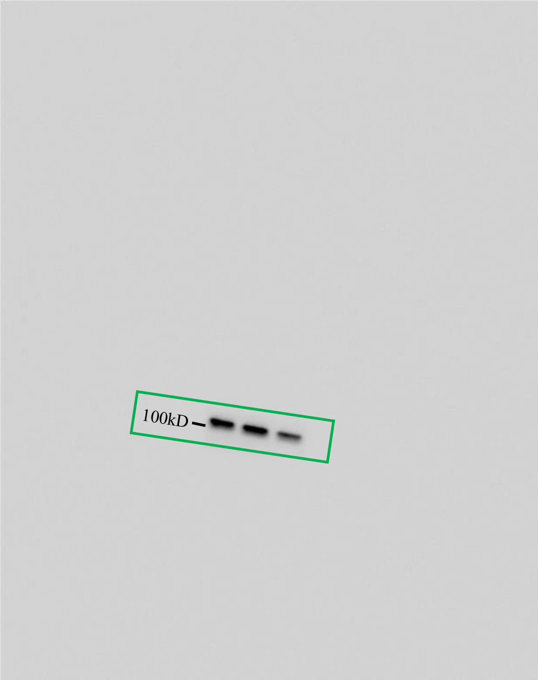

PKM2

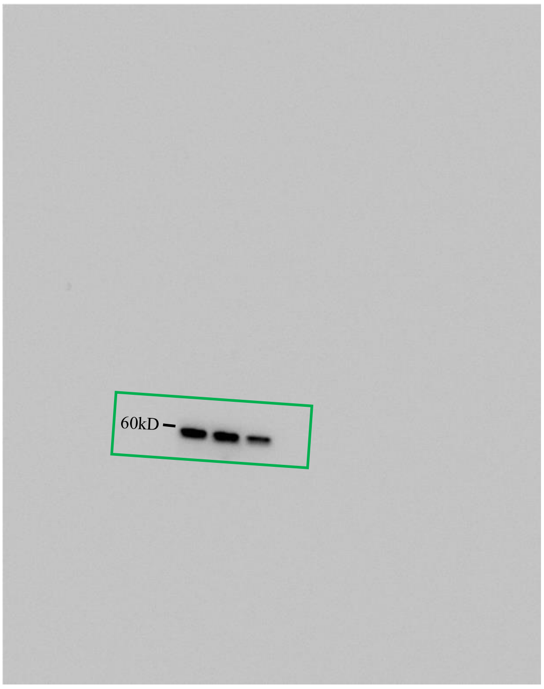

β-actin

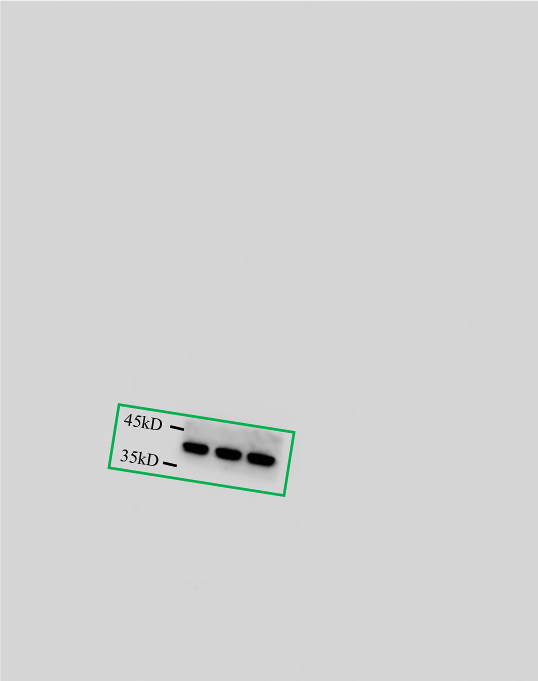

Supplementary Figure 4I U373 representative image

HK2

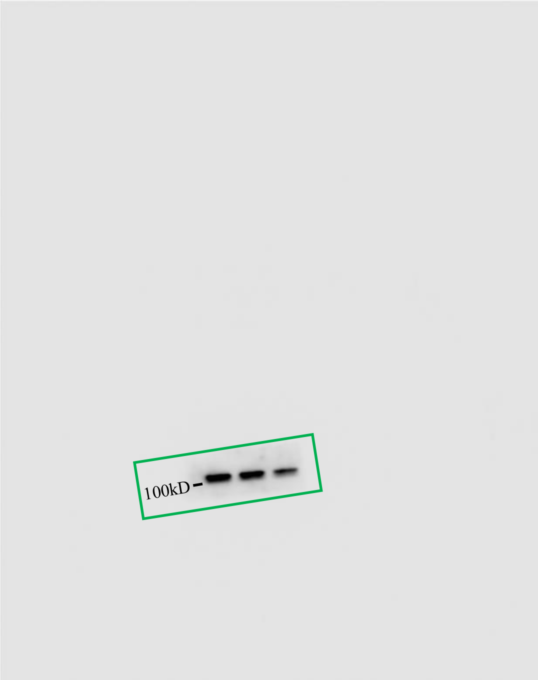

PKM2

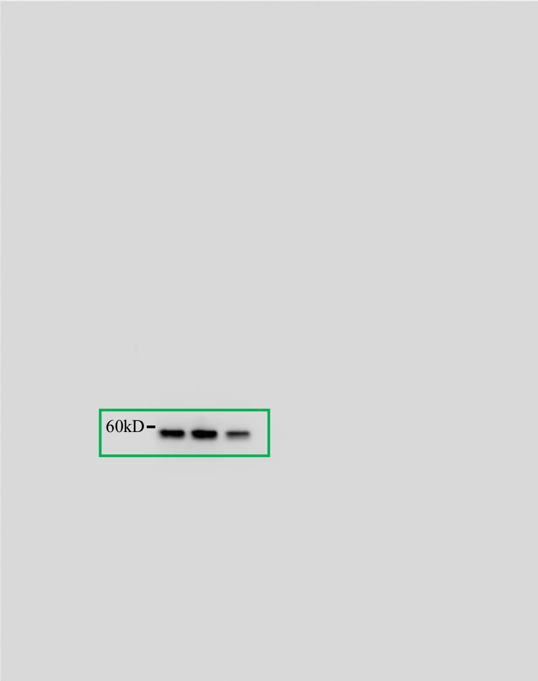

β-actin

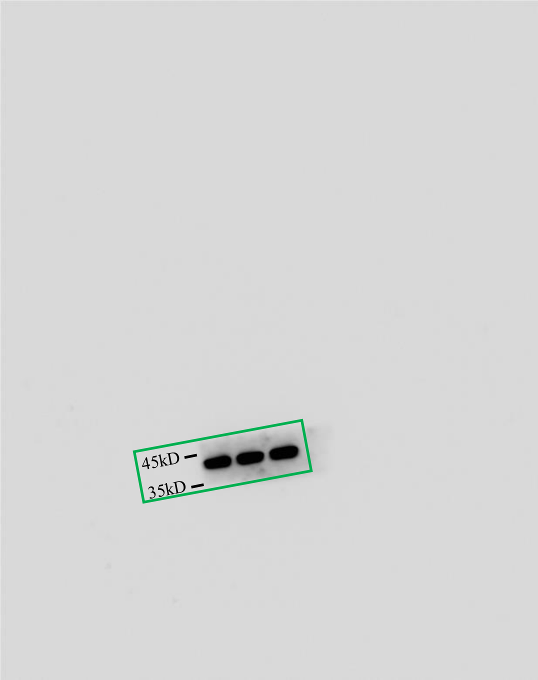

Supplementary Figure 5D left panel representative image

NFAT5

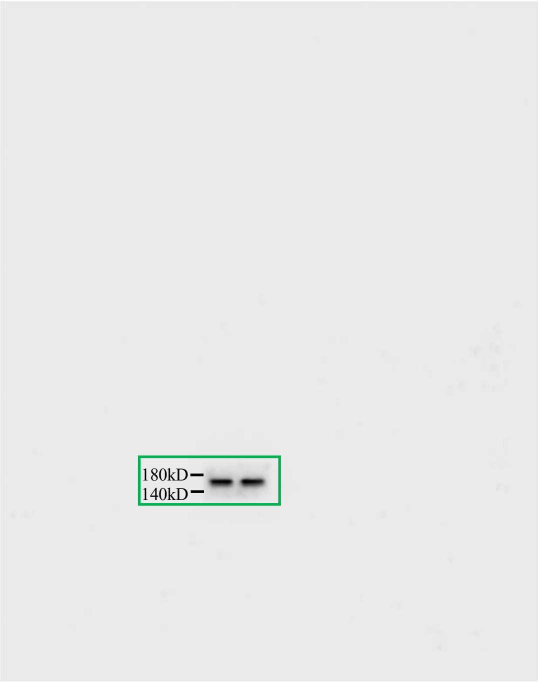

BACH1

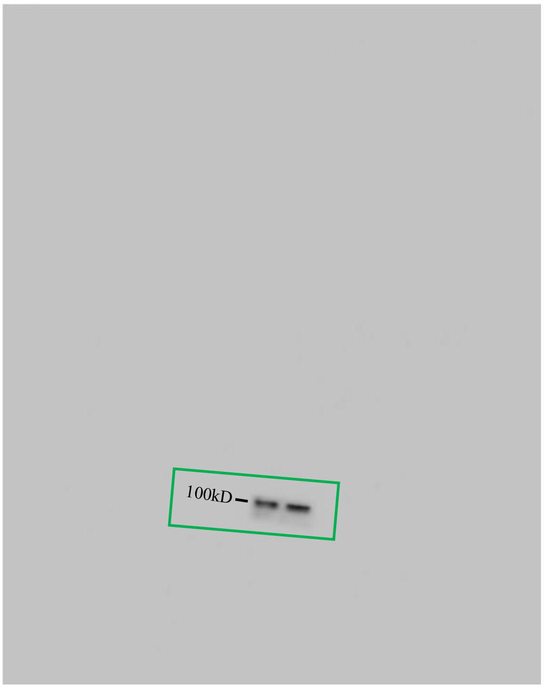

CEBPB

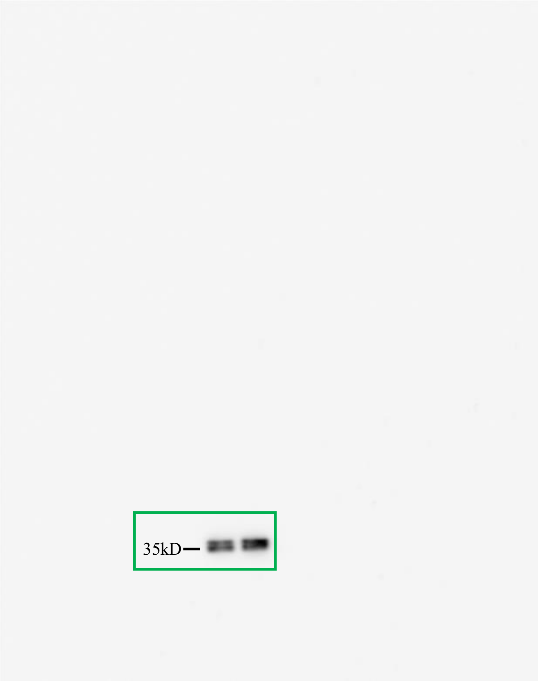

β-actin

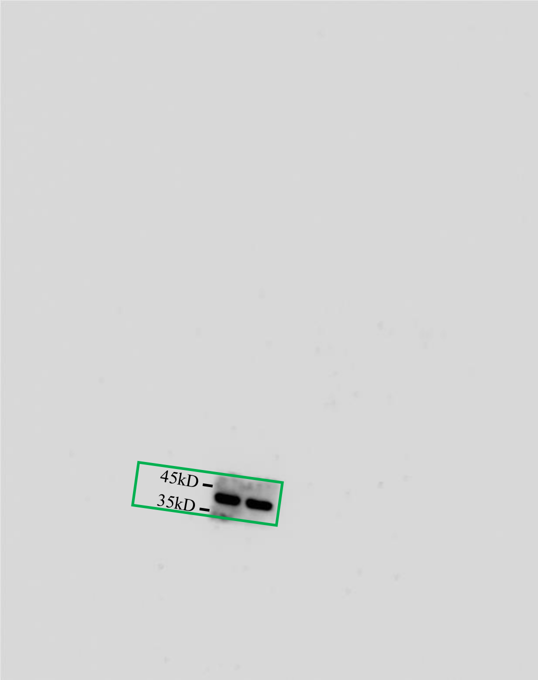

Supplementary Figure 5D right panel representative image

FOXP3

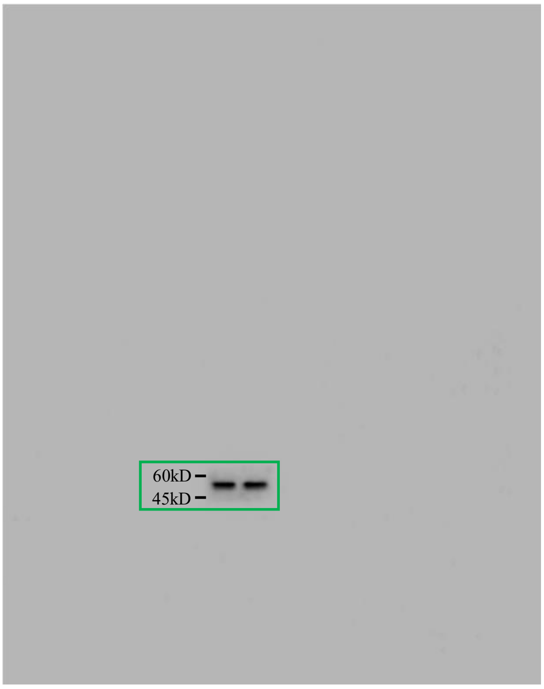

PAX5

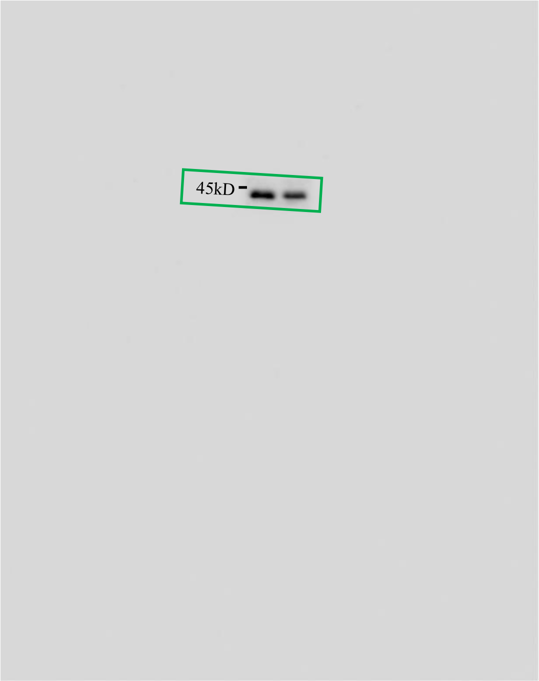

KLF14

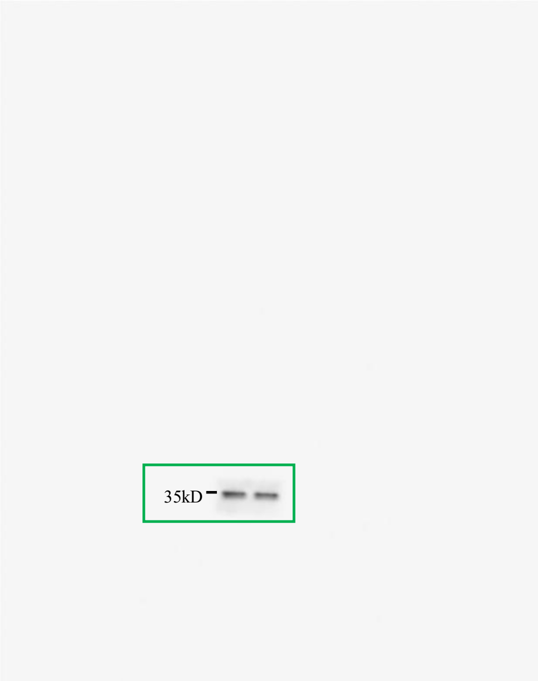

β-actin

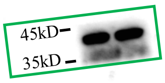

Supplementary Figure 5O U251 representative image

HK2

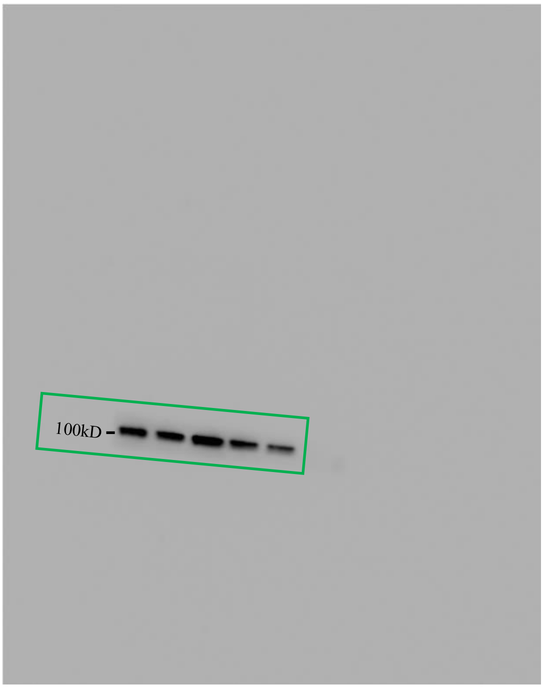

PKM2

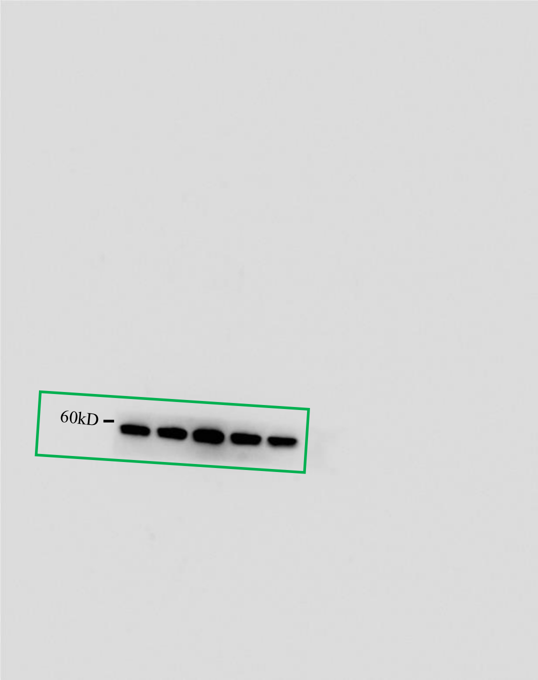

β-actin

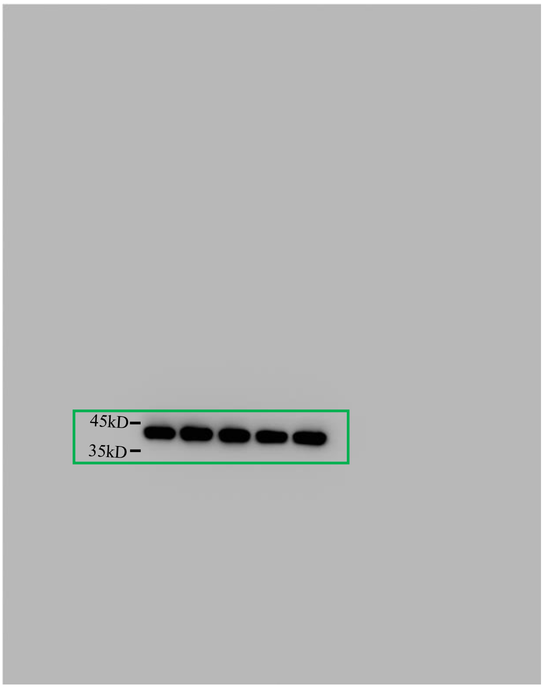

Supplementary Figure 5P U373 representative image

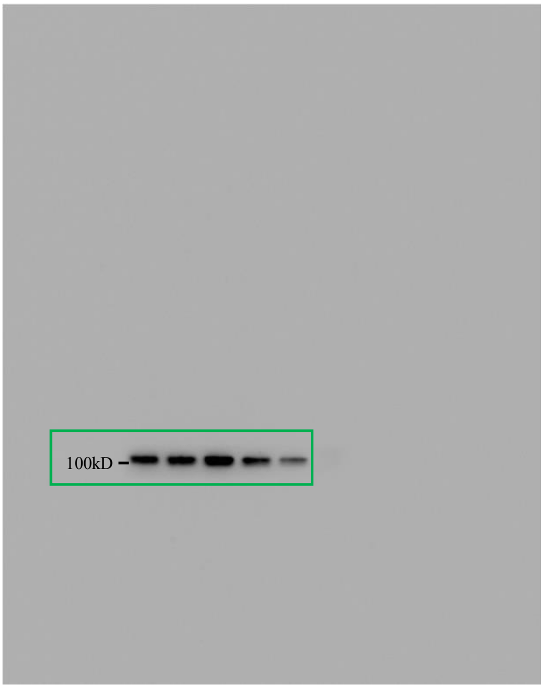

HK2

PKM2

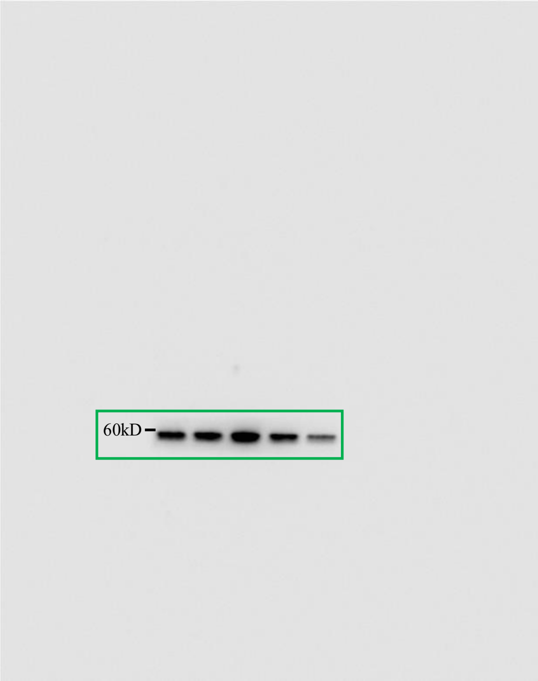

β-actin

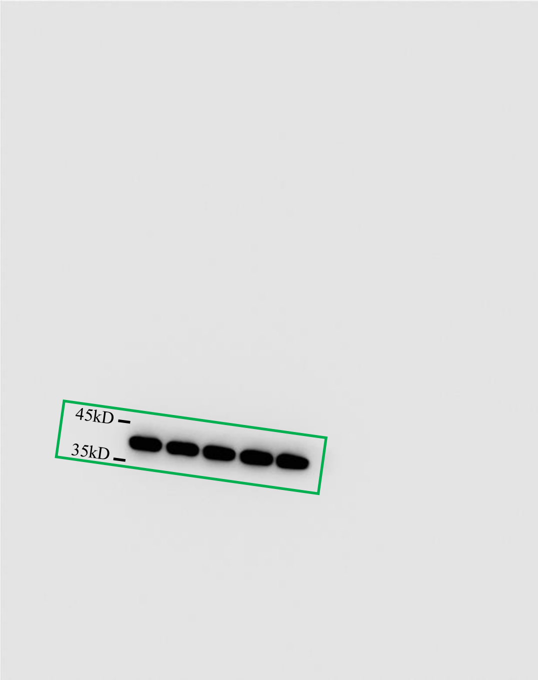

Supplementary Figure 6C U251 representative image

PAX5

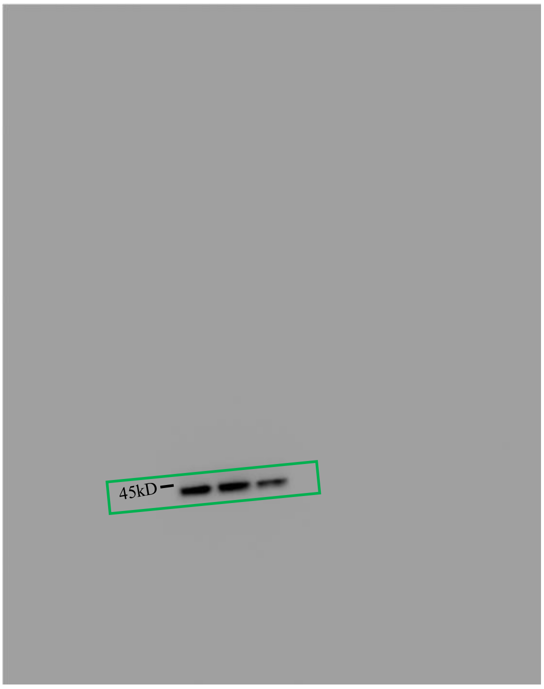

β-actin

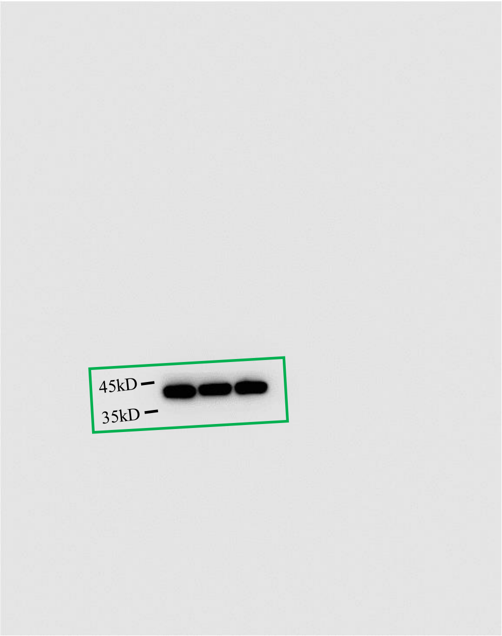

Supplementary Figure 6C U373 representative image

PAX5

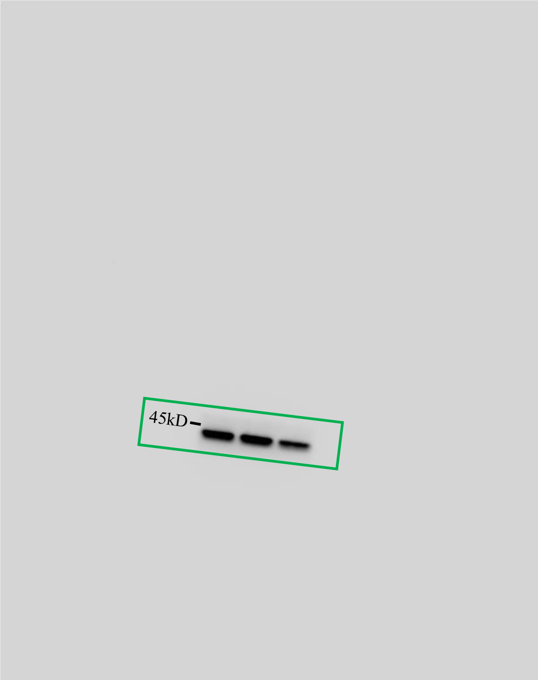

β-actin

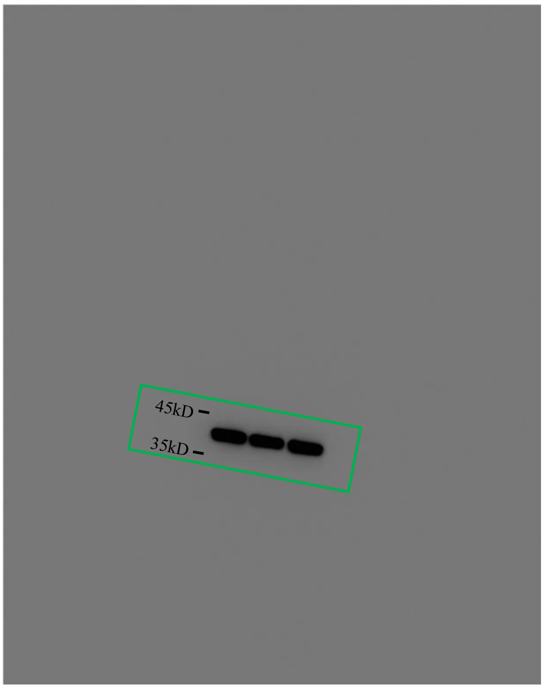

Supplementary Figure 6I NTRK2-243aa (+) - representative image

PAX5

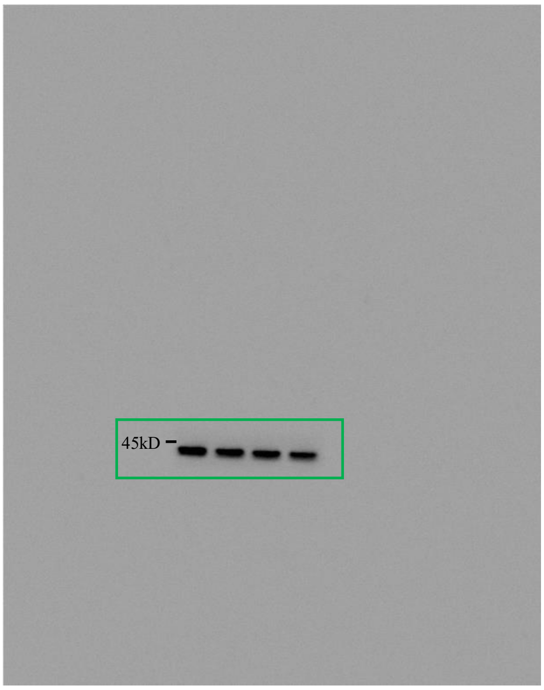

β-actin

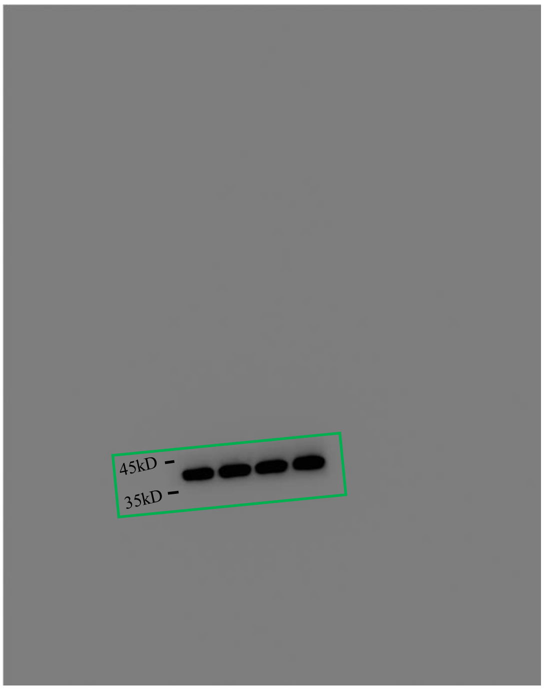

Supplementary Figure 6I NTRK2-243aa (+)+ representative image

PAX5

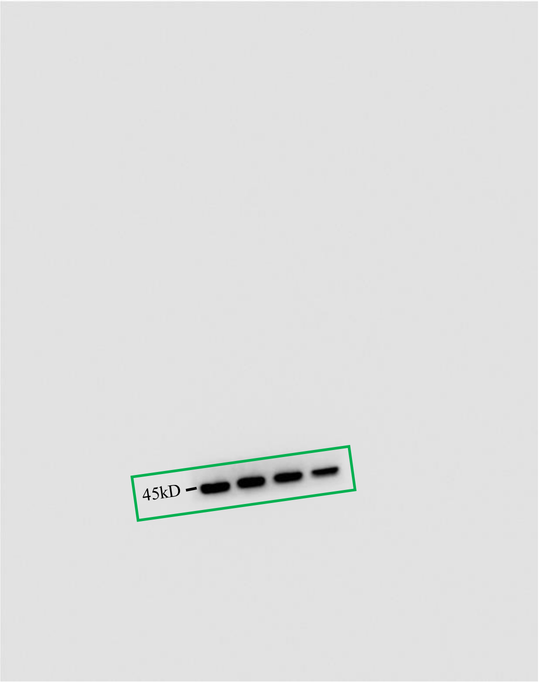

β-actin

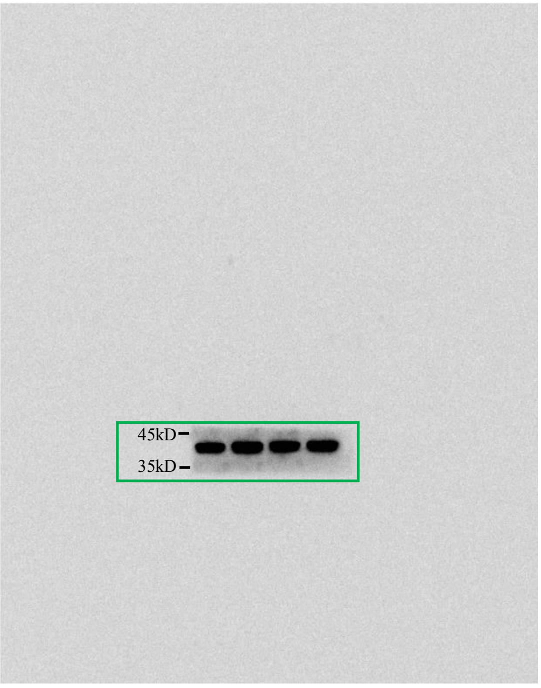

Supplementary Figure 6K U251 representative image

UHRF2

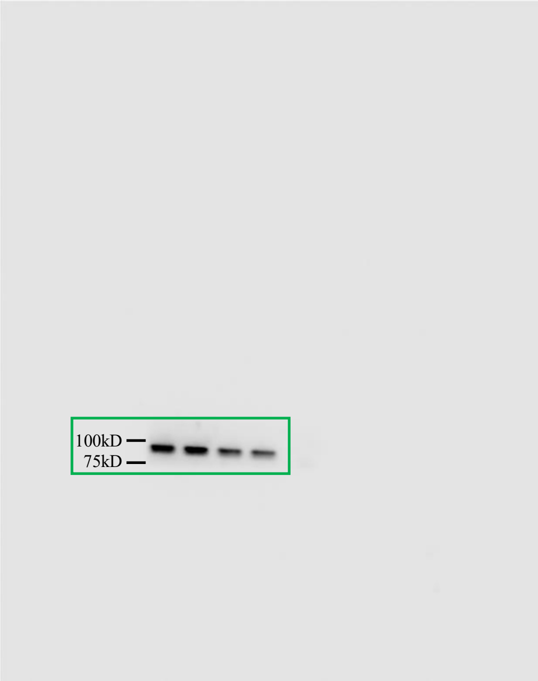

PAX5

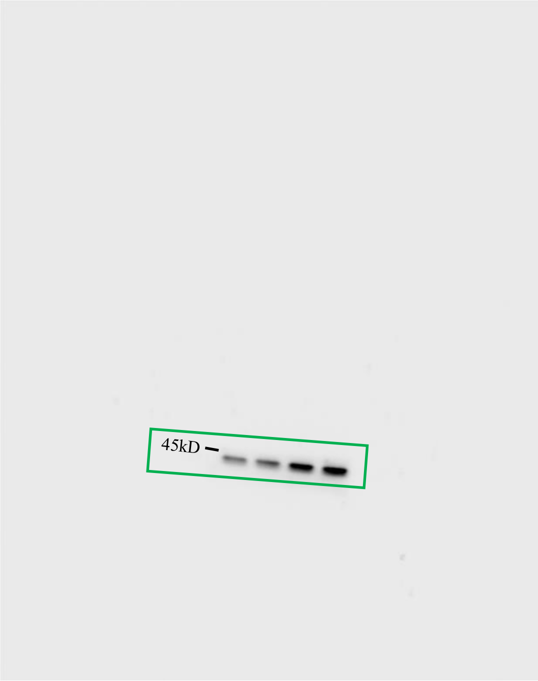

β-actin

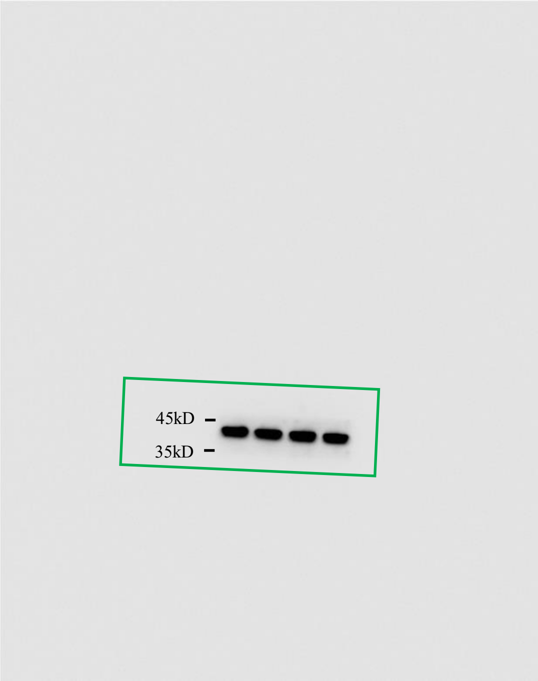

Supplementary Figure 6K U373 representative image

UHRF2

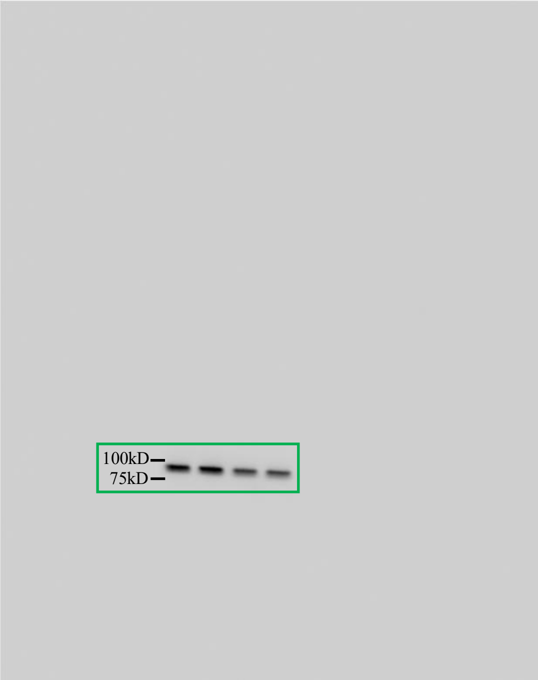

PAX5

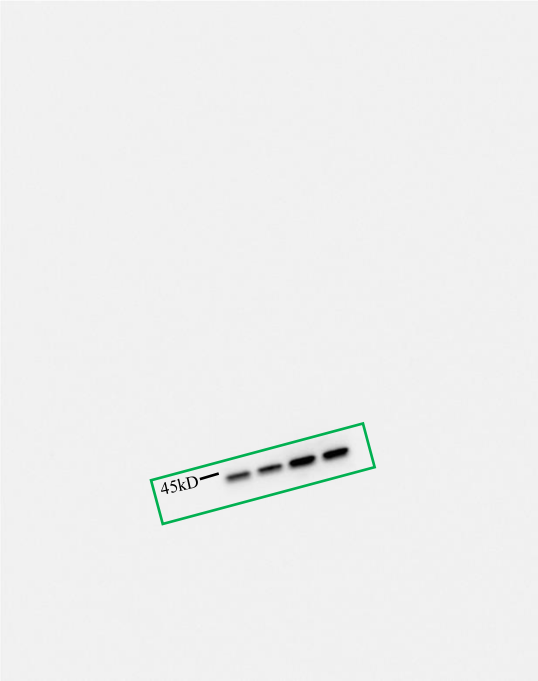

β-actin

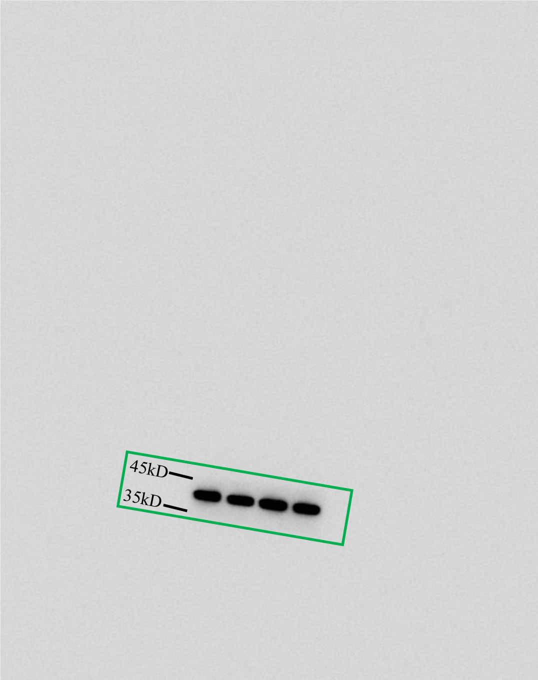

Supplementary Figure 6L representative image

PAX5(IP)
ubiquitin(IB)

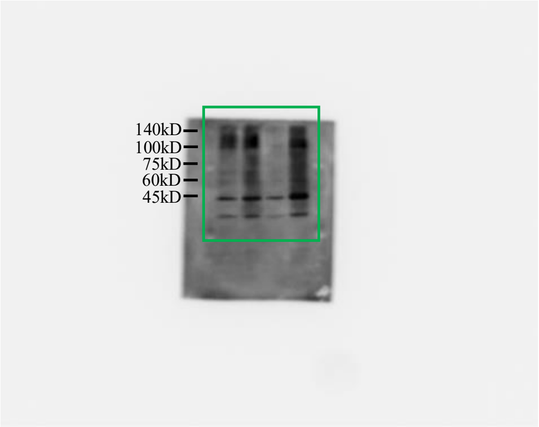

PAX5(IP)
p-PAX5(IB)

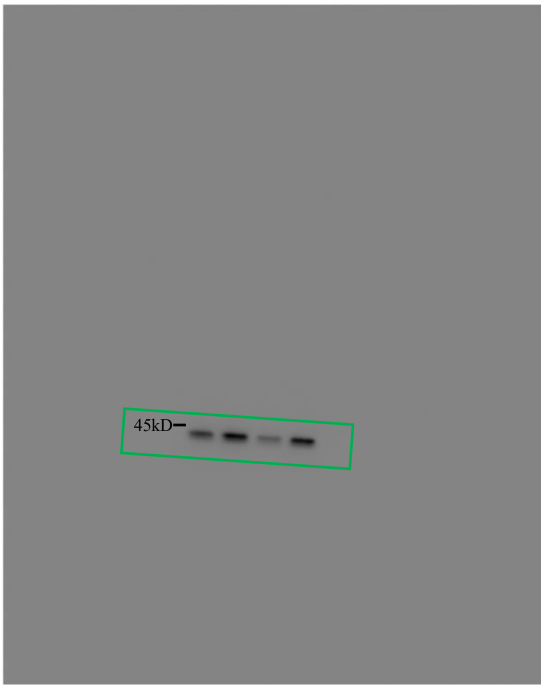

PAX5(IP)
PAX5(IB)

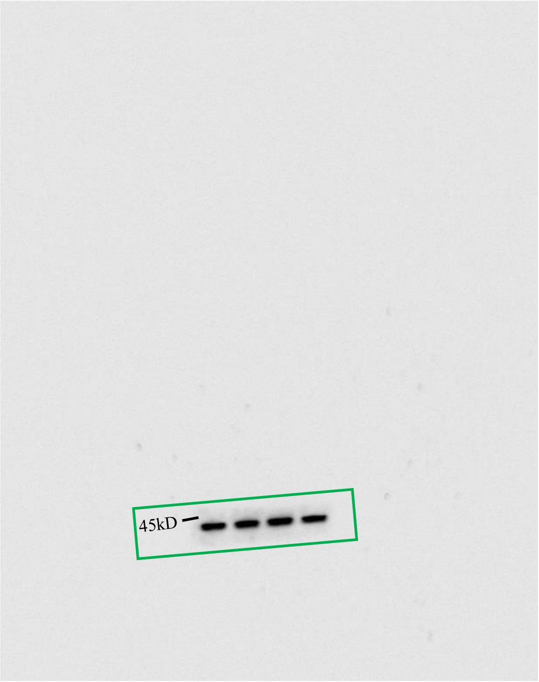

WCE UHRF2

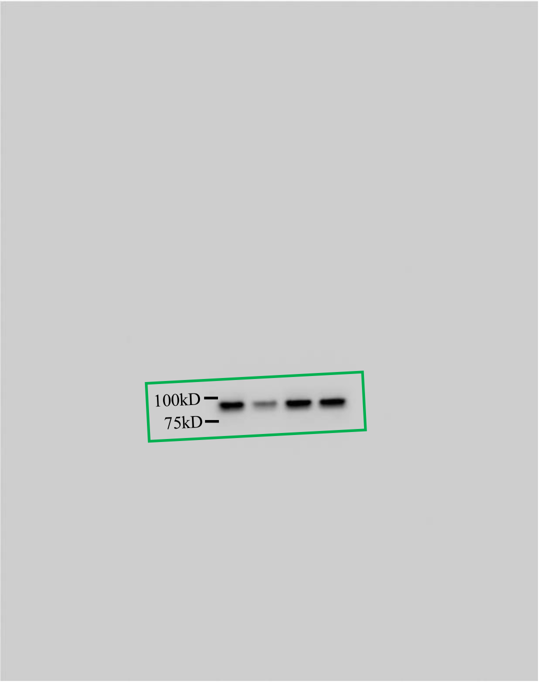

WCE PAX5

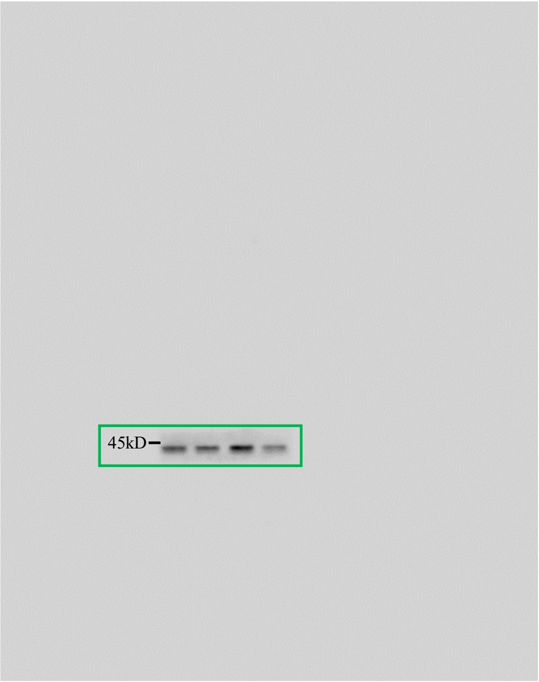

WCE β-actin

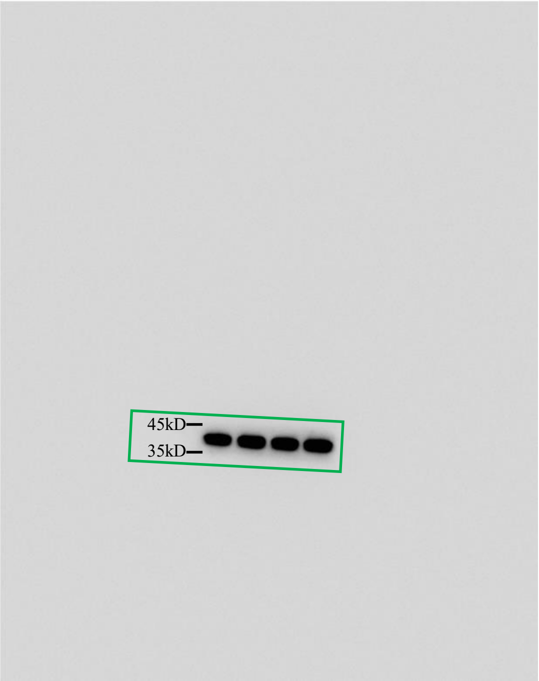

Supplementary Figure 7A U251 representative image

HK2

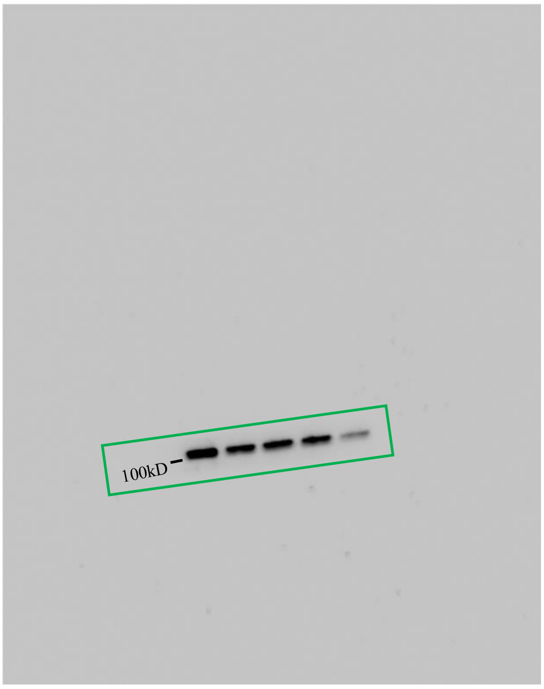

PKM2

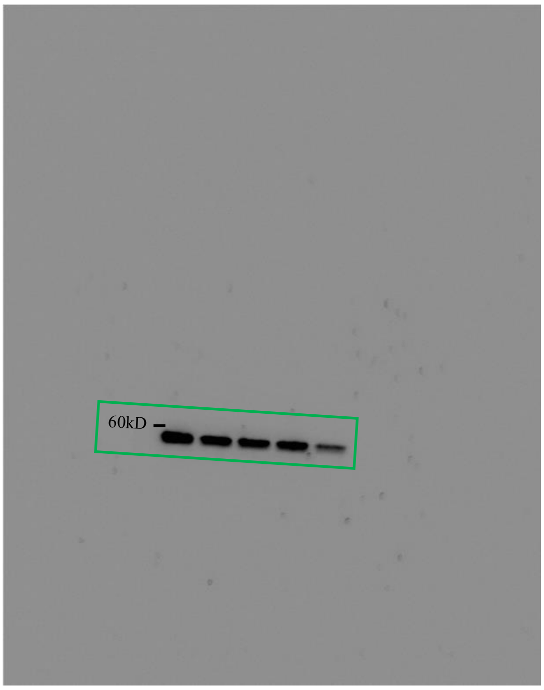

β-actin

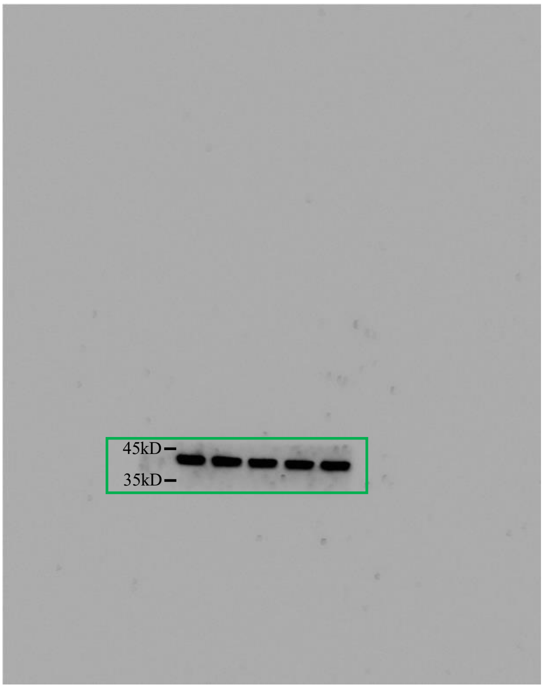

Supplementary Figure 7A U373 representative image

HK2

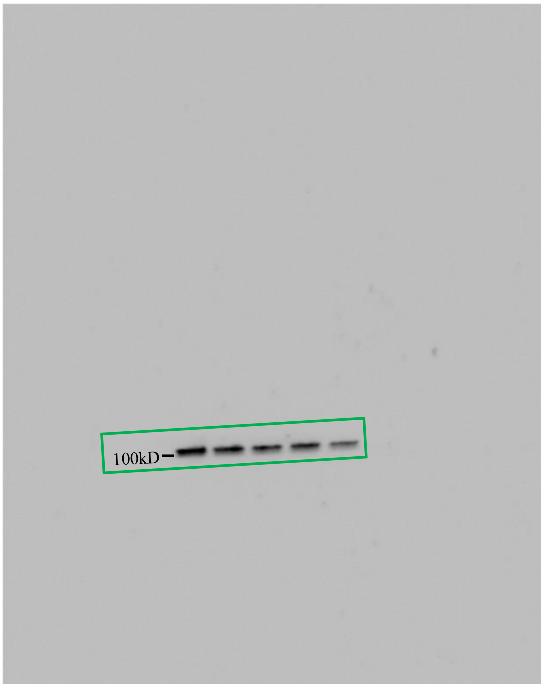

PKM2

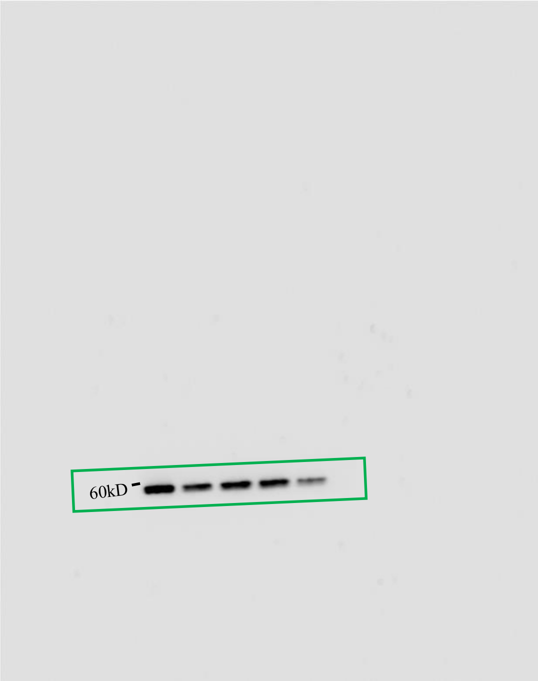

β-actin

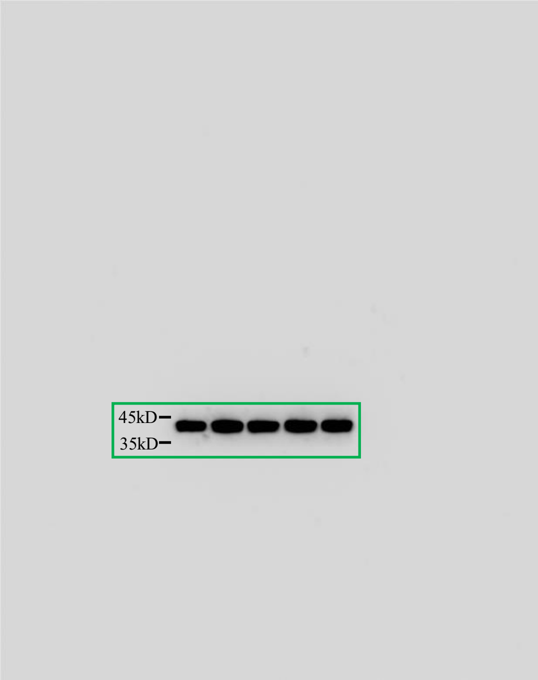

Supplementary Figure 7B U251 representative image

HK2

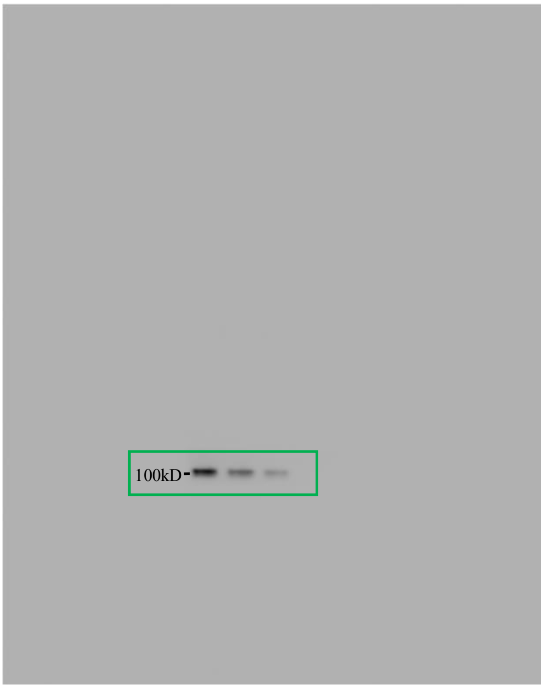

PKM2

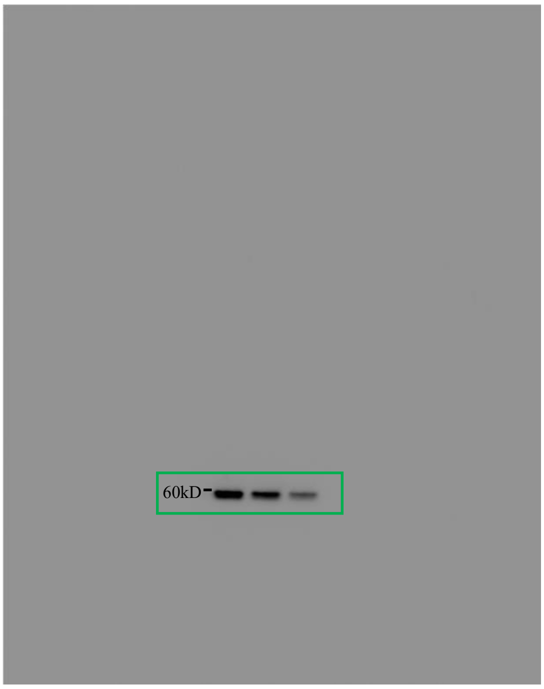

β-actin

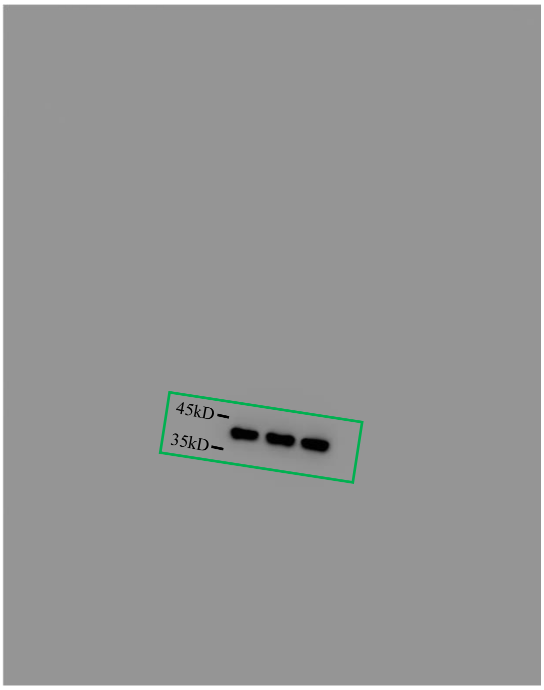

Supplementary Figure 7B U373 representative image

HK2

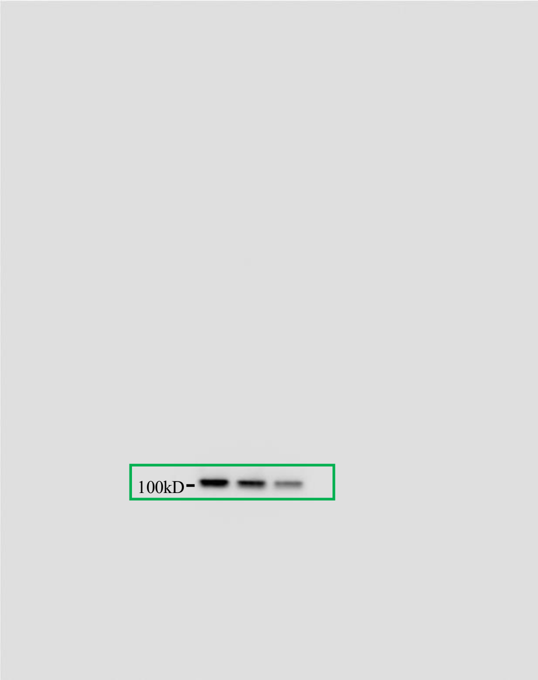

PKM2

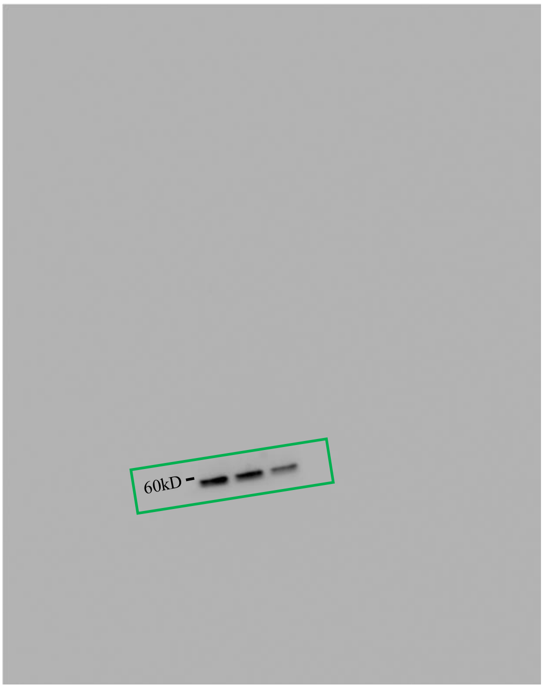

β-actin

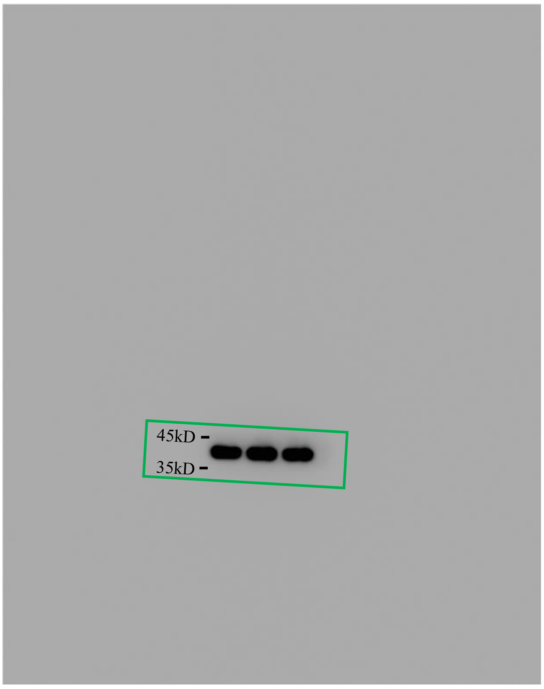

Supplementary Figure 8B U251 representative image

MBNL1

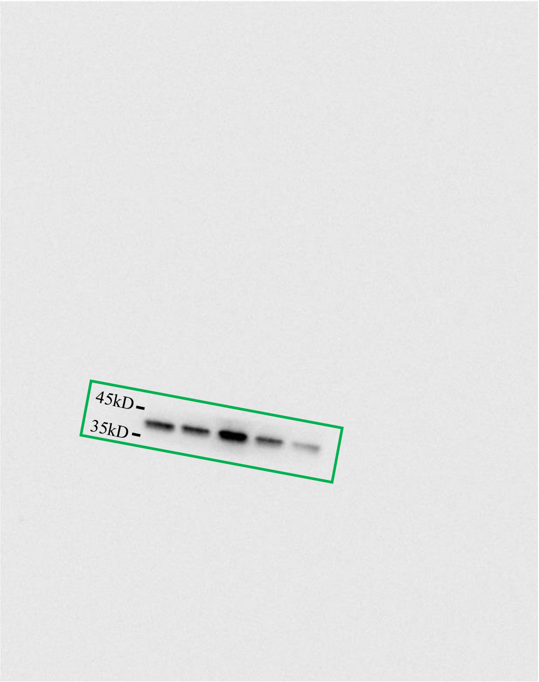

β-actin

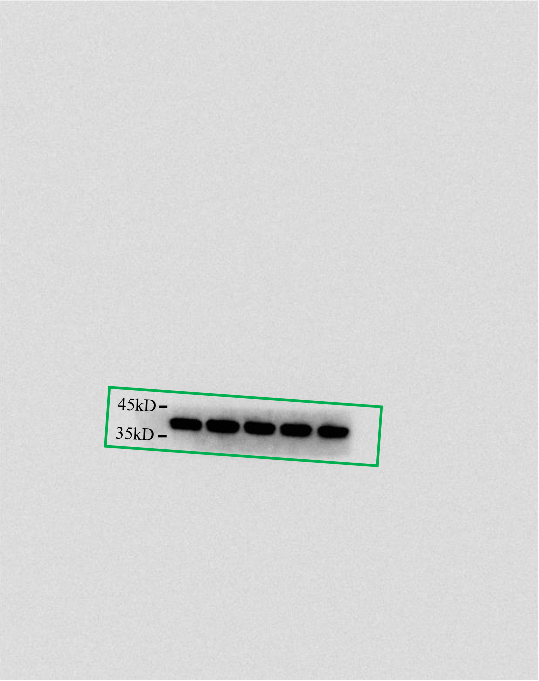

Supplementary Figure 8B U373 representative image

MBNL1

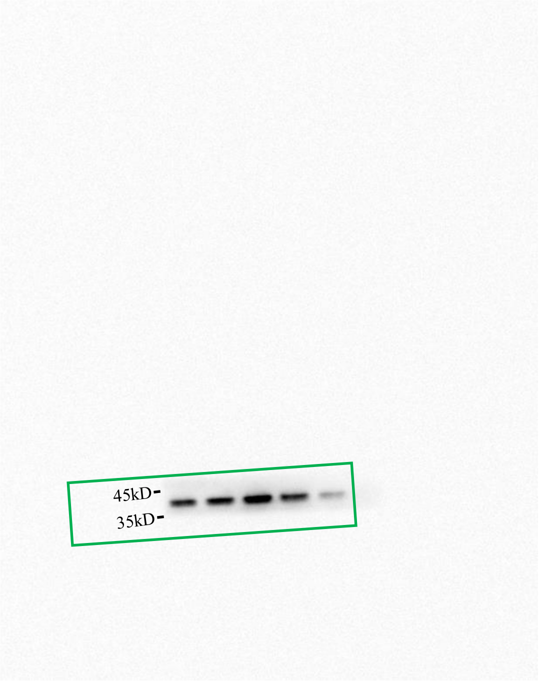

β-actin

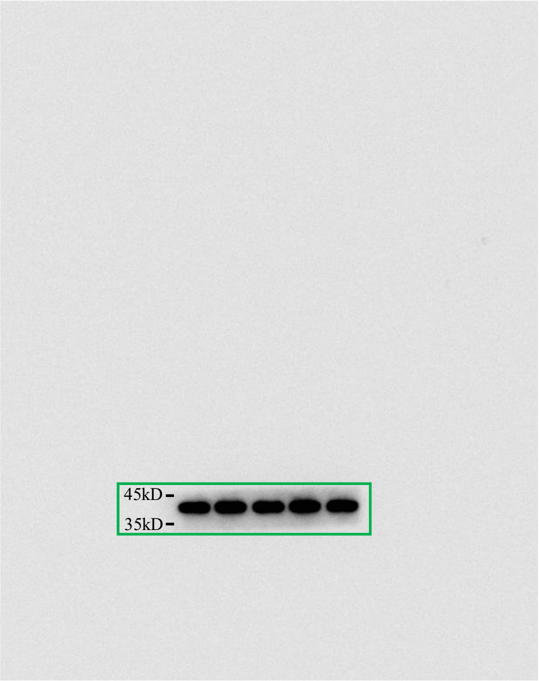

Supplementary Figure 8E U251 representative image

NTRK2-243aa

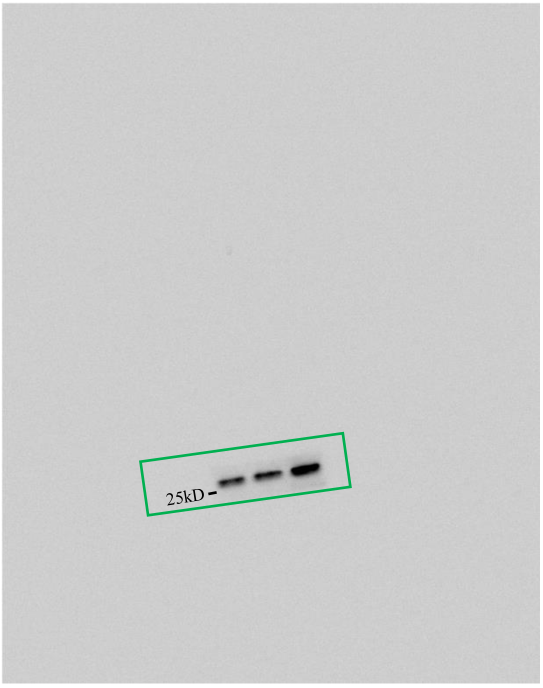

β-actin

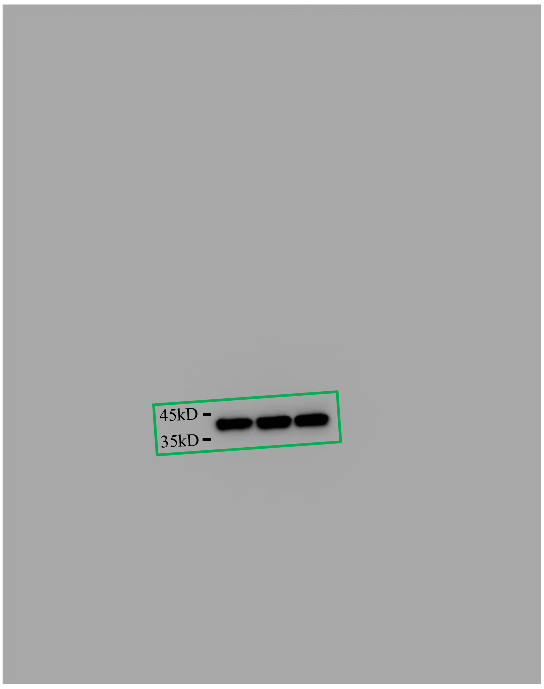

Supplementary Figure 8E U373 representative image

NTRK2-243aa

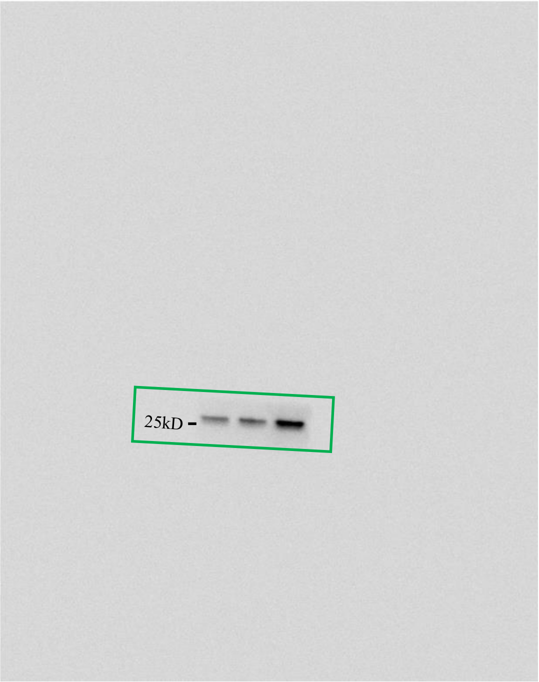

β-actin

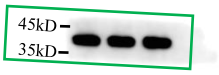

Supplementary Figure 8G U251 representative image

PAX5

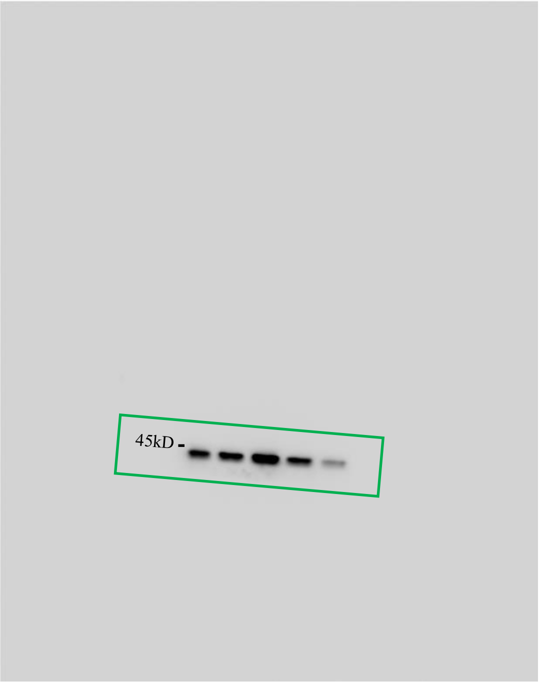

β-actin

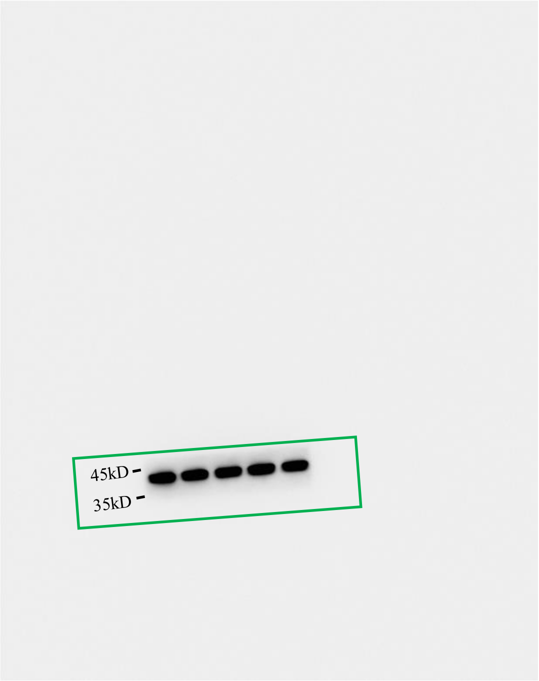

Supplementary Figure 8G U373 representative image

PAX5

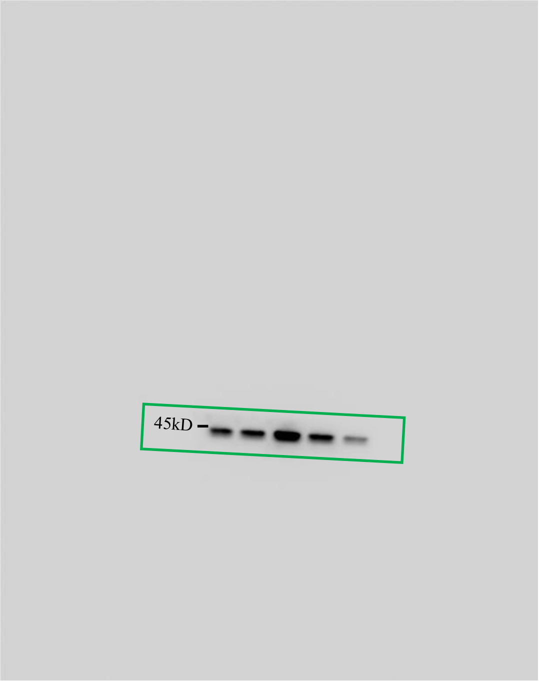

β-actin

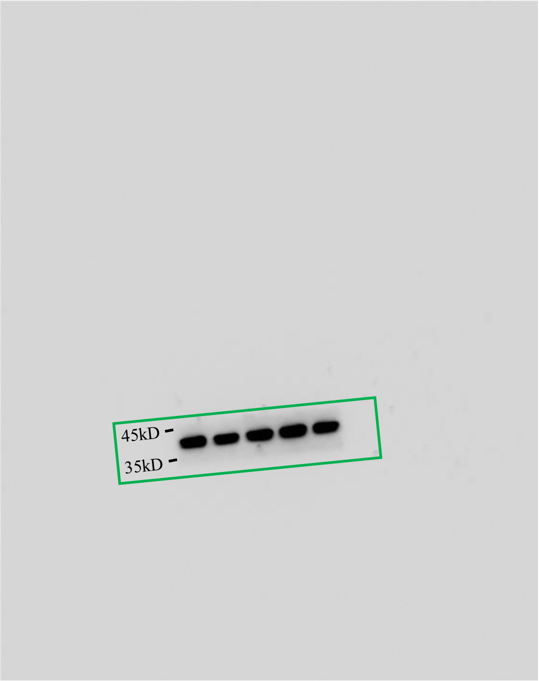

Supplement: Supplementary file 8 — supplementary file of western blot [file 41419_2022_5219_MOESM8_ESM.pdf]
